# Prevalence and mechanism of synergistic carboxylate-cation-water interactions in halophilic proteins

**DOI:** 10.1016/j.bpj.2023.05.011

**Published:** 2023-05-12

**Authors:** Hosein Geraili Daronkola, Ana Vila Verde

**Affiliations:** 1 Max Planck Institute of Colloids and Interfaces, Department of Theory & Bio-Systems, Potsdam, Germany

## Abstract

The cytoplasmic proteins of some halophilic organisms remain stable and functional at multimolar concentrations of KCl, i.e., under conditions that most mesophilic proteins cannot withstand. Their stability arises from their unusual amino acid composition. The most dramatic difference between halophilic and mesophilic proteins is that the former are rich in acidic amino acids. It has been proposed that one of the evolutionary driving forces for this difference is the occurrence of synergistic interactions between multiple acidic amino acids at the surface of the protein, the potassium cations in solution, and water. We investigate this possibility with molecular dynamics simulations, using high-quality force fields for the protein-water, protein-ion, and ion-ion interactions. We create a rigorous thermodynamic definition of interactions between acidic amino acids on proteins that can be used to distinguish between synergistic, noninteracting and interfering interactions. Our results demonstrate that synergistic interactions between neighboring acidic amino acids in halophilic proteins are frequent at multimolar KCl concentration. Synergistic interactions have an electrostatic origin, and are associated with stronger water-to-carboxylate hydrogen bonds than for acidic amino acids without synergistic interactions. Synergistic interactions are not observed in minimal systems of carboxylates, indicating that the protein environment is critical for their emergence. Our results demonstrate that synergistic interactions are neither associated with rigid amino acid orientations nor with highly structured and slow moving water networks, as had been originally proposed. Moreover, synergistic interactions can also be found in unfolded protein conformations. However, because these conformations are only a small subset of the unfolded state ensemble, synergistic interactions should contribute to the net stabilization of the folded state.

## Significance

X-ray crystallography and NMR studies have suggested that acidic amino acids in folded halophilic proteins interact synergistically with water and cations at molar KCl concentrations, stabilizing the folded protein. We provide, for the first time, an operational definition of synergistic interactions between acidic amino acids that enables their quantification in molecular simulations. This definition can easily be adapted to investigate synergistic interactions between other amino acids. The results confirm that synergistic interactions exist at high KCl concentration and should stabilize the folded protein. They have an electrostatic origin and are associated with strong water-to-carboxylate hydrogen bonds. The results suggest nevertheless that other mechanisms may contribute to the evolutionary driving force behind the abundance of acidic amino acids in halophilic proteins.

## Introduction

A few environments on Earth have physical-chemical properties far from the typical earthly environment; nevertheless, life thrives in these extreme environments as well. One striking example of such places is the Dead Sea, with its multi molar concentration of bromide and chloride salts, i.e., much higher than the typical salt concentration (≈0.7 mol·dm^−3^) in oceans. Microorganisms living at high salt concentrations are a specific type of extremophile; they are called halophiles (from the Greek word for “salt-loving”) (1). Halophilic microorganisms are mainly archea (2) and bacteria (3), but halophilic fungi, algae, protozoa, and multicellular eukaryotes also exist (4,5). To prevent rupture of their membrane due to the high concentration of salt in the environment, and therefore a high osmotic pressure, halophiles accumulate osmolytes inside their cytoplasm up to the same concentration as the external environment (1). For some halophiles, the main osmolyte is KCl; these halophiles boast cytoplasmic KCl concentrations in the multimolar range, far higher than the 0.15 mol·dm^−3^ that is typical of nonhalophilic organisms (termed mesophiles). Halophilic proteins (of halophiles where KCl is the main osmolyte) thus remain soluble, structurally stable, and functional (1,4,6,7,8) under conditions that proteins of mesophiles (i.e., mesophilic proteins) cannot typically tolerate (1,9,10,11,12).

Halophilic and mesophilic proteins differ in their amino acid composition (7). Revealing and quantifying how amino acid composition impacts protein-solvent, protein-ion, and protein-protein interactions so proteins remain stable and functional at high KCl concentration is important from a fundamental perspective, because halophilic environments are ancient and are candidates for proteogenesis (the origin of polypeptides) and abiogenesis (the origin of living systems) (13,14,15). The amino acids most abundant in halophilic proteins are part of the prebiotic set of amino acids; polypeptides formed from this set have the ability to fold when under halophilic conditions (13,14,15). Clarifying the physical-chemical mechanisms that result in structurally stable and functional proteins under halophilic conditions is thus critical for a broader understanding of evolution and of protein folding. It is also important from a biotechnological perspective, to develop methods to rationally determine the optimal and minimal modifications that must be introduced in mesophilic enzymes (e.g., for production of chiral drug molecules (16), for production of biofuels (17)) so they remain functional at high NaCl concentration. Enzymes with this ability are advantageous in a world where fresh water resources are increasingly scarce.

Mesophilic and halophilic proteins differ substantially in composition and structure. The most noticeable difference between them is that halophilic proteins are highly depleted of positively charged residues, and are highly enriched in negatively charged amino acids, especially aspartic acid, located on the surface of the protein (4,6,7,18,19). Halophilic proteins are thus on average substantially negatively charged. The physical mechanisms making up the evolutionary driving force for this characteristic are not yet well-understood. Multiple explanations have been proposed, as we described elsewhere (20); here we provide only a short summary. At high salt concentration, electrostatic interactions are strongly screened. A seemingly obvious explanation for the excess acidic amino acids in halophilic proteins is that a high net protein charge is necessary for sufficient electrostatic repulsion to prevent protein aggregation, i.e., to increase protein solubility (4,21). However, the fact that enzyme activity, structural stability, and association of subunits strongly depend on salt concentration beyond 0.5 mol·dm^−3^, even though charge screening is largely complete by this concentration, indicates that, while maintenance of solubility may well be one of their roles, acidic amino acids should have other roles as well (1). The *solvent-only stabilization* model claims that acidic amino acids, which have the highest water-binding ability of all amino acids (22), are necessary to keep the protein hydrated by competing with the ions in solution for available water (2,23). However, our previous simulation work (20) does not support that scenario. Our simulations indicate that the amount of hydration water for any given protein is barely affected by changing the KCl concentration from 0.15 to 2 mol·dm^−3^ (20).

In this work, we investigate another proposed explanation for the abundance of acidic amino acids in halophilic proteins: the *ion-solvent stabilization model*. This hypothesis claims that hydrated ion networks form around the surface of the folded protein, enabled by specific arrangements of carboxyl groups that attract hydrated potassium ions and which thus induce synergistic interactions between the acidic amino acids (4,24,25,26). This hypothesis has been described in qualitative terms only. Because analogous arrangements of acidic amino acids cannot form (or at least are not dominant) in the ensemble of unfolded protein structures, the folded structure of halophilic proteins is stabilized relative to the unfolded state (4,24,25,26). This hypothesis is supported by the presence of highly ordered protein hydration shells in some of the few halophilic proteins that have been crystallized (27). Synergistic hydrated ion networks would explain why some halophilic proteins have higher hydration levels than predicted based on their content in acidic amino acids alone, and why halophilic proteins bind larger amounts of salt than mesophilic proteins (4,24,25,26,28). Such an ordered solvation layer around the protein is proposed to function as a barrier that prevents aggregation of halophilic proteins at high salt concentration (4,24,25,26). This hypothesis was further supported by measurements of water translational dynamics in the cytoplasm of halophilic and nonhalophilic bacteria using quasielastic neutron spectroscopy, which were interpreted as indicating that water near halophilic proteins had extremely slow translational dynamics (29). However, this interpretation of the quasielastic neutron spectroscopy measurements was subsequently challenged by another experimental study (30), and by our previous simulation work, which demonstrated that the translational dynamics of water of hydration of mesophilic and halophilic proteins are quite similar (20).

We use simulations to determine whether synergistic water + potassium + carboxylate networks indeed arise in halophilic proteins and contribute toward the stability of the folded protein structure relative to the unfolded state. We use force fields for the carboxylate-potassium, carboxylate-amine, and carboxylate-water interactions previously optimized by us to correctly capture these interactions, a critical aspect of this work (20,31). With the optimized parameters, the models reproduce the KCl activity derivative of aqueous potassium acetate solutions up to $b_{{\text{KCH}}_{3}\text{COO}}=2$ mol·kg^−1^, the carboxylate-potassium distances visible in the crystal structure of a protein, the difference between the experimental hydration free energies of the carboxylate and the chloride ions, and the osmotic pressure of concentrated glycine solutions. Although multiple references to synergistic or cooperative effects involving acidic amino acids in halophilic proteins can be found in the literature, a quantifiable definition has not been offered. We thus start by defining synergistic and nonsynergistic (i.e., interfering) interactions between acidic amino acids in a precise and quantifiable manner. The minimum system in which synergistic interactions between acidic amino acids and the ions in solution can exist is that involving two neighboring amino acids. We investigate whether synergistic interactions exist between pairs of neighboring amino acids on halophilic proteins, and also in model systems composed of dimers or trimers of mimics of amino acid side chains. Our results make clear that synergistic interactions between neighboring acidic amino acids are indeed possible in proteins at high KCl concentration, and give insight into the physical mechanisms behind them.

## Methods

All simulations used the TIP3P water model (32), and modified versions of the AMBER ff14SB (33,34) force field for proteins and of the potassium and chloride parameters of Joung and Cheatham (35) for TIP3P water. The original force fields significantly overestimate the interaction between carboxylates and amines and between carboxylates and the potassium ion. To overcome these shortcomings, we modified the Lennard-Jones (LJ) interactions between carboxylate and water, carboxylate and amines, and carboxylate and potassium ions as described in our previous work (20,31).

### Free energy calculations

**Figure 1:**
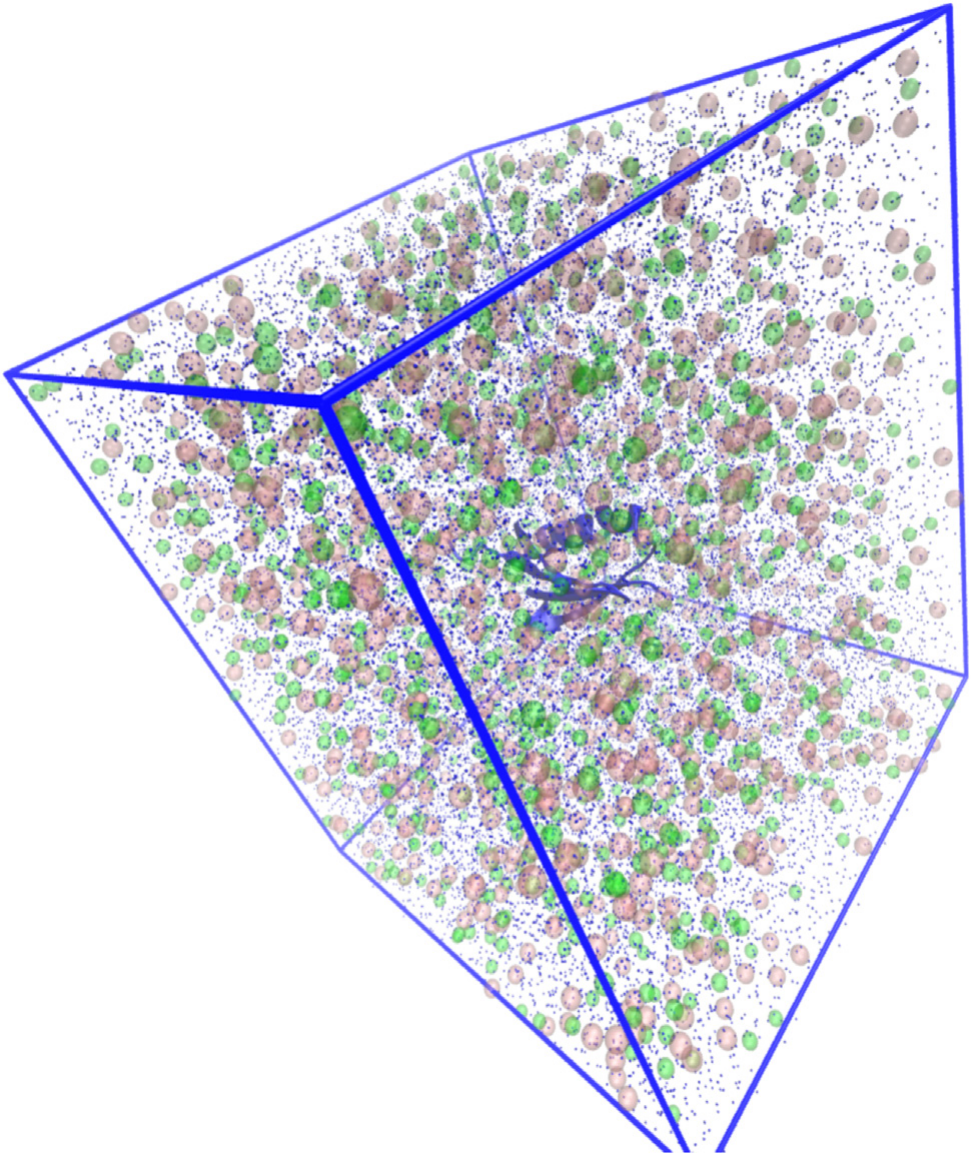
A snapshot of the simulation box of protein L in an aqueous solution of KCl at *b*_KCl_=2 mol·kg^−1^. Protein L, dark blue; K^+^, transparent pink spheres; Cl^−^, transparent green spheres; oxygen atoms of water molecules, blue dots. To see this figure in color, go online.

Free energies associated with the mutation of acidic residues—Asp (D) or Glu (E)—to their neutral counterparts—Asn (N) or Gln (Q), respectively—were calculated using thermodynamic integration as implemented in the pmemd GPU engine of the AMBER 18 molecular dynamics package (36), using a dual-topology approach and soft core potentials. Calculations were performed for halophilic proteins with Protein Data Bank identifying codes (PDB) PDB: 1DOI (2Fe-2S ferredoxin from *H. marismortui* (23)), PDB: 2KAC (protein L mutant Kx6E (37)), and PDB: 2ITH (dihydrofolate reductase from *H. volcanii* (38)). Folded proteins PDB: 1DOI and PDB: 2KAC were simulated at two different salt concentrations: *b*_KCl_ =0.15 mol·kg^−1^, corresponding to mesophilic conditions (the control situation), and *b*_KCl_ =2 mol·kg^−1^, corresponding to halophilic conditions. The folded dihydrofolate reductase and the unfolded protein L were simulated at the higher salt concentration to increase the number and diversity of systems under halophilic conditions, for which synergistic effects have been proposed to occur. Given the very high computational cost of these calculations, these two systems were not simulated at low salt concentration. The starting structure of each folded protein for the free energy calculations was the last saved configuration of 1 *μ*s production runs at the desired salt concentration, reported in our previous publication (20). The starting structure for the simulations with the unfolded protein L was obtained as described in supporting material, section 6. The simulation boxes (Fig. 1) are cubic, with edge length ≈100 Å.

To choose appropriate pairs of acidic amino acids for the mutation study, we first selected residues with a minimum solvent-accessible surface area of 50 Å^2^ using the package *chimera* (39), thus ensuring they were solvent exposed. Of this set, we selected amino acid pairs for which the distance between their carboxylate carbons was below 7 Å at t=0.

Simulations were performed in three steps, described in Eq. 1 for the mutation of a generic amino acid X into amino acid Y. In the equation, *a* denotes the position of the residue in the amino acid sequence. Y_*a*_^0^ and X _*a*_^0^ indicate nonphysical forms of the amino acid where all atomic charges are set to zero. The free energy of mutation, $\Delta G_{XaY}$, is equal to the sum of the three partial terms shown in Eq. 1. This free energy reflects only the different nonbonded interactions of the initial- and final-state amino acids with the solvent (water and ions) and the surrounding protein.(1)

Complete details of the free energy simulations are given in supporting material, section 2.1; here we summarize only the most relevant aspects. The protein backbone atoms were restrained to their initial position using a harmonic restraint with a force constant of 35 kcal·mol^−1^·Å^−2^. Doing so is indispensable to obtain reproducible values of $\Delta G_{XaY}$ for each mutation. Our tests, discussed in supporting material, section 3, indicate that, without backbone restraints, small conformational changes away from the mutation site affect the value of $\Delta G_{XaY}$ by a few kcal·mol^−1^. Changes of this magnitude are similar to those that may arise from the synergistic effect, and would thus make results impossible to interpret. The introduction of backbone restraints eliminates this difficulty. We emphasize that the energy of the restraints does not contribute to ∂V/∂λ, and therefore does not contribute to the mutation free energy.

The Python tool *alchemical-analysis* (40) was used to calculate the free energy difference for each of the steps in Eq. 1. This tool calculates multiple free energy estimates, using different methods, from the simulation data. In all cases, the first 1 ns of the production simulations was ignored and considered as equilibration time. The values reported here were estimated integrating ∂V/∂λ using a natural cubic spline (“TI-3”). A detailed analysis of computational accuracy and choice of the best estimator for our system is given in supporting material, section 2.1.1.

### Potential of mean force calculations

**Figure 2:**
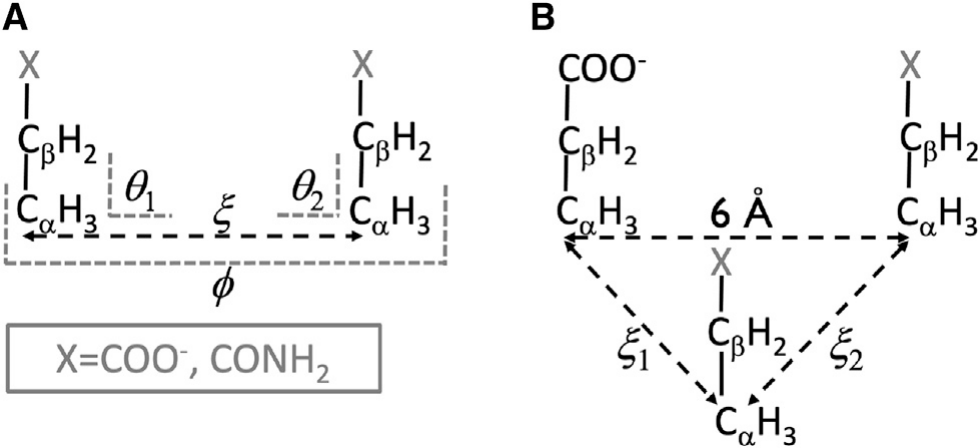
Systems and collective variables used in the potential of mean force calculations; X = COO^−^ for the model aspartate side chain and X = CONH_2_ for the model asparagine side chain. (*A*) Two-body systems, showing the reaction coordinate ξ and the restrained angles ($\theta_{1}$ and $\theta_{2}$) and dihedral (φ). (*B*) Three-body systems, showing the reaction coordinates $\xi_{1}$ and $\xi_{2}$; angle and dihedral restraints (not shown) analogous to those applied in the two-body system were used to keep the side chains parallel to each other and perpendicular to the plane defined by the three C_*α*_s.

To generate minimal models of the side chains of aspartate and of asparagine, we replaced the amino (${{\text{–NH}}_{3}}^{+}$) and carboxylate (${{\text{–CO}}_{2}}^{-}$) functional groups attached to the C_*α*_ of the zwitterionic form of the amino acids by two hydrogen atoms, to form a -C_*α*_H_3_ group. The three hydrogen atoms attached to the C_*α*_ were given identical charges so that the net charge of the side chain model is zero (for asparagine) or −1 (for aspartate). Finally, the van der Waals (vdW), bond, angle, and dihedral parameters for interactions involving the newly added hydrogen atoms and the rest of the side chain atoms were given the same values as those of the standard hydrogen bonded to C_*α*_ in the respective amino acid. The side chain models are shown in Fig. 2.

All umbrella sampling simulations from which the potential of mean force (PMF) curves were obtained were performed using the GROMACS 2020 simulation package (41,42). Complete simulation details are given in supporting material, section 2.1; here we summarize only the most relevant simulation choices. The two-body PMFs were calculated as a function of the distance, ξ, between the C_*α*_ of the side chains. For all cases, the side chains were kept approximately coplanar by restraining the dihedral φ defined by atoms $C_{\beta 1}$-$C_{\alpha 1}$-$C_{\alpha 2}$-$C_{\beta 2}$ of the two amino acids (denoted by the subscripts 1 and 2) to $\varphi =0^{\circ }$. Calculations were performed for different relative orientations of the side chains, enforced by restraining the angles $\theta_{1}$ and $\theta_{2}$ to different values; $\theta_{1}$ is defined by atoms $C_{\beta 1}$-$C_{\alpha 1}$-$C_{\alpha 2}$; $\theta_{2}$ is defined by atoms $C_{\alpha 1}$-$C_{\alpha 2}$-$C_{\beta 2}$. The collective variables used in the two-body PMF calculations are illustrated in Fig. 2 *A*. The two-body PMF was calculated for three different systems—D-D, N-N, and D-N—in explicit water and KCl at *b*_KCl_ = 0.15 and 2 mol·kg^−1^.

The three-body PMF was calculated as a function of the distances, $\xi_{1}$ and $\xi_{2}$, of one amino acid to the other two, as illustrated in Fig. 2 *B*. The C_*α*_s of two of the amino acids are restrained to fixed positions in space so they are on average 6 Å apart. The position of the C_*α*_ of the third amino acid is governed by the restraints applied to $\xi_{1}$ and $\xi_{2}$. Six angular and three dihedral restraints (not shown in Fig. 2 *B*) analogous to those employed in the two-body system were used to keep the side chains approximately perpendicular to the plane defined by the three C_*α*_s, by setting the angle equilibrium values to $90^{\circ }$ and the dihedral ones to $0^{\circ }$. PMFs were calculated for three different systems—D-D--D, D-D--N, and D-N--N—in explicit water and KCl at *b*_KCl_ = 2 mol·kg^−1^. The WHAM (43) analysis program implemented in GROMACS was used to compute the PMF curves from the output of the umbrella simulations.

## Results

### Defining and quantifying synergistic interactions between acidic amino acids

As summarized above, the ion-solvent stabilization model claims that synergistic interactions between spatially close acidic amino acids, mediated by hydrated potassium cations, stabilize folded halophilic proteins at high KCl concentrations (24,26,44). To investigate this effect in simulations, a precise and quantifiable definition of synergistic interactions between acidic amino acids is necessary. The smallest system in which synergistic interactions between acidic amino acids can exist consists of two acidic residues. With this in mind, we designed a simulation protocol to assess the existence and magnitude of these synergistic interactions by a difference of mutation free energies. A pair of solvent-exposed acidic amino acids in a protein, in positions *a* and *b* of the amino acid sequence, is selected. We first calculate the free energy of mutating of one of them to the neutral natural amino acid that most closely resembles it: aspartate (D) is mutated to asparagine (N) and glutamate (E) to glutamine (Q). This mutation is schematically represented as:

(2) $$\left(-\right)X_{a}\overset{\Delta G_{\left(-\right)XaY}}{\to }\left(-\right)Y_{a}$$

Equation 2 indicates that amino acid X in position *a* is mutated to amino acid Y; the minus sign in parentheses (−) emphasizes that this mutation is done in the presence of a particular negative neighbor in position *b*. The free energy $\Delta G_{\left(-\right)XaY}$ contains only the contribution of the interactions between the environment (i.e., the water, ions in solution, and the rest of the protein) and the mutated residues to the mutation free energy, as detailed in the methods. The structure and size of the neutral amino acid Y (Q or N) are very similar to that of X (E or D) as can be seen in Fig. S1, so the initial and final states are expected to have similar vdW interactions with their environment, i.e., these differences will contribute minimally to $\Delta G_{\left(-\right)XaY}$. Our results below confirm that this assumption holds. $\Delta G_{\left(-\right)XaY}$ is thus dominated by electrostatic interactions with the solvent (i.e., water + ions). These interactions are much more favorable for charged amino acids than for uncharged ones (45), so this free energy is positive (i.e., the mutation is unfavorable). If solvent-mediated synergistic electrostatic interactions exist between the two acidic amino acids, they will be present in the initial state but not the final state of the system, and will thus affect the value of $\Delta G_{\left(-\right)XaY}$.

In a second step, we calculate the free energy associated with the same mutation, but now for a modified version of the protein where D or E in position *b* was replaced by N or Q, as appropriate, before the calculation is performed. This second mutation is schematically represented as

(3) $$\left(0\right)X_{a}\overset{\Delta G_{\left(0\right)XaY}}{\to }\left(0\right)Y_{a}$$

where (0) emphasizes that the neighboring amino acid is electrically neutral.

The difference

(4) $$\Delta \Delta G=\Delta G_{\left(0\right)XaY}-\Delta G_{\left(-\right)XaY}$$

gives direct insight into the presence or absence of synergistic interactions between amino acids, and quantifies the magnitude of this effect. If amino acids *a* and *b* interact very weakly (e.g., if they are spatially distant), ΔΔG=0. If they are nearby, conventional understanding would suggest that two charged amino acids repel each other. Moreover, this repulsion should be more intense than any interactions between two neutral amino acids or between one neutral and one charged amino acid (in the end state). To first approximation, the electrostatic interaction between two neutral amino acids is a dipole-dipole interaction, which decays as $1/r^{3}$, where *r* is the distance between the two dipoles; likewise, the electrostatic interaction between a neutral and a charged amino acid is a charge-dipole interaction, which decays as $1/r^{2}$. In contrast, charge-charge interactions decay as 1/r. In this conventional picture, the (−) X*a*Y mutation should be less unfavorable than the (0) X*a*Y one, i.e., $\Delta G_{\left(-\right)XaY}$ < $\Delta G_{\left(0\right)XaY}$, so ΔΔG>0; we call these interactions repulsive or interfering.

A negative value of ΔΔG indicates a synergistic interaction: it is harder to mutate an acidic amino acid to a neutral one in the presence of a nearby acidic residue than when the neighbor is not acidic. Our results below will show that this synergistic interaction indeed stems from the interactions between the two acidic amino acids.

Practically, to account for the uncertainty in the calculated values of ΔΔG, we consider that negligible interactions exist if |ΔΔG|<0.5 kcal·mol^−1^. Significant interfering interactions occur if ΔΔG>0.5 kcal·mol^−1^ and significant synergistic interactions exist if ΔΔG<−0.5 kcal·mol^−1^.

We note that other approaches to assess whether neighboring acidic amino acids have synergistic interactions are in principle possible. For example, mutating charged amino acids to their protonated versions should lead to results comparable with ours, because protonating the acid minimally perturbs its LJ interactions with its neighbors. The definition proposed here (Eqs. 2, 3, and 4) has the advantage of being more intuitive because it relies on protonation states realistically accessible at the same pH. Quantitative definitions relying on mutations of charged amino acids to purely hydrophobic ones, however, will likely introduce larger changes also in the LJ interactions between them, making it harder to isolate the electrostatic effect that is thought to be at the source of synergistic interactions.

### Free energies of D → N and E → Q mutations in folded proteins

We calculated ΔΔG for selected pairs of acidic residues close to each other (the distance between the *a* and *b* carboxylate carbons is <7 Å at t=0) on the surface of three halophilic proteins: halophilic ferredoxin (PDB: 1DOI), halophilic protein L (PDB: 2KAC), and halophilic dihydrofolate reductase (PDB: 2ITH). These proteins were selected because they differ in net electric charge and size. Protein L has a net charge of −15*e* but is substantially smaller (only 64 amino acids) than either the halophilic dihydrofolate reductase (162 amino acids) or the halophilic ferredoxin (128 amino acids). The halophilic dihydrofolate reductase is substantially less charged (−15*e*) than the halophilic ferredoxin (−29*e*). Their diverse characteristics thus enable us to draw general conclusions regarding the importance of synergistic interactions between acidic amino acids in halophilic proteins. Given the high computational cost of these calculations, we investigated a smaller set of amino acid pairs in two of the proteins at a low salt concentration (*b*_KCl_ = 0.15 mol·kg^−1^) and a larger set of pairs in the three proteins at the highest salt concentration (*b*_KCl_ = 2 mol·kg^−1^), for which the force fields yield reliable results.

**Figure 3:**
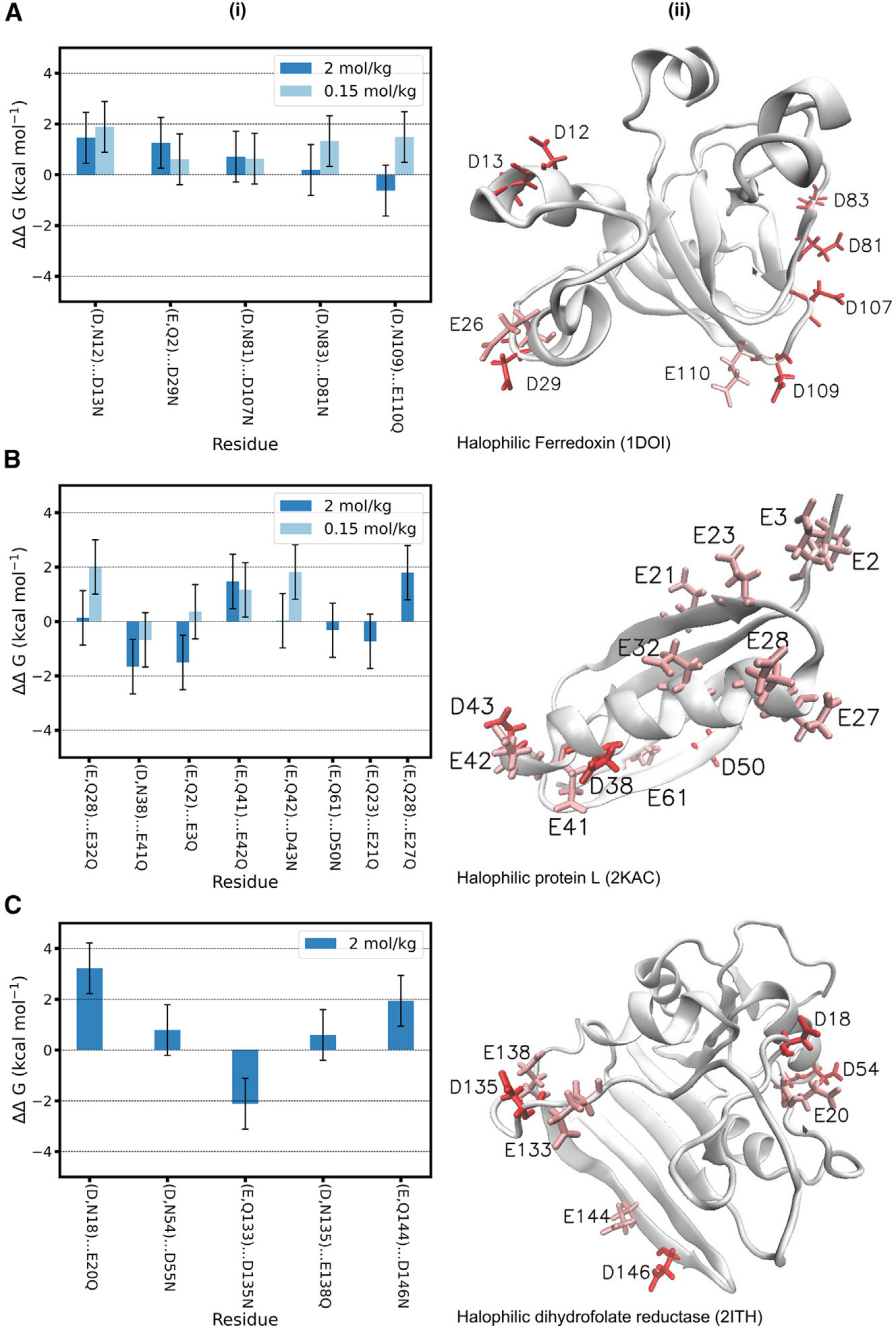
Interactions between pairs of acidic amino acids in folded halophilic proteins. (*i*) Change in free energy of mutation (ΔΔG±1.0 kcal·mol^−1^; Eq. 4) for selected pairs of amino acids of the indicated proteins. The ΔΔG values are compiled in Tables S4–S6. The error bars are calculated using error propagation, using as input the standard error of the mean of each ΔG value estimated from five independent calculations for one of the protein sites as described in supporting material, section 3. (*ii*) Structure of each protein during the free energy calculation simulations; the acidic amino acids in (*i*) are displayed in red (D, aspartic acid) and in pink (E, glutamic acid). To see this figure in color, go online.

Fig. 3 shows the calculated values of ΔΔG in (*i*). The position of the residues of each pair is shown in (*ii*). The amino acid pairs and the mutations performed are indicated using condensed notation: e.g., (D,N36) … E41Q indicates that the glutamate in position 41 of the amino acid sequence is mutated to glutamine; the vicinal amino acid is in position 36 and is either an aspartate (when calculating $\Delta G_{\left(-\right)E41Q}$) or an asparagine (when calculating $\Delta G_{\left(0\right)E41Q}$). Fig. 3 *A* and *B* shows values of ΔΔG for the two proteins investigated at low KCl concentration in light blue. Most values of ΔΔ G are positive, but vary substantially (between 0.5 and 2 kcal·mol^−1^) depending on the pair of amino acids being investigated. These results are qualitatively in line with the conventional expectation that, at low salt concentration, neighboring acidic amino acids should repel. Synergistic interactions between acidic amino acids at low KCl concentration are only observed for one of the sites ((D,N38) … E41Q of protein L) and are very weak (ΔΔG=−0.5 kcal·mol^−1^).

At high KCl concentration, however, the picture that emerges is very different: significant synergistic interactions are observed in 5 out of the 18 pairs of amino acids investigated (Fig. 3 *A*–*C*), i.e., for those with ΔΔG<−0.5 kcal·mol^−1^, as described in the previous subsection. Moreover, synergistic interactions have similar magnitude (−0.5 to −2 kcal·mol^−1^) as the observed repulsive interactions (positive ΔΔG cases) in other amino acid pairs at the same concentration; in other words, the synergistic effect, when present, is of considerable magnitude. In half of the pairs investigated at high salt concentration, we observe interfering (i.e., repulsive) interactions. The values of positive ΔΔG are clearly lower than those at low KCl concentration for the same pairs, reflecting the expected higher electrostatic shielding brought by the more concentrated electrolyte solution. This result illustrates the limitations of mean field descriptions of electrostatic interactions for short distances: mean field calculations predict that the electrostatic interaction between acidic amino acids at *b*_KCl_ = 2 mol·kg^−1^ is fully shielded for distances larger than 2 Å, i.e., ΔΔG should be very close to zero for the pairs we selected. In our calculations, full shielding is only observed for 3 of the 18 pairs tested.

The results in Fig. 3 demonstrate that a synergistic interaction between neighboring acidic amino acids is possible, and in fact occurs frequently at high salt concentration: in our data set, we observe it in ≈1/3 of the amino acid pairs tested. The solvent-mediated (i.e., water- and ion-mediated) interaction between the amino acids in these pairs thus contributes to the stabilization of the folded protein structure. Moreover, for 1/6 of the amino acid pairs tested at high salt concentration, the interaction between the neighboring amino acids was reduced to zero: those pairs neither stabilize nor destabilize the folded protein structure. For half of the pairs investigated at high salt concentration, however, we observe positive values of ΔΔG. Overall, our results suggest that the ion-solvent stabilization effect exists. The fact that a synergistic interaction is more frequent at high salt concentration confirms that it is mediated by the ions and water. Nevertheless, this effect contributes only to a limited extent to the stabilization of the folded structure of halophilic proteins, at least at *b*_KCl_ = 2 mol·kg^−1^. It is possible that the ion-solvent stabilization effect becomes more intense and more likely at even higher salt concentrations, but at present this possibility cannot be investigated using molecular simulations because force fields that remain accurate up to the solubility limit of the salt do not exist.

### Free energies of D → N and E → Q mutations in the unfolded halophilic protein L

**Figure 4:**
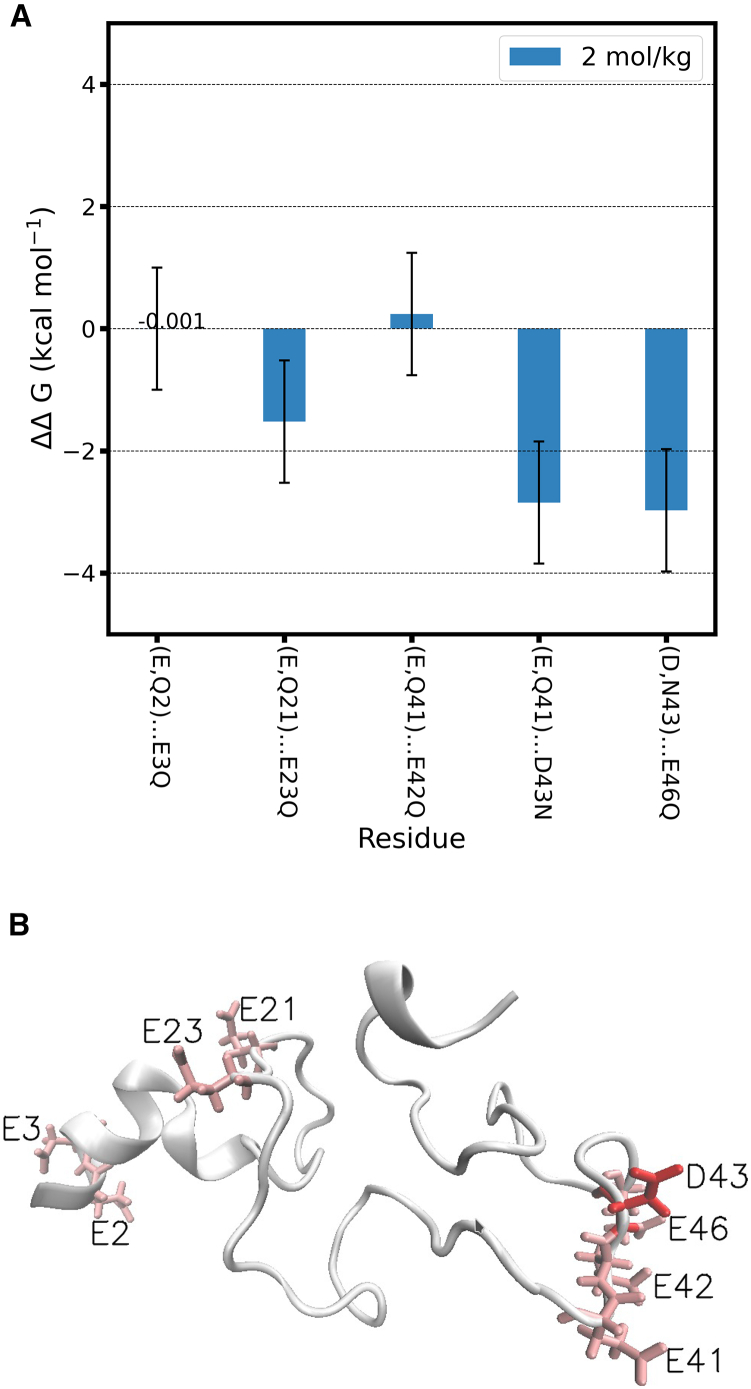
Interactions between pairs of acidic amino acids in the unfolded halophilic protein L at *b*_KCl_ = 2 mol·kg^−1^. (*A*) Change in free energy of mutation (ΔΔG±1.0 kcal·mol^−1^; Eq. 4) for selected pairs of amino acids. The ΔΔG values are compiled in Table S7. The error bars are calculated using error propagation, using as input the standard error of the mean of each ΔG value estimated from five independent calculations for one of the protein sites as described in supporting material, section 3. (*B*) Unfolded conformation of protein L used in the free energy calculations; the acidic amino acids in (*A*) are displayed in red (D, aspartic acid) and in pink (E, glutamic acid). To see this figure in color, go online.

We next tested whether synergistic interactions between acidic amino acids in unfolded proteins are possible: we calculated ΔΔG for multiple pairs of spatially close acidic amino acids belonging to an unfolded structure of halophilic protein L, and compared the frequency of occurrence and magnitude of synergistic effects relative with those observed for the same protein in the folded state. Protein L was selected because, of the three proteins investigated in the folded state, it had the most frequent synergistic interactions. The unfolded configuration was obtained from a replica exchange molecular dynamics (REMD) simulation, as described in supporting material, section 6. The REMD simulation could not sample the full ensemble of unfolded configurations; rather, it sampled a subset of configurations where the protein is denatured but collapsed. For our purpose this behavior is not an impediment because synergistic interactions, if present in the unfolded state, should be more likely when pairs of acidic amino acids are sufficiently close. Theseus (46) was used to find the configuration with the structure most similar to the average structure in the REMD trajectory at 298 K. This configuration is shown in Fig. 4. The backbone of the structure was restrained during the free energy calculations, similarly to the calculations done for the folded proteins.

Fig. 4*A* shows the ΔΔG values for the denatured structure of protein L at *b*_KCl_ = 2 mol·kg^−1^. Surprisingly, almost all of the pairs of amino acids show synergistic interactions, and the value of ΔΔG is more negative in some cases than the synergistic cases in the folded structure of the same protein. These results do not support the scenario that synergistic effects between pairs of amino acids are enabled by specific amino acid configurations exclusive to the folded protein structure (44). On the contrary, it seems that pairs of acidic residues assume relative distances and conformations leading to synergistic interactions very easily in denatured conformations of halophilic protein L.

### Mechanism behind the synergistic effect

#### Negative values of ΔΔG result from synergistic electrostatic interactions between acidic amino acids

To further understand the mechanism leading to the synergistic effect, we examined the three contributions to ΔΔG along the mutation path. The free energy associated with a generic mutation XaY is calculated in three steps

(5) $$\Delta G_{\text{XaY}}=\Delta G_{\text{decharge}}+\Delta G_{\text{vdW}}+\Delta G_{\text{charge}}$$

following the thermodynamic cycle shown in Eq. 1. The term $\Delta G_{\text{decharge}}$ is the free energy change associated with decharging amino acid X, i.e., setting all atomic charges of this residue to zero. The term $\Delta G_{\text{vdW}}$ corresponds to mutating the decharged residue $X^{0}$ into the decharged residue $Y^{0}$; this term includes only the contributions of the changes in the LJ potentials of the residue to the interactions with its environment and is often termed the vdW contribution. The final term, $\Delta G_{\text{charge}}$, is the free energy change associated with reinstating the atomic charges of amino acid Y. The values of ΔΔG can thus be decomposed into decharging, vdW, and charging contributions as:

(6) $$\Delta \Delta G_{\text{decharge}}=\Delta G_{\left(0\right)\text{decharge}}-\Delta G_{\left(-\right)\text{decharge}}$$

(7) $$\Delta \Delta G_{\text{vdW}}=\Delta G_{\left(0\right)\text{vdW}}-\Delta G_{\left(-\right)\text{vdW}}$$

(8) $$\Delta \Delta G_{\text{charge}}=\Delta G_{\left(0\right)\text{charge}}-\Delta G_{\left(-\right)\text{charge}}$$

**Figure 5:**
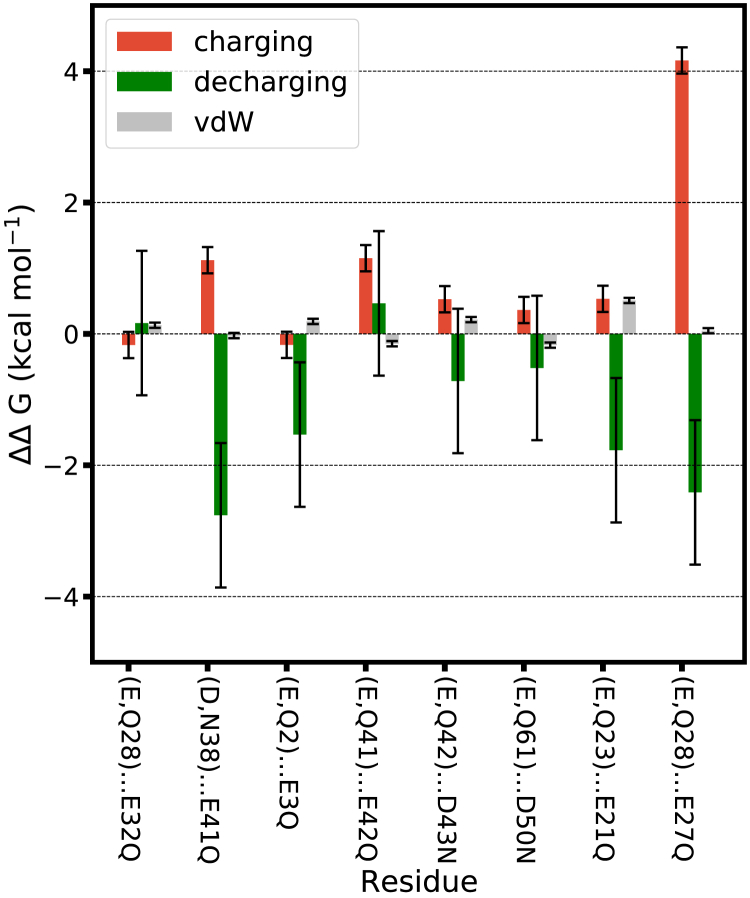
Components of ΔΔG for the indicated pairs of amino acids of the halophilic protein L at *b*_KCl_ = 2 mol·kg^−1^. $\Delta \Delta G_{\text{charge}}\pm 0.2$ kcal·mol^−1^, $\Delta \Delta G_{\text{decharge}}\pm 1.1$ kcal·mol^−1^, and $\Delta \Delta G_{\text{vdW}}\pm 0.04$ kcal·mol^−1^ (Eqs. 6, 7, and 8). The error bars are standard errors of the mean, estimated from 5 independent calculations for one of the protein sites as described in supporting material, section 3. The sum of the components for each amino acid pair equals the values shown in Fig. 3*Bi*). To see this figure in color, go online.

These components are shown in Fig. 5 for the folded halophilic protein L at *b*_KCl_ = 2 mol·kg^−1^. The $\Delta \Delta G_{\text{vdW}}$ component takes both positive and negative values, but its absolute value is always below ≈0.5 kcal·mol^−1^. The vdW component of the mutation free energy is thus essentially independent of the identity of the vicinal amino acid. The sign of ΔΔG is thus determined by the balance between the charging and the decharging steps. The charging step, which introduces the atomic charges in the final (neutral) amino acid, predominantly has a positive contribution to ΔΔG. In contrast, the decharging step, which removes the atomic charges in the initial (negative) amino acid, negatively contributes to ΔΔG.

This result confirms that a negative ΔΔG is associated with the decharging step. A negative value of $\Delta \Delta G_{\text{decharge}}$ could arise either from 1) unexpected *stabilizing* electrostatic interactions between the two vicinal acidic amino acids, or between 2) *destabilizing* electrostatic interactions between vicinal acidic and neutral amino acids. As discussed in detail in supporting material, section 5, the first possibility is the one at play here.

### Synergistic interactions are not observed in minimal systems of acidic amino acids

Do synergistic interactions between acidic amino acids occur in minimal systems, i.e., outside of a protein environment? Does the synergistic effect occur for particular distances or relative orientations? To answer these questions, we characterized the thermodynamics of the interaction of minimal systems mimicking the side chains of aspartate and of asparagine by calculating the PMF as a function of their distance. In the minimal systems, the carbon corresponding to the C_*α*_ in the full amino acid now forms a CH ${}_{3}$ group; that carbon and the hydrogen atoms directly bonded to it have modified charges, so the nominal charge of the side chain is the same as that of the original amino acid. For conciseness, we refer to the amino acid models as side chains, and by the names (aspartate or asparagine) and one-letter code (D or N) of the corresponding amino acids.

**Figure 6:**
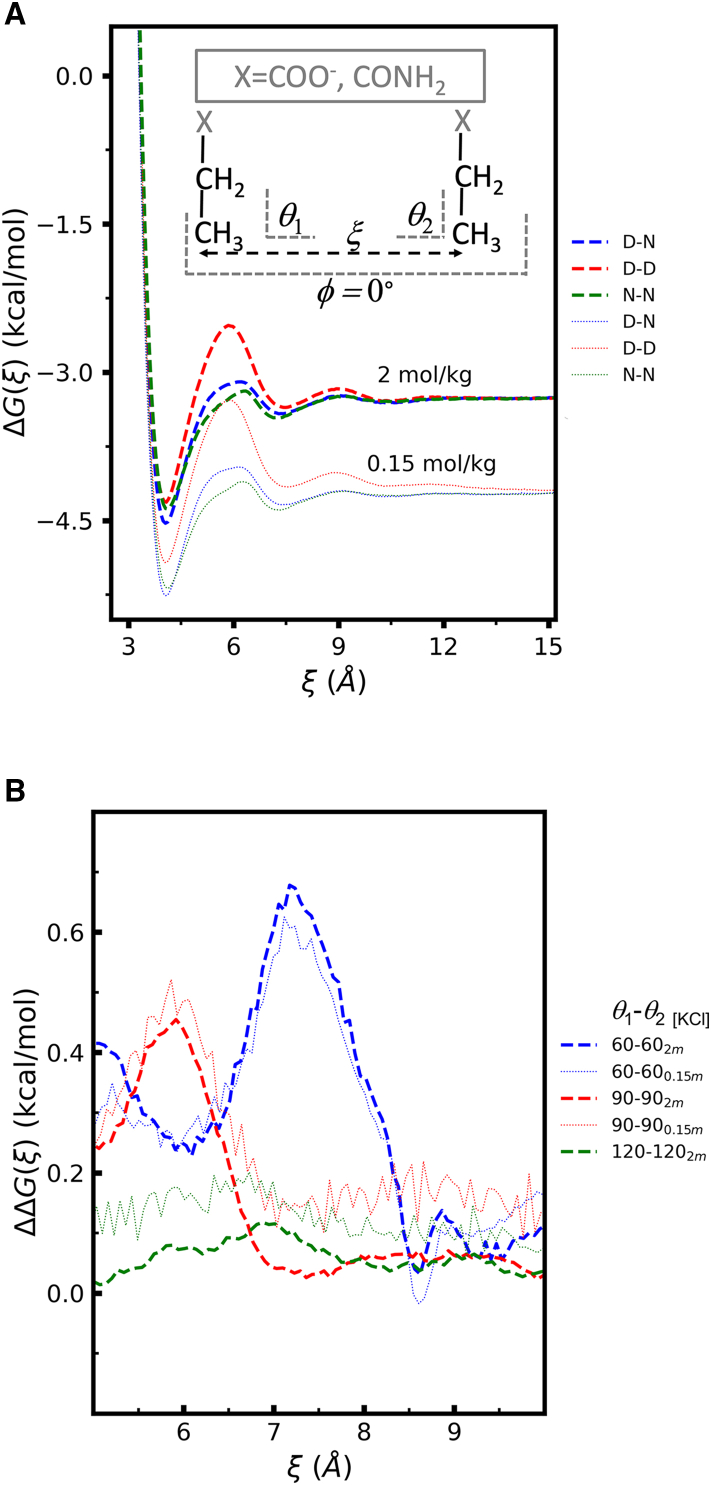
Potential of mean force as a function of C ⋯α C_*α*_ distance, for different orientations of pairs of side chain mimics, at *b*_KCl_ = 0.15 mol·kg^−1^ (0.15m) and *b*_KCl_ = 2 mol·kg^−1^ (2m). (*A*) PMF for the indicated pairs of amino acid side chains, for parallel side chains (restraint angles $\theta_{1}=\theta_{2}=90^{\circ }$; $\varphi =0^{\circ }$). The ideal gas entropic term ($-2k_{B}T\;\ln\left(\xi \right)$) is not included in the curves. (*B*) ΔΔG(ξ) (Eq. 9) for different relative orientations ($\theta_{1}$ and $\theta_{2}$; $\varphi =0^{\circ }$) of the side chains, at $b_{\text{KCl}}=0.15$ mol·kg^−1^ (0.15m) or $b_{\text{KCl}}=2$ mol·kg^−1^ (2m). To see this figure in color, go online.

Fig. 6*A* shows the PMF as a function of C ⋯α C_*α*_ distance (ξ) for the side chain pairs D-D, N-N, and D-N, at *b*_KCl_ = 0.15 mol·kg^−1^ and at *b*_KCl_ = 2 mol·kg^−1^, for the case where the side chains are parallel to each other and perpendicular to ξ. The curves are shifted along the *y* axis for ease of viewing, so the absolute values of ΔG(ξ) have no physical meaning. Differences between the three curves at each salt concentration, or within each curve, are physically meaningful. The D-N and N-N interactions at large distances can be assumed to be zero; for this reason, the curves for the D-N and N-N pairs at each salt concentration were shifted along the *y* axis to coincide at ξ=15 Å. The interaction energy between the D-D pair at ξ=15 Å at each salt concentration was estimated analytically as the Coulomb interaction between two point charges. Each D-D curve was shifted so that the difference $\Delta G_{D-D}(15$ Å $)-\Delta G_{N-N}(15$ Å) reproduces the analytical estimate for the respective salt concentration. At both concentrations, the maximum repulsion experienced by two negative side chains occurs at ξ≈ 6 Å, and is weak (<1 kcal·mol^−1^). The D-N and N-N pairs have negligible interaction energy down to ξ≈ 5 Å, as expected because charge-dipole and dipole-dipole interactions are short range and are screened at the salt concentrations considered. Below ξ≈ 4.5 Å, the ${CH}_{3}$ and ${CH}_{2}$ groups of the side chains are very close, whereas the terminal atoms point away from each other, as the representative configuration shown in Fig. S10 illustrates. As a result of this preferential configuration, attractive LJ interactions dominate over electrostatics, giving rise to a minimum in the PMF in all three cases (calculations shown in supporting material, section 7). Results (not shown) for other relative orientations of the side chains have the same qualitative features; the main differences are observed in the position and height of the repulsive maximum of the D-D pair. PMF curves calculated based on the distance between the terminal (i.e., the γ) carbons of the side chain show similar characteristics, as illustrated in Fig. S11 for the D-D pair.

To assess whether a synergistic effect can arise from side-chain pairs, we computed the quantity ΔΔG(ξ) from the PMF curves:

(9) $$\Delta \Delta G\left(\xi \right)=\left[\Delta G{\left(\xi \right)}_{N-N}-\Delta G{\left(\xi \right)}_{N-D}\right]-\left[\Delta G{\left(\xi \right)}_{D-N}-\Delta G{\left(\xi \right)}_{D-D}\right]=\Delta G{\left(\xi \right)}_{D-D}+\Delta G{\left(\xi \right)}_{N-N}-2\times \Delta G{\left(\xi \right)}_{D-N}$$

The subscripts indicate the pair of side chains for each PMF. The quantity ΔΔG(ξ) is analogous to the ΔΔG calculated for pairs of acidic amino acids on proteins (Figs. 3 and 4) but gives insight into cooperativity as a function of the distance between the side chains. Fig. 6 *B* shows ΔΔG(ξ) for both salt concentrations, for side chains at different relative orientations. The value and position of the maxima of each curve depend strongly on relative orientation. Nevertheless, this quantity never assumes negative values, indicating that a synergistic effect does not occur in this two-body system. Could it be that a synergistic effect arises in minimal systems with more side chains? To answer this question we performed an analogous PMF study for a system of three side chains. The results, presented in supporting material, section 8, indicate that a synergistic effect does not occur in that system either.

The absence of the synergistic effect in the two-body and three-body systems suggests that synergistic interactions between acidic amino acids do not result solely from water- and salt-mediated interactions between the acidic amino acids, but are also enabled by particular local protein environments.

### The synergistic effect does not require particular amino acid orientations

The large variation observed in the values of ΔΔG (−3<ΔΔG (kcal·mol^−1^) <+3) shown in Figs. 3 and 4 indicates that solvent-mediated interactions (synergistic or repulsive) between charged amino acids at high salt concentration are strongly affected by local protein composition and local structure. Obvious correlations between the sign or magnitude of ΔΔG and the secondary structural motifs in which the amino acids of the pair are located or the identity of other neighboring amino acids are not present. Moreover, in our data set the frequency of synergistic interactions differs strongly between proteins: they occur more frequently in protein L than for the other two proteins. Because the data set is small, it is at present unclear whether these differences between the proteins are significant. Clarifying these aspects will require their systematic study through a substantially larger data set of mutation free energy calculations, which is beyond what is currently possible.

**Figure 7:**
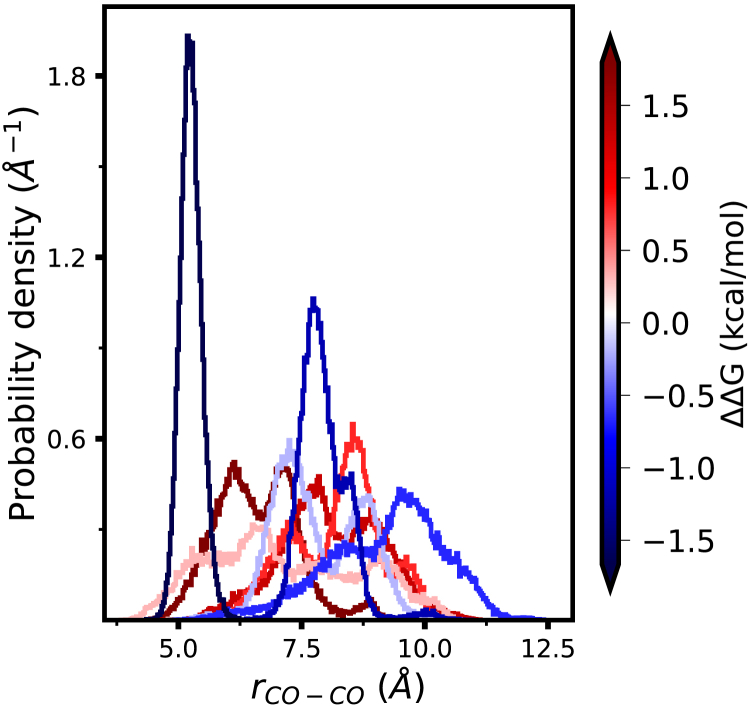
Histogram of the distances between carboxylate carbons of the folded protein L from an MD simulation at *b*_KCl_ = 2 mol·kg^−1^, for the amino acid pairs for which ΔΔG was calculated (Figure 3*Bi*). The color scale shows the value of ΔΔG, with blue used for pairs showing a synergistic interaction and red used for pairs with an interfering interaction. To see this figure in color, go online.

We have observed a substantial synergistic effect in a denatured configuration of the halophilic protein L. This observation contradicts the claim of the lack of necessary preorientation for cation-acidic residue interaction in the unfolded structure. To gain further insight into the connection between the relative orientation of amino acids and their synergistic or repulsive interactions, we simulated the folded halophilic protein L, at *b*_KCl_ = 2 mol·kg^−1^, to obtain a long, continuous trajectory (see supporting material, section 2.2 for simulation details). We analyzed this trajectory to determine whether characteristic configurations of acidic amino acids differ between pairs of amino acids showing synergistic versus interfering effects. Fig. 7 compares the histograms of distances between the carboxylate carbons of the amino acid pairs for which ΔΔG was calculated (Fig. 3 *Bi*). The distribution of distances differs substantially between pairs, and a correlation between the characteristics of the distribution and the magnitude or sign of ΔΔG cannot be discerned. The distribution of distances for pairs of amino acids showing synergistic interactions (in *blue*) can be narrow and associated with short distances (5–6 Å) corresponding to contact ion pair configurations, or can be quite broad and include distances that correspond to solvent-shared ion pair configurations. The distributions of distances for pairs of amino acids showing interfering interactions (in *red*) are equally broad and span the same distances. These results do not support the claim that the synergistic effect, when it exists, arises from amino acids with particular and well-defined relative orientations.

### Enthalpic/entropic origins of the synergistic effect

**Figure 8:**
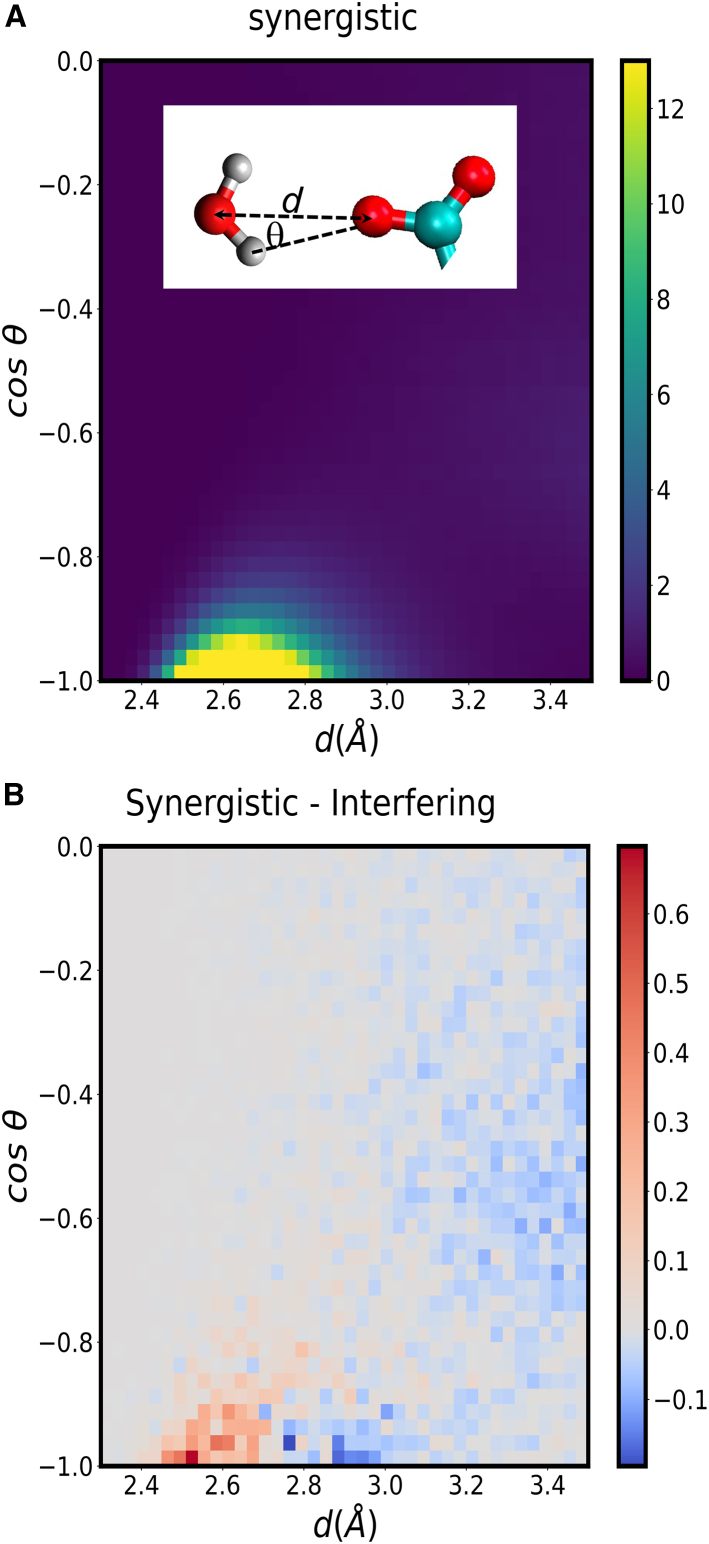
Geometric features of hydrogen bonds donated by water molecules to the carboxylate groups of acidic amino acids in protein L. (*A*) Histogram, *p*_syn_(*d*, cosθ), of the distance and angle characterizing hydrogen bonds accepted by the residues of synergistic pairs. The inset shows a water molecule near a carboxylate group, and illustrates the distance and angle used for the histograms. (*B*) Difference, *p*_syn_(*d*, cosθ) − *p*_int_(*d*, cosθ), between the histograms characterizing hydrogen bonds accepted by residues of synergistic and interfering pairs. To see this figure in color, go online.

To assess whether the synergistic effect observed for some of the protein sites could originate from stronger hydrogen bonds between water and the carboxylate groups of the acidic amino acids in those cases, we quantified the geometry of those hydrogen bonds separately for the synergistic and the interfering pairs of amino acids of protein L from the same simulation on which Fig. 7 is based. Fig. 8 *A* shows the normalized histogram (*p*_syn_(*d*, cosθ)) of the distance (*d*) between the water and the carboxylate oxygens versus the cosine of the angle θ for water molecules near carboxylate groups of the amino acid pairs showing a synergistic effect. The hydrogen bond strength increases for shorter distances and for cosine values close to −1, i.e., for θ near $180^{\circ }$. Water preferentially donates strong hydrogen bonds to the carboxylate groups, as indicated by the short values of *d* and by cosine values near −1. These results are consistent with previous work, which indicates that carboxylates accept strong hydrogen bonds from water (47). In Fig. 8 *B* we show the difference between the histograms obtained for the interfering and for the synergistic residues (*p*_syn_(*d*, cosθ) − *p*_int_(*d*, cosθ)). This difference shows that the synergistic sites accept stronger hydrogen bonds and accept fewer weaker hydrogen bonds than the interfering sites. This result confirms that the synergistic effect has an enthalpic contribution arising from solute-water interactions.

We found no mechanistic correlation between the radial distribution function of the cation and the presence or absence of synergistic interactions. Further assessing the contribution of the cation toward the difference in hydrogen bond strength between water and the carboxylate groups, and assessing whether entropy also contributes to the synergistic effect, will require the quantification of local entropy and enthalpy at the interfering and synergistic protein sites. At present, algorithms to quantify local enthalpic and entropic contributions to solvation free energies (48,49) can only be applied to solutes in pure water, so these aspects cannot be investigated in our system.

## Conclusions

We used molecular simulations to investigate whether synergistic—rather than the expected interfering—interactions between neighboring acidic amino acids exist and contribute to the stabilization of folded halophilic proteins, as originally proposed by Zaccai et al. (24) and supported by NMR studies of halophilic proteins (44). Our results partially support this hypothesis, clarify its mechanism, and point to further studies necessary to fully understand the origin of synergistic interactions.

Quantitative investigations of this hypothesis have been hampered by the lack of an operational definition of synergistic interactions. Our first step was thus to address this difficulty: we quantify the interactions between neighboring amino acids as a difference, ΔΔG, of two mutation free energies, which can be calculated with standard molecular dynamics simulation packages.

Our ΔΔG calculations show that pairs of neighboring acidic amino acids at the surface of proteins in multimolar KCl solutions may indeed interact synergistically. Synergistic interactions are frequent at multimolar KCl concentration but very infrequent at low KCl concentration, suggesting that they have an electrostatic origin. Decomposing ΔΔG into the decharging, vdW, and charging components confirms that synergistic interactions are associated with the decharging step, i.e., with solvent-mediated electrostatic interactions between two acidic amino acids. Hydrogen bond analysis confirms that acidic amino acids with synergistic interactions accept stronger hydrogen bonds from water more often than those with interfering interactions. Further work is necessary to investigate whether synergistic interactions have also an entropic contribution. We find that synergistic interactions do not occur in minimal systems mimicking amino acid side chains, indicating that the protein environment influences the solvent- and ion-mediated interactions between acidic amino acids and contributes to the occurrence of synergistic interactions between them. However, obvious correlations between the secondary structural motif to which the amino acids belong or their spatially close neighboring amino acids and their synergistic/interfering interactions do not exist. Further work—e.g., investigating whether the protein environment creates electric fields of favorable strength and direction at synergistic but not interfering sites—is necessary to fully clarify the mechanism by which the protein environment allows synergistic interactions to occur in some sites.

Our results support the existence of synergistic interactions between acidic amino acids proposed by Zaccai et al. (24), but do not support their proposed mechanism that synergistic interactions are enabled by specific amino acid configurations exclusive to folded halophilic proteins. Instead, the simulations show that synergistic interactions are not associated with specific or rigid amino acid configurations, and exist also in unfolded conformations. According to Zaccai et al., specific amino acid configurations would lead to unusually slow dynamics of the water of hydration because the anchoring of the acidic residues in the protein would limit the number of available configurations and would slow down the hydrogen bond dynamics. Our previous simulations do not support that scenario: we found that the translational dynamics of water of hydration of halophilic proteins is very similar to that of mesophilic ones (20).

Synergistic interactions should nevertheless stabilize folded relative to unfolded configurations, as initially proposed, because the ensemble of unfolded configurations has many more configurations than the folded one. The configurations for which synergistic interactions occur (where the acidic amino acids are spatially close) are only a small subset of the total unfolded ensemble, whereas they dominate the ensemble of folded configurations. Therefore, the stabilizing contribution of synergistic interaction to the unfolded ensemble should be smaller than for the folded one. We emphasize that it remains unclear whether synergistic interactions are the main mechanism by which acidic amino acids stabilize the folded structure of halophilic proteins at high salt concentration. It is also possible that nonsynergistic interactions involving acidic amino acids, solvent, and ions lead to different solvation of folded versus unfolded configurations, and that these differences stabilize the folded state (50). Future studies in our group will investigate this possibility.

## Author contributions

A.V.V. designed and guided the research. H.G.D. performed the research. Both authors wrote the manuscript.

## Supporting material

Document S1. Figures S1–S12 and Tables S1–S7

Document S2. Article plus supporting material

## Footnotes

Supporting material can be found online at https://doi.org/10.1016/j.bpj.2023.05.011.
