# Supplementary material for: Prevalence and mechanism of synergistic carboxylate-cation-water interactions in halophilic proteins
Source: Biophys J. 2023 May 12;122(12):2577–89. doi: 10.1016/j.bpj.2023.05.011 (PMC10323026; doi:10.1016/j.bpj.2023.05.011)
Supplement: Document S1. Figures S1–S12 and Tables S1–S7 [file mmc1.pdf]

Biophysical Journal, Volume *122*

Supplemental information

Prevalence and mechanism of synergistic carboxylate-cation-water interactions in halophilic proteins

Hosein Geraili Daronkola and Ana Vila Verde

S1 Molecular formula of amino acids

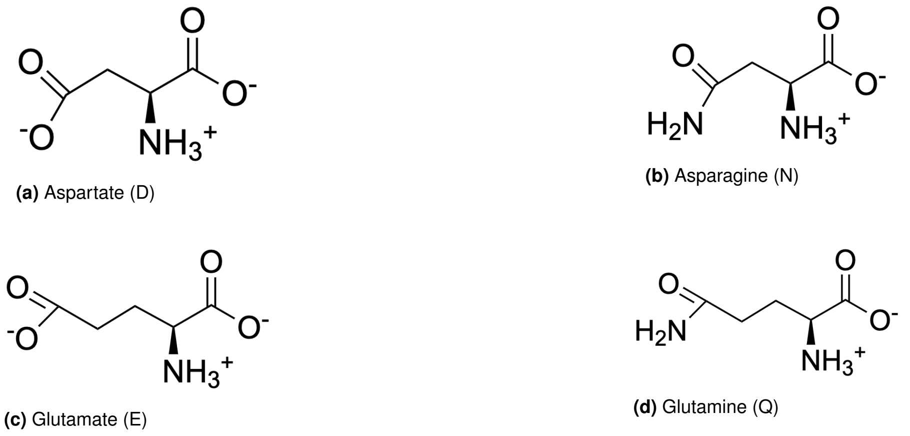

(a) Aspartate (D) **(b)** Asparagine (N) **(c)** Glutamate (E) **(d)** Glutamine (Q)

Fig. S1 Molecular formula of each amino acid in its zwitterionic form.

S2 Simulation details

S2.1 Free energy calculations

The free energy values reported in the main text were obtained from free energy calculations using optimized simulation parameters described in this section; section S3 describes how the optimized parameters were determined.

For step (2) in Scheme 1 in the main text, 34 λ values of 0.00 (X_a^0), 0.02, 0.04, 0.06, 0.08, 0.10, 0.12, 0.14, 0.16, 0.18, 0.20, 0.22, 0.24, 0.26, 0.28, 0.30, 0.35, 0.40, 0.45, 0.50, 0.55, 0.60, 0.65, 0.70, 0.75, 0.80, 0.85, 0.88, 0.90, 0.92, 0.94, 0.96, 0.98, 1.00 (Y_a^0) were used. The value returned by this calculation is ΔG_{vdW}. For practical reasons, in the case of steps (1) and (3), the thermodynamic steps actually calculated were the reverse of those shown in Scheme 1. For step (1), 37 λ values of 0.00 (X_a^0), 0.05, 0.10, 0.15, 0.20, 0.25, 0.30, 0.35, 0.40, 0.45, 0.50, 0.55, 0.60, 0.61, 0.62, 0.63, 0.64, 0.65, 0.66, 0.67, 0.68, 0.69, 0.70, 0.71, 0.72, 0.73, 0.74, 0.75, 0.76, 0.77, 0.78, 0.79, 0.80, 0.85, 0.90, 0.95, 1.00 (X_a) were used. The value returned by this calculation was $-\Delta G_{\text{decharge}}$. For step (3), 24 λ values of 0.00 (Y_a), 0.02, 0.04, 0.06, 0.08, 0.10, 0.15, 0.20, 0.25, 0.30, 0.35, 0.40, 0.45, 0.50, 0.55, 0.60, 0.65, 0.70, 0.75, 0.80, 0.85, 0.90, 0.95, 1.00 (Y_a^0) were used. The value returned by this calculation is $-\Delta G_{\text{charge}}$.

For each value of λ, the starting configuration was minimized using a steepest-descent algorithm for 10000 steps with a cutoff distance of 12 Å for both Lennard-Jones and electrostatic interactions. The simulation box was equilibrated in the NpT ensemble for 1 ns. Using the Berendsen barostat[1] with a relaxation time of 2.0 ps, the average system pressure was kept at 1.0 bar. Also, in this 1 ns equilibration step, using a Langevin thermostat with a coupling constant of 5.0 ps^{-1} the simulation box was heated for 250 ps between 0 and 298 K, after which the average temperature of the system was kept at 298 K. Lennard-Jones interactions and direct electrostatic interactions were calculated up to a cutoff of distance 10 Å. Beyond this cutoff, electrostatic interactions were calculated with the Particle Mesh Ewald (PME) algorithm[2] with a grid spacing of 1 Å, and fourth order interpolation. Long-range dispersion corrections were applied to both the energy and pressure. The protein backbone atoms (N, C_α, C, O) were restrained to their initial positions using a harmonic restraint with a force constant of 35 kcal·mol^{-1}·Å^{-2}. The value of *skinnb* was increased to 5 Å. The production phase of the simulation was performed in the NVT ensemble and lasted 10 ns, using the Langevin thermostat with a coupling constant of 5.0 ps^{-1} to keep the average temperature at 298 K. The cutoff value for the calculation of Lennard-Jones and direct electrostatic interactions was increased to 12 Å.

Soft-core potentials were used for the residues participating in the mutation, taking the AMBER default

parameter values of α=0.5 and β=12.0 Å^2 which control the softness of this potential. The SHAKE algorithm was used to constrain the length of bonds involving hydrogen atoms, except those in the two residues involved in the mutation. We used a time step of 1 fs in all the simulations.

S2.1.1 Evaluating the quality of the free energy calculations

In Figure S2 we show the result of the integrations for the mutation (D81)...D83N on the surface of 1DOI protein at b_{KCl} = 2 mol·kg^{-1} concentration. This figure shows the vdW (Figure S2a), charge (Figure S2b), and decharge (Figure S2c) curves of integration of $\langle \partial U(\lambda)/\partial\lambda \rangle_{\lambda_i}$ versus λ_i values. Usually, the integration for the charge step is smooth. In contrast, there is a sharp change around $0.65 < \lambda < 0.78$ in the decharge step, so we used a $\Delta\lambda$ of 0.01 in this region to be able to capture the change more accurately. As for the vdW step, the integration is not always very smooth. We did not increase the number of λ values for this step because the results show (see Tables S4,S6,S7 and S5) that it contributes minimally to the total free energy change of the mutation.

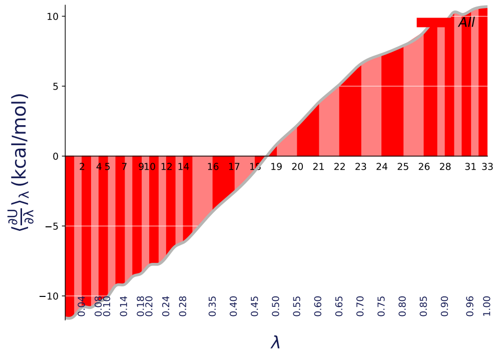

(a) vdW

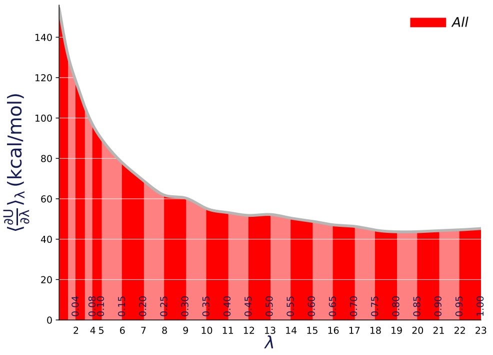

(b) charge

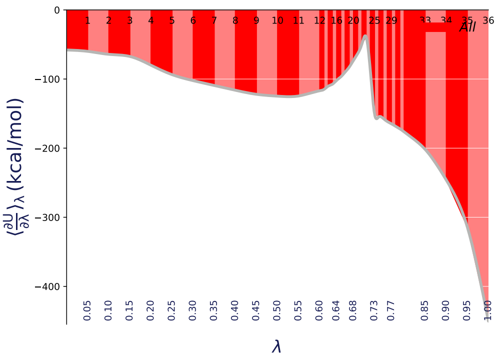

(c) decharge

Fig. S2 Plot of $\left\langle \frac{\partial U}{\partial \lambda} \right\rangle_\lambda$ vs. λ values for the thermodynamic integration corresponding to the (D81)...D83N mutation at b_{KCl} = 2 mol·kg^{-1} of protein 1DOI. The light and dark red filled areas indicate free energy estimates from the TI-1 method; the silver curve indicates interpolation via the TI-3 method. The agreement between the two interpolation schemes suggests that the interpolation successfully captures the free energy change between two neighboring λ, as well as over the whole range. The subfigures correspond to the three legs of the thermodynamic cycle shown in Scheme 1 in the main text, with (a) vdW, (b) charge, and (c) decharge. Free energy values calculated with the TI-3 method: $-\Delta G_{\mathrm{charge}} = 59.50194$ kcal·mol^{-1}; $-\Delta G_{\mathrm{decharge}} = -133.7835$ kcal·mol^{-1}; $\Delta G_{\mathrm{vdW}} = 0.23334$ kcal·mol^{-1}, resulting in a total free energy change of 74.5149 kcal·mol^{-1}.

S2.1.2 Calculating the standard error of the free energies

We made the assumption that the statistical error associated with the mutation free energy values is the same for every protein site we investigated. We estimated this error by calculating the Standard Error of the Mean (SEM) for one case only: the mutation (E26)...D29N of protein 1DOI at b_{KCl} = 2 mol·kg^{-1} (see also Table S4). We performed 5 independent simulations using the optimized simulation protocol described in Section S2.1. The resulting free energy values are shown in Table S1.

Table S1 Free energy values and their SEM (kcal·mol^{-1}) associated with the (E26)...D29N of protein 1DOI at b_{KCl} = 2 mol·kg^{-1}, from 5 independent simulations. These SEM values were taken as estimates of the statistical uncertainty of all free energy values reported in the main text.

Simulation	1	2	3	4	5	SEM
$-\Delta G_{\mathrm{charge}}$	56.26149	56.54705	56.56241	56.45222	56.99566	0.12
$-\Delta G_{\mathrm{decharge}}$	-132.62702	-131.24322	-133.03272	-133.62545	-134.52607	0.55
ΔG_{vdW}	-0.64023	-0.61521	-0.73233	-0.61889	-0.60338	0.02
ΔG_{XaY} (total)	75.7253	74.08096	75.73798	76.55434	76.92703	0.49

The SEM associated with each step (charge, decharge and vdW) was calculated using the following expressions:

$$\sigma = \sqrt{\frac{\sum_{i=1}^{N}(x_i - \bar{x})^2}{N-1}} \tag{1}$$

$$\mathrm{SEM} = \frac{\sigma}{\sqrt{N}} \tag{2}$$

where $N = 5$ is the number of independent simulation runs for each simulation step (charge, decharge and vdW), x_i is the free energy value of each simulation step, $\bar{x}$ is the mean over the N values, and σ is the standard deviation.

S2.2 MD of folded protein L.

The folded halophilic protein L. was simulated at b_{KCl}= 2 mol·kg^{-1} with the same parameters as for the mutation free energy simulations, to obtain a continuous trajectory with frequently saved configurations for detailed structural analysis. This simulation used the same starting configuration as well as the same minimization and equilibration protocols as for the mutation free energy calculations of this protein. In the production phase, the system was simulated in the NVT ensemble for 400 ns. The final trajectory contains 4×10^4 configurations saved every 10 ps.

S2.3 Simulation details for the potential of mean force calculations

All simulations for the PMF calculations were done using the GROMACS 2020 simulation package.[3,4] The simulation boxes were prepared by placing the side chains in appropriate positions near the center of a cubic box with an edge length $L \approx 6$ nm and then adding the appropriate number of TIP3P water molecules and potassium and chloride ions to create an aqueous solution of the desired potassium chloride molality. In all steps of the simulations, electrostatic interactions were calculated using direct summation up to 1.2 nm, and using the particle mesh Ewald[2] (PME) scheme with a grid spacing of 0.12 nm beyond this cutoff distance. Lennard-Jones interactions were smoothly shifted to zero between 1.0 nm and 1.2 nm using the switch function available in GROMACS. Long-range dispersion corrections were applied to both the energy and pressure. A leap-frog stochastic (SD) integrator[5] was used to integrate the equations of motion in all simulations. All bonds with H-atoms were restrained using the LINCS algorithm[6] in all simulation steps (except the minimization step with the l-bfgs method), which enables integration using a 2 fs time step.

The initial configurations were equilibrated as follows: i) Two initial minimization steps with the

steepest-descent and l-bfgs algorithms. The latter is a quasi-Newtonian algorithm for energy minimization, which converges faster than the Conjugate-Gradient algorithm. ii) A 500 ps heating equilibration simulation in the canonical ensemble using the Langevin thermostat with a coupling constant of 1.0 ps and using a target temperature of 298 K. iii) Another 6 ns simulation in the isothermal-isobaric ensemble to equilibrate the system density at the pressure of 1 bar, using the Berendsen barostate[1] with a relaxation time of 1.0 ps. In all of these equilibration steps, the C_αs were restrained to their initial position using a harmonic restraint with a force constant of 10000 kJ·mol^{-1}·nm^{-2}.

To generate starting configurations for the umbrella simulations, we performed pulling simulations in the isothermal-isobaric ensemble. The pressure was controlled using the Parrinello-Rahman barostat[7,8] with a relaxation time of 1.0 ps and a target pressure of 1 bar. The temperature was controlled separately for the water and the remaining species using two Nose-Hoover thermostats, each with a coupling constant of 1.0 ps and a target temperature of 298 K. The C_α of one side chain was restrained to its initial position, in the proximity of the box center, using a harmonic restraint with a force constant of 10000 kJ·mol^{-1}·nm^{-2}. For the 2-body system, the second residue was pulled along the 3-D vector associated with the distance ξ. For the 3-body system, the third residue was pulled along the 3-D vectors associated with the distances ξ_1 and ξ_2. In all cases the pulling simulations had a duration of 1.5 ns, the pull rate was 1 nm·ns^{-1}, the distance restraints were enforced with a harmonic potential with a force constant of 10000 kJ·mol^{-1}·nm^{-2}, and 6 angular restraints and 3 dihedral harmonic restraints (described in the main text) were applied to ensure that the 3 side chains were approximately parallel to each other and were perpendicular to the plane defined by the three C_α. The force constant for the angular restraints was 2000 kJ·mol^{-1}·rad^{-2} and for the dihedral restraints were 2000 kJ·mol^{-1}·rad^{-2}. The pulling simulations were post-processed using in-house python scripts to extract individual configurations where the reaction coordinates ξ, ξ_1 and ξ_2 vary between $\approx$ 2.5 Å and 15 Å in 0.5 Å steps. These configurations were used as the starting configurations of the umbrella simulations.

Each umbrella simulation lasted 60 ns. The distance, angle and dihedral reaction coordinates were restrained to their values in the starting configuration used for each umbrella simulation. The force constants for the distance restraints were 5000 kJ·mol^{-1}·nm^{-2}; for the angular and dihedral restraints, force constants of 500 kJ·mol^{-1}·rad^{-2} were used.

S3 Identifying the best computational scheme to calculate free energies of mutation

Optimized simulation protocols for mutation free energy calculations of the type of system investigated here – highly charged proteins at high salt concentration in explicit water, with mutations leading to a change in the total charge of the system, and where the accuracy and precision of the calculated values are critical for the desired study – have not been reported. To address this issue, we investigated how the number of intermediate states, conformational sampling adequacy, finite-size effects in free energy calculation and post-processing free energy calculation methods impact the results. In the computational test studies that follow, the simulation settings were those used in the final simulations unless explicitly noted otherwise.

The calculations of the free energy associated with an amino acid mutation are done using the 3-step protocol described in Scheme 1 in the main text: the charge, decharge, and vdW steps are performed separately. Mutation free energies could, in principle, have been calculated using a one-step protocol corresponding to the direct mutation of residue, ΔG_{XaY}, in Scheme 1, by applying softcore potentials to the vdW and electrostatic interactions. We opted not to do so because Garton et al.[9] have concluded in their study that, for mutations leading to a change in the electric charge, the 3-step protocol yields more accurate free energy values than the 1-step protocol. They have also indicated that if a change in charge with the mutation does not occur, the 3-step protocol should always be avoided.

S3.1 Finite-size effects

Mutation free energies involving charge changes and in explicit solvent might suffer from significant finite-size effects[10]. Finite-size effects occur when the result of a simulation in a periodic boundary box and that done in an infinite bulk medium differ. They arise primarily because of the treatment of long-range electrostatic interactions in simulations. When performing simulations in periodic boxes, finite-size effects have two main sources: firstly, the extra electrostatic interaction between the solute in the computational reference box, its periodic replicas, and the homogeneous background charge density; and secondly, the undersolvation of the solute in the reference box because the solvent in the periodic boxes is perturbed by the image of the solute in the respective box, and is therefore unavailable for the solute in the main box[10]. Analytical and numerical schemes have been proposed[10–13] to correct these issues, but not all commonly used simulation packages have implemented them. To check how finite size effects impact the results of free energy calculations in AMBER[14] when using Particle Mesh Ewald (PME)[15] to calculate long-range electrostatics, we calculated the solvation free energy (ΔG_{solv}) of one potassium ion in a box of TIP3P water, for different box sizes. The production simulation was performed after minimization and heating; it used a timestep of 2 fs and lasted 10 ns. The SHAKE algorithm was used for every bond involving hydrogen. The average temperature was kept at 298 K using the Langevin thermostat with a coupling constant of 2 ps^{-1}, and the average pressure was kept at 1 bar using the Berendsen barostat with a coupling constant of 2 ps. A distance cutoff of 8 Å was used for the van der Waals interactions and for the direct calculation of electrostatics interactions. The potassium ion was transferred to the gas phase using a one-step protocol. In total 51 λ values of 0.00 (ion fully coupled to the solution), 0.02, 0.04, 0.06, 0.08, 0.10, 0.12, 0.14, 0.16, 0.18, 0.20, 0.22, 0.24, 0.26, 0.28, 0.30, 0.32, 0.34, 0.36, 0.38, 0.40, 0.42, 0.44, 0.46, 0.48, 0.50, 0.52, 0.54, 0.56, 0.58, 0.60, 0.62, 0.64, 0.66, 0.68, 0.70, 0.72, 0.74, 0.76, 0.78, 0.80, 0.82, 0.84, 0.86, 0.88, 0.90, 0.92, 0.94, 0.96, 0.98, 1.00 (ion fully decoupled from solution (gas phase)) was used.

The $\langle \partial U/\partial \lambda \rangle_\lambda$ vs. λ plots for all the four different box sizes as well as the values of $-\Delta G_{\mathrm{solv}}$ are shown in Figure S3. We find that the solvation free energy values vary between -93.8 and -94.6 kcal·mol^{-1} for boxes with edge-length varying between 45 and 70 Å. Moreover, they do not vary monotonically with edge-length. The results confirm that the Amber 18 code has the appropriate correction schemes in place and thus that our calculated mutation free energy values have minimal influence from finite-size effects.

We further note that, because we used the same box size for each protein and each salt concentration

in all the mutation free energy calculations reported in the main text, differences between the reported values cannot be due to finite-size effects.

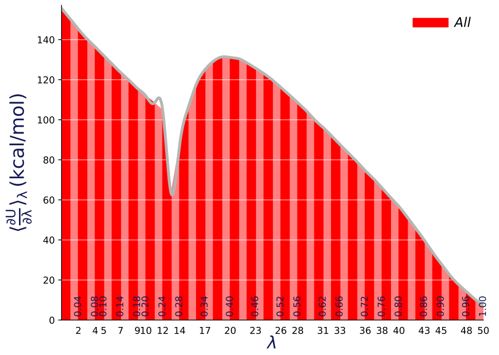

(a) Box-edge length ≈ 70 Å. Total free energy 93.78527 kcal·mol^{-1}.

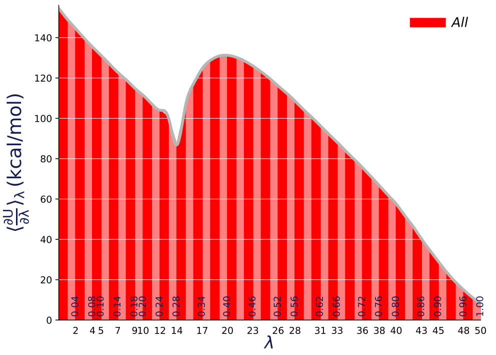

(b) Box-edge length ≈ 60 Å. Total free energy 94.56233 kcal·mol^{-1}.

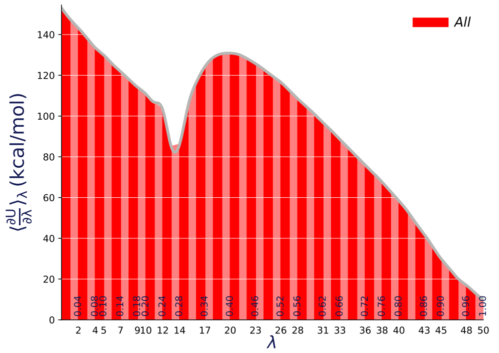

(c) Box-edge length ≈ 50 Å. Total free energy 94.30012 kcal·mol^{-1}.

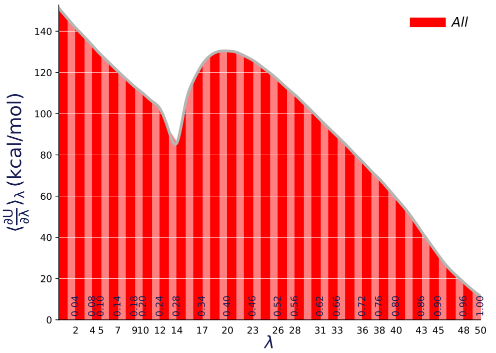

(d) Box-edge length ≈ 45 Å. Total free energy 94.50033 kcal·mol^{-1}.

Fig. S3 Plot of $\left\langle \frac{\partial U}{\partial \lambda} \right\rangle_\lambda$ vs. λ values for the integration. This figure shows the mutation corresponding to annihilating a potassium ion. The details of the simulation are explained in Subsection S3.1.

S3.2 Simulation time, number of intermediate states and post-processing method

To assess whether 10 ns of simulation time for each λ enabled sufficient sampling, we compared the final free energy associated with the mutation (D81)...D83N of protein ferredoxin (pdb ID: 1DOI) at b_{KCl} = 2 mol·kg^{-1} (compare also with Figure S2) against that obtained from simulations that used 30 ns of simulation time for each λ. All the simulation details are the same as the final free energy simulations, except that the SHAKE algorithm was not used for the heating and production runs. Figures S4 and S5 show the $\langle \partial U/\partial\lambda \rangle_\lambda$ vs. λ for the two cases. The results confirm that the shape of the curves is very similar in both cases, indicating that 10 ns is sufficient simulation time to sample the system at each value of λ.

To assess whether a total number of 95 λ (34 for the vdW step, 37 for decharge, and 24 for charge) enabled us to capture the shape of the $\langle \partial U/\partial\lambda \rangle_\lambda$ vs. λ curves with sufficient definition, we performed another test simulation where the total number of λ was increased dramatically to 177: 59 λ values of 0.00, 0.01, 0.02, 0.03, 0.04, 0.05, 0.06, 0.08, 0.12, 0.14, 0.16, 0.20, 0.24, 0.28, 0.32, 0.36, 0.40, 0.44, 0.48, 0.52, 0.56, 0.58, 0.60, 0.62, 0.64, 0.65, 0.66, 0.67, 0.68, 0.70, 0.71, 0.72, 0.73, 0.74, 0.75, 0.76, 0.77, 0.78, 0.79, 0.80, 0.81, 0.82, 0.83, 0.84, 0.85, 0.86, 0.87, 0.88, 0.89, 0.90, 0.91, 0.93, 0.94, 0.95, 0.96, 0.97, 0.98, 0.99, 1.00 for each of the vdW, decharge and charge steps. The simulation details were precisely the same as those of the previous simulations described in this subsection, with a 10 ns of total production simulation time. Figure S6 shows the integration curves corresponding to the different steps of this simulation. The curves have very similar shapes in both cases, confirming that 95 values of λ are sufficient to accurately estimate the free energies.

Table S2 quantitatively compares the results of these simulations by processing the simulation data using an integration-based method (TI-3) and using a perturbation-based method (BAR). Integration methods are sensitive to the smoothness of the $\langle \partial U/\partial\lambda \rangle_\lambda$ curve; in contrast, perturbation-based methods are sensitive to the extent of phase space overlap between adjacent λ windows. Agreement between the free energy values calculated with both methods suggests that the simulations adequately sampled the system and thus that the results are reliable. The values obtained with TI-3 and with BAR are almost identical for every case. Increasing the simulation time per λ alters the total free energy values by less than 0.1 kcal·mol^{-1}, confirming that there is no advantage in increasing the simulation time beyond 10 ns. Increasing the number of λ values altered the total free energy by 0.65 kcal·mol^{-1}, i.e., only slightly higher than the estimated SEM (section S2.1.2) of $\pm$0.49 kcal·mol^{-1} associated with the free energy values.

Table S2 Free energy values (kcal·mol^{-1}) calculated from different perturbation-based (BAR), and integration-based (TI-3) methods, for the indicated simulation conditions (number of λ values and simulation time per λ value).

		decharge		charge		vdW		total	
	Simulation time (ns)	TI-3	BAR	TI-3	BAR	TI-3	BAR	TI-3	BAR
**95λ**	10 (fig. S4)	-135.79401	-135.49497	60.04460	59.89161	0.15400	0.19775	75.90341	75.80111
	30 (fig. S5)	-135.33353	-135.38722	59.66333	59.65385	0.13958	0.17432	75.80978	75.90769
**177λ**	10 (fig. S6)	-134.75056	-134.36508	59.76601	59.73252	0.27122	0.25510	75.25577	74.88766

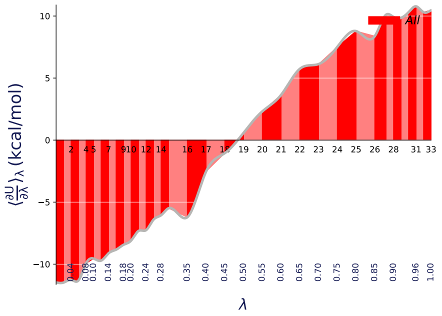

(a) vdW

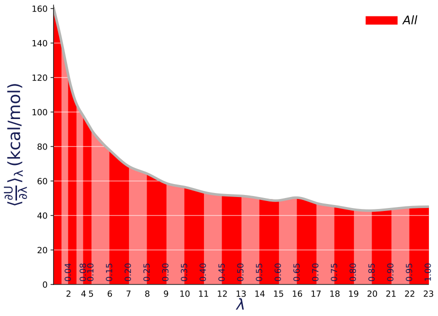

(b) charge

images/dhdl_TI_decharge10ns.pdf

(c) decharge

Fig. S4 Plot of $\left\langle \frac{\partial U}{\partial \lambda} \right\rangle_\lambda$ vs. λ values for the (D81)...D83N mutation of protein 1DOI at b_{KCl} = 2 mol·kg^{-1}, with 10 ns of simulation time per λ. The details of the simulation are explained in Subsection S3.2.

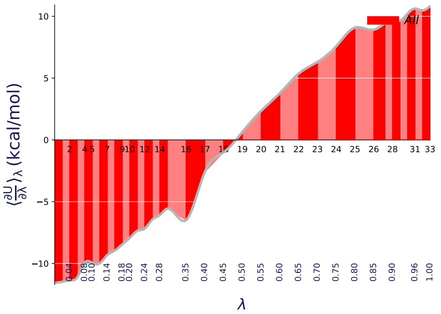

(a) vdW

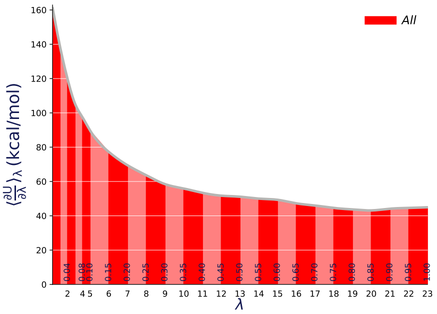

(b) charge

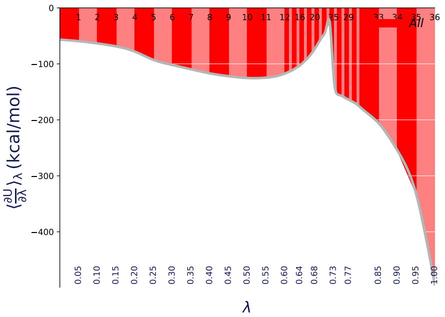

(c) decharge

Fig. S5 Plot of $\left\langle \frac{\partial U}{\partial \lambda} \right\rangle_\lambda$ vs. λ values for the (D81)…D83N mutation of protein 1DOI at b_{KCl} = 2 mol·kg^{-1}, with 30 ns of simulation time per λ. The details of the simulation are explained in Subsection S3.2.

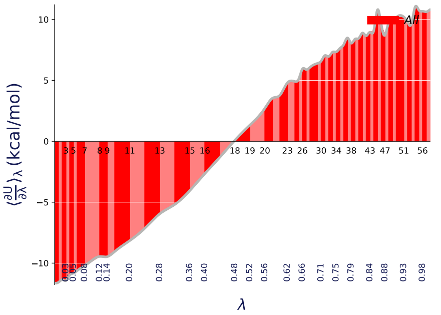

(a) vdW

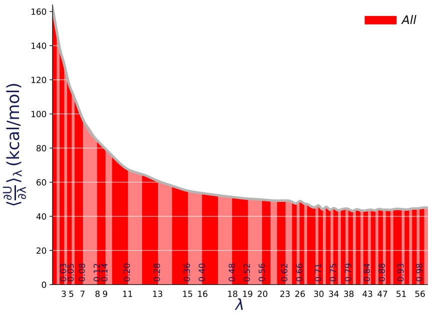

(b) charge

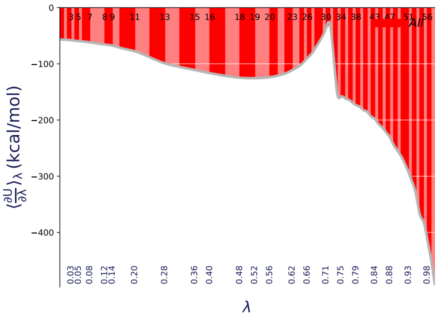

(c) decharge

Fig. S6 Plot of $\left\langle \frac{\partial U}{\partial \lambda} \right\rangle_\lambda$ vs. λ values for the (D81)…D83N mutation of protein 1DOI at b_{KCl} = 2 mol·kg^{-1}, with a total of 177 λ. The details of the simulation are explained in Subsection S3.2.

S3.3 Restraining the backbone of proteins

We calculated the free energy associated with the D34N mutation of protein 1DOI at b_{KCl} = 2 mol·kg^{-1}, with and without backbone restraints. For each value of λ the system was heated from 0 to 298 K over 300 ps with a timestep of 1 fs, using a Langevin thermostat with a coupling constant 1 ps^{-1}. A harmonic restraint with a force constant of 10 kcal·mol^{-1}·Å^{-2} on all heavy atoms of the protein was used during the heating stage; the SHAKE algorithm was not applied. After the target temperature 298 K was reached, the average temperature was kept constant at that value. The production simulation lasted 30 ns. A timestep of 2 fs was used because the SHAKE algorithm was used for all the bonds connected to the hydrogen atoms, including the mutating residues. The Langevin thermostat was used to keep the average temperature at 298 K using a coupling constant of 2 ps^{-1}, and the Berendsen barostat with a coupling constant of 2 ps kept the average pressure at 1 bar. The distance cutoff for the calculation of Lennard-Jones interactions and for the direct calculation of electrostatic interactions was 12 Å. For one simulation, harmonic restraints with bond constants of 50 kcal·mol^{-1}·Å^{2} were applied to the backbone atoms N, C_α, C, and O; for the other, the restraints were turned off. In total 26 λ values of 0.00 (X), 0.04, 0.08, 0.12, 0.16, 0.20, 0.24, 0.28, 0.32, 0.36, 0.40, 0.44, 0.48, 0.52, 0.56, 0.60, 0.64, 0.68, 0.72, 0.76, 0.80, 0.84, 0.88, 0.92, 0.96, 1.00 (Y) corresponding to direct mutation in one step instead of three, in the scheme 1 were used.

The two cases are compared in Table S3 and in Figures S7a and S7b. Restraining the backbone resulted in a $\langle \partial U/\partial\lambda \rangle_\lambda$ vs. λ curve that is smoother and easier to integrate; it also resulted in free energy estimates that are much more similar between TI-3 and BAR than when restraints were not applied. The absence of restraints enables small conformational changes unrelated to the mutation but which affect the mutation free energy by a few kcal·mol^{-1}. Producing free energy values that average over all the small conformational changes is computationally prohibitive. Because this variation would make it impossible to make the detailed comparisons of free energy values that are at the core of this study, the protein backbone was restrained in the final free energy calculations.

Table S3 Free energy values (kcal·mol^{-1}) associated with the D34N mutation of the protein 1DOI at b_{KCl} = 2 mol·kg^{-1}, calculated from different perturbation-based (BAR) and integration-based (TI-3), from simulations where the backbone was restrained or free.

Backbone	TI-3	BAR
Restrained (fig. S7a)	72.75423	73.61249
Free (fig. S7b)	76.03225	73.29420

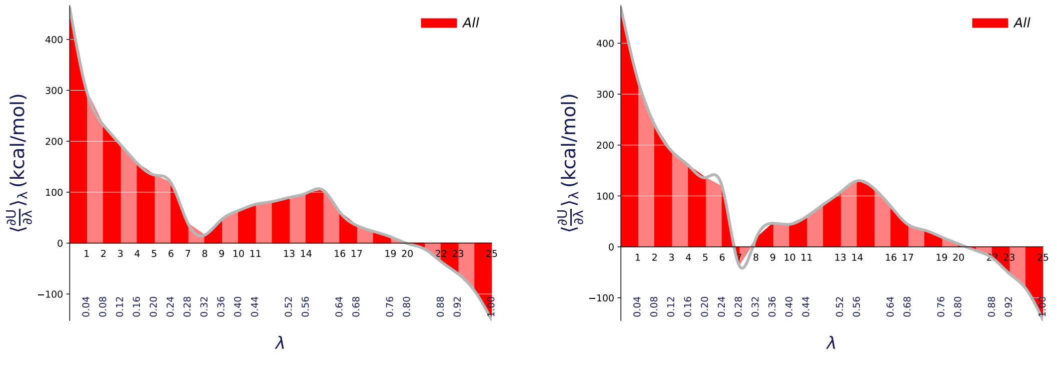

(a) Backbone restrained **(b)** Backbone free

Fig. S7 Plot of $\left\langle \frac{\partial U}{\partial \lambda} \right\rangle_\lambda$ vs. λ values for the D34N mutation of protein 1DOI at b_{KCl} = 2 mol·kg^{-1}. The details of the simulation are explained in Subsection S3.3.

S4 Free energy of mutations in protein L., ferredoxin, and dihydrofolate reductase

Table S4 Free energies (kcal·mol^{-1}) of mutation for the halophilic protein ferredoxin. The standard error of the mean (SEM) calculated as described in S2.1.2 is reported for each value. $\Delta G_{\mathrm{X}a\mathrm{Y}} = \Delta G_{\mathrm{decharge}} + \Delta G_{\mathrm{vdW}} + \Delta G_{\mathrm{charge}}$; see Scheme 1.

	b_{KCl} = 2 mol·kg^{-1}					b_{KCl} = 0.15 mol·kg^{-1}				
**mutation**	-$\Delta G_{\mathrm{charge}}$ (±0.1)	-$\Delta G_{\mathrm{decharge}}$ (±0.6)	ΔG_{vdW} (±0.02)	$\Delta G_{\mathrm{X}a\mathrm{Y}}$ [a] (±0.5)	distance [b]	-$\Delta G_{\mathrm{charge}}$ (±0.1)	-$\Delta G_{\mathrm{decharge}}$ (±0.6)	ΔG_{vdW} (±0.04)	$\Delta G_{\mathrm{X}a\mathrm{Y}}$ (±0.5)	distance (Å)
(D12)...D13N	60.76736	-137.79309	-0.55357	76.47216	5.63	63.24650	-140.28636	-0.56827	76.47159	5.96
(N12)...D13N	59.74974	-138.17301	-0.50017	77.9231	5.53	62.57828	-141.54472	-0.60791	78.35853	5.94
(E26)...D29N	56.26149	-132.62702	-0.64023	75.7253	6.36	57.82612	-133.13112	-0.09011	75.21489	5.19
(Q26)...D29N	56.11569	-133.70953	-0.60941	76.98443	7.74	56.45076	-132.36193	-0.09076	75.82041	5.41
(D81)...D107N	63.03267	-141.40526	-1.55017	76.82242	4.36	61.32781	-139.49030	-1.58711	76.57538	5.32
(N81)...D107N	62.65884	-141.83911	-1.64680	77.53347	5.58	61.34764	-140.34335	-1.78861	77.2071	5.23
(D83)...D81N	63.83078	-139.56623	0.32738	76.06283	10.97	66.46505	-142.27500	-0.85744	74.95251	6.04
(N83)...D81N	63.70651	-139.70081	0.25179	76.24609	9.86	66.10691	-143.31791	-0.93470	76.2763	4.84
(D109)...E110Q	57.22122	-135.08841	-0.59288	77.27431	5.53	57.41629	-134.64142	-0.33888	76.88625	7.65
(N109)...E110Q	56.89173	-134.29882	-0.75998	76.64711	6.74	57.98058	-136.46016	-0.10906	78.37052	8.50

[a] Combination of all thermodynamic cycles' free energy, and multiplying the values of $-\Delta G_{\mathrm{charge}}$, and $-\Delta G_{\mathrm{decharge}}$ part by a negative to fit the scheme 1 with the associated value of standard error of the mean. [b] The average distance between the terminal carbons of the side-chain of the mutating residue, D or E, and that of the neighboring residue, D, E, N, Q.The average is taken from the $-\Delta G_{\mathrm{vdW}}$ step of the mutation simulation at λ zero.

Table S5 Free energy (kcal·mol^{-1}) of mutation of aspartic acid to asparagine for halophilic protein L. The standard error of the mean (SEM) calculated as described in S2.1.2 is reported for each value.

	b_{KCl} = 2 mol·kg^{-1}					b_{KCl} = 0.15 mol·kg^{-1}				
**mutation**	-$\Delta G_{\mathrm{charge}}$ (±0.1)	-$\Delta G_{\mathrm{decharge}}$ (±0.6)	ΔG_{vdW} (±0.04)	$\Delta G_{\mathrm{X}a\mathrm{Y}}$ (±0.5)	distance	-$\Delta G_{\mathrm{charge}}$ (±0.1)	-$\Delta G_{\mathrm{decharge}}$ (±0.6)	ΔG_{vdW} (±0.04)	$\Delta G_{\mathrm{X}a\mathrm{Y}}$ (±0.5)	distance (Å)
(E2)...E3Q	60.94070	-140.71040	-0.54365	79.22605	9.95	57.32818	-141.04686	-0.46305	83.25563	8.93
(Q2)...E3Q	61.10848	-139.17715	-0.35378	77.71489	9.97	57.41838	-141.39776	-0.36833	83.61105	9.58
(E28)...E32Q	65.31491	-142.93726	-0.33877	77.28358	7.87	66.00519	-141.92388	-0.46811	75.45058	8.07
(Q28)...E32Q	65.48402	-143.10207	-0.20677	77.41128	7.55	65.05565	-143.16430	-0.65581	77.45284	9.12
(E41)...E42Q	64.44933	-142.49745	-0.32551	77.72261	8.85	63.36859	-142.30051	0.02803	78.95995	7.67
(Q41)...E42Q	63.29743	-142.96200	-0.47503	79.18954	8.71	63.41466	-143.84089	-0.30592	80.12031	8.56
(D38)...E41Q	71.77454	-149.83710	-0.93222	77.13034	5.22	70.41324	-148.54546	-0.43475	77.69747	6.62
(N38)...E41Q	70.65305	-147.07521	-0.95782	75.46434	4.73	69.17052	-146.88446	-0.69658	77.01736	5.94
(E42)...D43N	57.20438	-135.56766	-0.67833	77.68495	6.17	57.07769	-134.68398	-0.43039	77.1759	6.28
(Q42)...D43N	56.67696	-134.85054	-0.46103	77.71255	6.00	57.89925	-137.23697	-0.34339	78.99433	5.76
(E23)...E21Q	60.58391	-136.00383	-1.04906	74.37086	8.57					
(Q23)...E21Q	60.04999	-134.23224	-0.54084	73.64141	7.87					
(E61)...D50N	59.29745	-138.22341	-0.93365	77.99231	6.96					
(Q61)...D50N	58.93284	-137.70456	-1.10447	77.66725	6.21					
(E28)...E27Q	66.99108	-142.59274	-0.22245	75.37921	6.30					
(Q28)...E27Q	62.82825	-140.17936	-0.17533	77.17578	7.15					

Table S6 Free energy (kcal·mol^{-1}) of mutation of aspartic acid to asparagine for halophilic protein Dihydrofolate reductase. The standard error of the mean (SEM) calculated as described in S2.1.2 is reported for each value.

$b_{\mathbf{KCl}}$ = 2 mol·kg^{-1}					
**mutation**	-ΔG_{charge} (±0.1)	-$\Delta G_{\text{decharge}}$ (±0.6)	ΔG_{vdW} (±0.04)	$\Delta G_{\text{X}a\text{Y}}$ (±0.5)	distance
(D18)...E20Q	56.77630	-130.71713	-0.52123	73.4196	5.95
(N18)...E20Q	56.41874	-133.81749	-0.75389	76.64486	9.88
(D54)...D55N	56.58246	-136.05554	-0.73275	78.74033	7.23
(N54)...D55N	55.64233	-135.87124	-0.70413	79.52478	6.46
(E133)...D135N	66.45264	-145.07489	-0.34306	78.27919	7.90
(Q133)...D135N	66.52757	-143.00993	-0.31854	76.16382	8.11
(D135)...E138Q	69.22918	-146.38492	-0.30062	76.85512	5.78
(N135)...E138Q	68.29959	-146.02664	-0.27535	77.4517	5.26
(E144)...D146N	57.65902	-131.85082	-0.35306	73.83874	6.11
(Q144)...D146N	57.69196	-133.70504	-0.23425	75.77883	5.53

Table S7 Free energy (kcal·mol^{-1}) of mutation of Aspartic acid to Asparagine for unfolded halophilic protein L. The standard error of the mean (SEM) calculated as described in S2.1.2 is reported for each value.

$b_{\mathbf{KCl}}$ = 2 mol·kg^{-1}					
**mutation**	-ΔG_{charge} (±0.1)	-$\Delta G_{\text{decharge}}$ (±0.6)	ΔG_{vdW} (±0.04)	$\Delta G_{\text{X}a\text{Y}}$ (±0.5)	distance
(E2)...E3Q	61.22625	-140.86904	-0.14001	79.50278	9.38
(Q2)...E3Q	62.13673	-141.91212	-0.27358	79.50181	7.44
(E21)...E23Q	56.39029	-137.91575	-0.06632	81.45914	7.50
(Q21)...E23Q	57.07932	-137.98829	-0.97050	79.93847	8.21
(E41)...E42Q	64.36035	-139.94819	0.04432	75.63216	6.16
(Q41)...E42Q	63.92586	-140.09838	-0.30096	75.87156	5.86
(E41)...D43N	61.78881	-135.53975	-0.27822	73.47272	10.64
(Q41)...D43N	62.42679	-133.52279	-0.46981	70.62619	9.62
(D43)...E46Q	61.07022	-139.94670	-0.16233	78.71415	5.96
(N43)...E46Q	60.45539	-136.06756	0.12897	75.74114	6.80

S5 Negative values of $\Delta\Delta G$ originate from interactions between vicinal acidic amino acids

A negative value of $\Delta\Delta G_{\text{decharge}}$ means that decharging an acidic amino acid, X, is more unfavorable when the neighbor is also an acidic amino acid (Equation 3):

$$(-)\text{X}_a \xrightarrow{\Delta G_{(-)\text{decharge}}} (-)\text{X}_a^0 \tag{3}$$

that when the neighbor is electrically neutral (Equation 4):

$$(0)\text{X}_a \xrightarrow{\Delta G_{(0)\text{decharge}}} (0)\text{X}_a^0. \tag{4}$$

Therefore, a negative value of $\Delta\Delta G_{\text{decharge}}$ can arise either from i) unexpected *stabilizing* electrostatic interactions between the two vicinal acidic amino acids – initial state in Equation 3 – or ii) *destabilizing* electrostatic interactions between vicinal acidic and neutral amino acids – initial state in Equation 4 – strong enough to compensate the expected electrostatic repulsion between two negative amino acids.

Indirect insight from the charging step indicates that the first possibility is the one at play here. The charging step indicates it is more favorable to introduce atomic charges in the final amino acid Y (which is net neutral) when its neighbor has a negative charge, corresponding to Equation 5,

$$(-)\text{Y}_a^0 \xrightarrow{\Delta G_{(-)\text{charge}}} (-)\text{Y}_a \tag{5}$$

than when its neighbor is neutral, corresponding to Equation 6

$$(0)\,\text{Y}_a^0 \xrightarrow{\Delta G_{(0)\text{charge}}} (0)\,\text{Y}_a \tag{6}$$

Considering that i) the electrostatic interaction between two neutral amino acids is well-described as a dipole-dipole interaction; ii) this interaction decays as $1/r^3$ where r is the distance between the dipoles; iii) the amino acids in question are at least 5 Å apart; iv) we are considering interactions at high KCl concentrations, we can assume that the electrostatic energy between 2 neutral amino acids, corresponding to the "products" in Equation 6, is approximately zero; our PMF calculations confirm that this assumption holds. Consequently, $-\Delta\Delta G_{\text{charge}} \approx \Delta G_{(-)\text{charge}}$ is a reasonable estimate of the electrostatic interaction between a neutral (N or Q) and an acidic (D or E) amino acid; this value is negative, indicating they have stabilizing interactions. These results indicate that synergistic effects between neighboring acidic amino acids, as measured by $\Delta\Delta G$, reflect electrostatically favorable interactions between vicinal acidic amino acids.

S6 Replica Exchange Molecular Dynamics (REMD)

S6.1 Computational details of REMD simulation

We performed a REMD simulation to sample the unfolded state ensemble of halophilic protein L. (pdb ID: 2KAC) at the high salt concentration of $b_{\mathrm{KCl}} = 2$ mol·kg^{-1}. Our aim was to obtain a representative unfolded configuration of this protein for subsequent free energy studies of the synergistic effect, to enable comparisons between the folded and the unfolded states.

The simulations were performed using the AMBER 2018 simulation package[14], using the pmemd engine on GPUs. The only exception was the l-bfgs minimization step, which was performed on the Sander engine of the AMBER simulation package because it is not available on the pmemd engine. All the simulation boxes are cubic with periodic boundary conditions applied in the XYZ directions. We set a non-bonded potential cutoff distance at 12 Å for vdW and electrostatic interactions. Beyond this cutoff distance, the electrostatic interactions are calculated with the particle mesh Ewald (PME) scheme with a grid spacing of 1.0 Å, and 4th order of interpolation[2]. Long-range dispersion corrections were applied to both the energy and pressure. The crystal structure of the halophilic protein with pdb ID 2KAC was placed in a simulation box with an edge length of $\approx$ 110 Å; the box was filled with TIP3P water and sufficient ions to obtain an electrically neutral system with the desired molality of KCl. All bonds with H-atoms were constrained using the SHAKE algorithm[16] in the NpT ensemble and REMD simulation. Four initial minimizations cycles, each with 2500 steps of the Steepest-Descent algorithm and 7500 steps of the Conjugate Gradient algorithm while using progressively weaker harmonic restraints (with bond constants 500, 300, 100, and 50 kcal·mol^{-1}· Å^{-2}) on the protein atoms were performed to remove the bad contacts that arose from the experimental structure and in the process of adding ions and water to the system. Afterwards, another minimization using the l-bfgs algorithm without any constraints or restraints was performed for 10000 steps.

The system was heated over 50 ns in the canonical ensemble (NVT) using the Langevin thermostat with a collision frequency of 1.0 ps^{-1} to slowly increase the temperature of the system from 0 to 800 K to denature the protein. Then another heating simulation in the canonical ensemble (NVT) was performed for 2.5 ns, during which the temperature was decreased again to reach 298 K using the Langevin thermostat with the same collision frequency. The SHAKE algorithm was not used for these two heating simulations, so consequently, a timestep of 1 fs was used. The system was equilibrated for a total of 10 ns, in 10 steps of 1 ns simulations, to equilibrate the density in the isothermal-isobaric ensemble (NpT) using the Berendsen barostat[1], and the Langevin thermostat, to keep the average temperature at 298 K, and the pressure at 1 bar.

The REMD simulation was performed in the NVT ensemble. The starting configuration was extracted from the last 1 ns NpT equilibration simulation, taking care to choose a configuration in which the density of the box was closest to the average. Also for the REMD simulation, we used the Langevin thermostat with a collision frequency of 1 ps^{-1}. The replica temperatures are given in section S6.2. Replica exchanges were attempted every 2500 MD steps; a total of 20000 exchanges were attempted. Consequently, each replica was simulated for $2500{\times}20000 = 5 \times 10^7$ MD steps which, considering the timestep of 2 fs, results in 100 ns of simulation time for each replica.

At such high temperatures, ($> 500\ K$) unwanted rotations around the peptide bond might occur, leading to non-physical chiralities. To prevent this, we used harmonic chirality restraints with force constants of 50 kcal·mol^{-1}·Å^{-2} on the backbone ω dihedrals (defined by the backbone atoms C_α, C, N, C_α), to retain it in *trans* configuration to keep it planar, and do not allow its rotation to *cis* configuration.

In the end, we extracted a trajectory from only those frames of all replica trajectories that corresponded to 298 K and performed the subsequent analysis on this trajectory.

S6.2 Temperature replicas used in REMD simulation

A total of 221 replica with different temperatures was used as 298.15, 298.98, 299.82, 300.65, 301.49, 302.33, 303.18, 304.02, 304.87, 305.71, 306.56, 307.42, 308.27, 309.12, 309.98, 310.84, 311.70, 312.57, 313.43, 314.30, 315.17, 316.04, 316.91, 317.79, 318.67, 319.55, 320.43, 321.31, 322.20, 323.09, 323.97, 324.87, 325.76, 326.66, 327.55, 328.45, 329.35, 330.26, 331.16, 332.07, 332.98, 333.89, 334.81, 335.73, 336.64, 337.57, 338.49, 339.41, 340.34, 341.27, 342.20, 343.13, 344.07, 344.97, 345.91, 346.86, 347.80, 348.75, 349.69, 350.64, 351.60, 352.55, 353.51, 354.47, 355.43, 356.39, 357.36, 358.33, 359.30, 360.27, 361.24, 362.22, 363.20, 364.18, 365.17, 366.15, 367.14, 368.13, 369.12, 370.12, 371.11, 372.11, 373.12, 374.12, 375.12, 376.13, 378.16, 37.16, 380.18, 381.20, 382.22, 383.25, 384.28, 385.30, 386.34, 387.37, 388.41, 389.44, 390.48, 391.53, 392.57, 393.62, 394.68, 395.73, 396.79, 397.84, 398.90, 399.97, 401.03, 402.10, 403.18, 404.25, 405.33, 406.40, 407.48, 408.57, 409.65, 410.74, 411.83, 412.92, 414.02, 415.12, 416.22, 417.32, 418.42, 419.53, 420.64, 421.75, 422.87, 423.99, 425.11, 426.23, 427.36, 428.48, 429.62, 430.75, 431.89, 433.03, 434.17, 435.31, 436.46, 437.61, 438.76, 439.91, 441.07, 442.2, 443.39, 444.56, 445.73, 446.90, 448.07, 449.25, 450.43, 451.61, 452.79, 453.98, 455.17, 456.36, 457.55, 458.75, 459.95, 461.15, 462.36, 463.57, 464.78, 466.00, 467.21, 468.44, 469.66, 470.88, 472.11, 473.35, 474.57, 475.81, 477.05, 478.29, 479.54, 480.79, 482.04, 483.29, 484.55, 485.81, 487.07, 488.34, 489.61, 490.88, 492.15, 493.43, 494.71, 495.99, 497.28, 498.57, 499.86, 501.16, 502.46, 503.76, 505.06, 506.37, 507.68, 508.99, 510.31, 511.63, 512.95, 514.28, 515.61, 516.94, 518.27, 519.61, 520.95, 522.29, 523.64, 524.99, 526.35, 527.70, 529.06, 530.43, 531.79, 533.16, 534.53, 535.91, 537.00. This means a total of 22.1 μs of simulation. This temperature distribution was taken from the website `http://folding.bmc.uu.se/remd-temperature-generator/`[17].

S6.3 Evaluating the quality of REMD simulations

To examine whether a good quality REMD simulation was achieved with the temperature distribution, the number of replicas, and the simulation details used in our study, we examine the acceptance rate of exchanges between adjacent pairs, and the temperature of individual replicas. Figure S8 shows the acceptance rate of the exchanges between replicas, considering up and down exchanges. The acceptance rate varies between 40% and 56%, indicating that exchanges between neighboring replicas were likely in all parts of the temperature distribution. Also, the acceptance rates are large enough (> 40%), leading to a sufficient number of exchanges during the simulation.

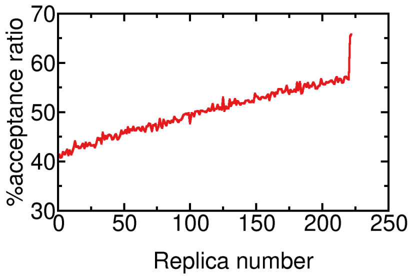

Fig. S8 Fraction of successful exchange attempts (exchange rate) for each replica.

Figure S9 shows the temperature distribution for one of the replicas with an initial temperature of 298.98 K. This replica visited higher temperatures at the end of the REMD simulation, but clearly has not experienced the desired random walk in temperature space. Doing so would require a much longer simulation time, beyond our resources.

Analysis of the protein structure (not shown) at 298 K indicates that we have sampled a subset of the unfolded structure ensemble that is rich in collapsed structures but contains few expanded structures. This level of sampling of the unfolded state ensemble is sufficient for our purpose: to obtain a structure

that is representative of the ensemble of unfolded but collapsed structures, which can be used to study the incidence of synergistic interactions between acidic amino acids in denatured protein conformations.

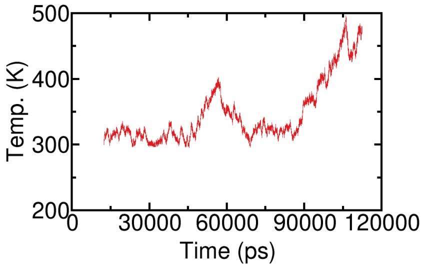

Fig. S9 Temperature distribution for the replica with the starting temperature of 298.98 K.

S7 Potential of mean force of 2-body systems

Figure S10 shows a representative configuration of two aspartates at the mininum of the PMF shown in Figure 6A in the main text. The gas phase interaction energy of the two amino acids in this conformation is negative (-2.63 kJ·mol^{-1}) because the LJ component (-5.1 kJ·mol^{-1}) largely overcomes the electrostatic repulsion (+2.44 kJ·mol^{-1}). These results confirm that the minimum of the PMF is due to the proximity of the CH_2 and CH_3 groups, which enables strong LJ attraction.

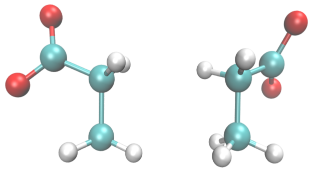

Fig. S10 Representative configuration of the D-D system with $\theta_1 = \theta_2 = 90°$ for $\xi \approx 4$ Å (the distance between the two α carbons), i.e., at the minimum of the PMF shown in Fig. 6A in the main text. The two carboxylates preferentially point away from each other whereas the CH_2 and CH_3 groups are very close.

Figure S11 shows the potential of mean force between two aspartates, calculated as a function of the distance of the carboxylate carbons. This curve differs quantitatively from that calculated based on the distance between the two alpha carbons (shown in Figure 6A) but retains similar features, such as the minimum at short separations.

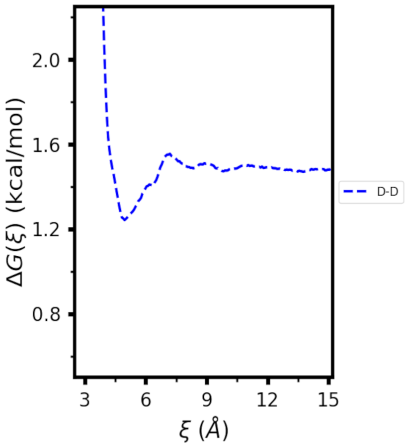

Fig. S11 Potential of mean force as a function of $C_\gamma \cdots C_\gamma$ distance of the D-D pair with parallel side chains (restraint angles $\theta_1 = \theta_2 = 90°; \phi = 0°$), at b_{KCl}= 2 mol·kg^{-1} (2m). The minimum near $\xi \approx$4 Åresults from LJ interactions, that overcome electrostatic repulsion.

S8 Potential of mean force of 3-body systems

We performed PMF calculations for a system of 3 side chains, as a function of the distance between one of the side chains from the other two. We considered only the case where the side chains are parallel to each other and perpendicular to the plane of the three C_αs, at b_{KCl} = 2 mol·kg^{-1}. We then calculated $\Delta\Delta G$ similarly as for the two-body system:

$$\Delta\Delta G(\xi) = \Delta G_{\text{D-D--D}} + \Delta G_{\text{D-N--N}} - 2 \times \Delta G_{\text{D-D--N}} \quad (7)$$

Figure S12 shows $\Delta\Delta G(\xi)$ for the 3-body system. The interactions remain qualitatively similar to those in the analogous 2-body system, with maximum repulsion being observed for a triplet of negative charges at $\xi = 6$ Å. The value of $\Delta\Delta G(\xi)$ is never negative, indicating the synergistic effect does not occur in this 3-body system either.

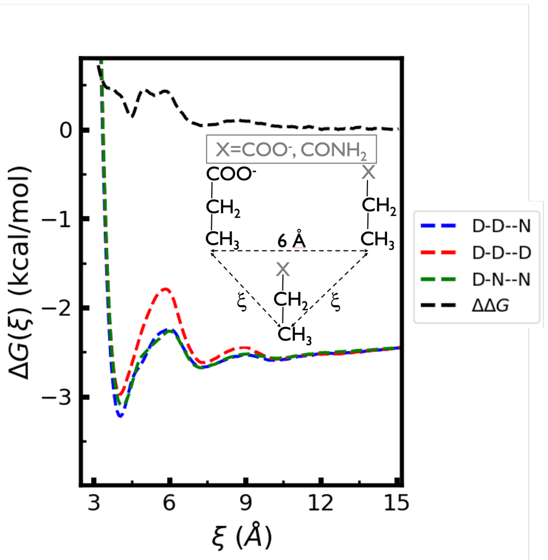

Fig. S12 Potential of mean force ($\Delta G(\xi)$) as a function of the distance ξ between a side chain and two other side chains at fixed positions, for the indicated side chain systems at b_{KCl} = 2 mol·kg^{-1} (2m).

References

1 H. J. C. Berendsen, J. P. M. Postma, W. F. van Gunsteren, A. DiNola and J. R. Haak, *J. Chem. Phys.*, 1984, **81**, 3684–3690.

2 T. Darden, D. York and L. Pedersen, *J. Chem. Phys.*, 1993, **98**, 10089–10092.

3 H. J. C. Berendsen, D. van der Spoel and R. van Drunen, *Comput. Phys. Commun.*, 1995, **91**, 43–56.

4 D. Van Der Spoel, E. Lindahl, B. Hess, G. Groenhof, A. E. Mark and H. J. C. Berendsen, *J. Comput. Chem.*, 2005, **26**, 1701–1718.

5 W. F. van Gunsteren and H. J. C. Berendesen, *Mol. Simul.*, 1988, **1**, 173–185.

6 B. Hess, H. Bekker, H. J. C. Berendsen and G. E. M. Fraaije, *J. Comput. Chem.*, 1997, **18**, 1463–1472.

7 M. Parrinello and A. Rahman, *J. Appl. Phys.*, 1981, **52**, 7182–7190.

8 S. Nosé and M. L. Klein, *Mol. Phys.*, 1983, **50**, 1055–1076.

9 M. Garton, C. Corbi-Verge, Y. Hu, S. Nim, N. Tarasova, B. Sherborne and P. M. Kim, *PROTEINS: Structure, Function, and Bioinformatics*, 2019, **87**, 236–244.

10 G. J. Rocklin, D. L. Mobley, K. A. Dill and P. H. Hünenberger, *J. Chem. Phys.*, 2013, **139**, year.

11 P. H. Hünenberger and J. A. McCammon, *Chem. Phys.*, 1999, **110**, 1856–1872.

12 M. A. Kastenholz and P. H. Hünenberger, *J. Phys. Chem. B*, 2004, **108**, 774–788.

13 M. A. Kastenholz and P. H. Hünenberger, *Chem. Phys.*, 2006, **124**, year.

14 D. Case, I. Ben-Shalom, S. Brozell, D. Cerutti, T. Cheatham III, V. Cruzeiro, T. Darden, R. Duke, D. Ghoreishi, M. Gilson, H. Gohlke, A. Goetz, D. Greene, R. Harris, N. Homeyer, Y. Huang, S. Izadi, A. Kovalenko, T. Kurtzman, ... and P. A. Kollman, *Amber 18*, University of California, San Francisco, 2018.

15 U. Essmann, L. Perera, M. L. Berkowitz, T. Darden, H. Lee and L. G. Pedersen, *Chem. Phys.*, 1995, **103**, 8577–8593.

16 J.-P. Ryckaert, G. Ciccotti and H. J. Berendsen, *J. Comput. Phys.*, 1977, **23**, 327–341.

17 A. Patriksson and D. Van Der Spoel, *Phys. Chem. Chem. Phys.*, 2008, **10**, 2073–2077.
