# Supplementary material for: Prevalence and mechanism of synergistic carboxylate-cation-water interactions in halophilic proteins
Source: Biophys J. 2023 May 12;122(12):2577–89. doi: 10.1016/j.bpj.2023.05.011 (PMC10323026; doi:10.1016/j.bpj.2023.05.011)
Supplement: Document S2. Article plus supporting material [file mmc2.pdf]

Biophysical Journal
Article

Prevalence and mechanism of synergistic carboxylate-cation-water interactions in halophilic proteins

Hosein Geraili Daronkola[1] and Ana Vila Verde[1,*]
[1]Max Planck Institute of Colloids and Interfaces, Department of Theory & Bio-Systems, Potsdam, Germany

ABSTRACT The cytoplasmic proteins of some halophilic organisms remain stable and functional at multimolar concentrations of KCl, i.e., under conditions that most mesophilic proteins cannot withstand. Their stability arises from their unusual amino acid composition. The most dramatic difference between halophilic and mesophilic proteins is that the former are rich in acidic amino acids. It has been proposed that one of the evolutionary driving forces for this difference is the occurrence of synergistic interactions between multiple acidic amino acids at the surface of the protein, the potassium cations in solution, and water. We investigate this possibility with molecular dynamics simulations, using high-quality force fields for the protein-water, protein-ion, and ion-ion interactions. We create a rigorous thermodynamic definition of interactions between acidic amino acids on proteins that can be used to distinguish between synergistic, noninteracting and interfering interactions. Our results demonstrate that synergistic interactions between neighboring acidic amino acids in halophilic proteins are frequent at multimolar KCl concentration. Synergistic interactions have an electrostatic origin, and are associated with stronger water-to-carboxylate hydrogen bonds than for acidic amino acids without synergistic interactions. Synergistic interactions are not observed in minimal systems of carboxylates, indicating that the protein environment is critical for their emergence. Our results demonstrate that synergistic interactions are neither associated with rigid amino acid orientations nor with highly structured and slow moving water networks, as had been originally proposed. Moreover, synergistic interactions can also be found in unfolded protein conformations. However, because these conformations are only a small subset of the unfolded state ensemble, synergistic interactions should contribute to the net stabilization of the folded state.

SIGNIFICANCE X-ray crystallography and NMR studies have suggested that acidic amino acids in folded halophilic proteins interact synergistically with water and cations at molar KCl concentrations, stabilizing the folded protein. We provide, for the first time, an operational definition of synergistic interactions between acidic amino acids that enables their quantification in molecular simulations. This definition can easily be adapted to investigate synergistic interactions between other amino acids. The results confirm that synergistic interactions exist at high KCl concentration and should stabilize the folded protein. They have an electrostatic origin and are associated with strong water-to-carboxylate hydrogen bonds. The results suggest nevertheless that other mechanisms may contribute to the evolutionary driving force behind the abundance of acidic amino acids in halophilic proteins.

INTRODUCTION

A few environments on Earth have physical-chemical properties far from the typical earthly environment; nevertheless, life thrives in these extreme environments as well. One striking example of such places is the Dead Sea, with its multi molar concentration of bromide and chloride salts, i.e., much higher than the typical salt concentration ($\approx 0.7\ \mathrm{mol\cdot dm^{-3}}$) in oceans. Microorganisms living at high salt concentrations are a specific type of extremophile; they are called halophiles (from the Greek word for "salt-loving") (1). Halophilic microorganisms are mainly archea (2) and bacteria (3), but halophilic fungi, algae, protozoa, and multicellular eukaryotes also exist (4,5). To prevent rupture of their membrane due to the high concentration of salt in the environment, and therefore a high osmotic

Submitted February 23, 2023, and accepted for publication May 9, 2023.

*Correspondence: ana.araujo-vila-verde@uni-due.de

Hosein Geraili Daronkola and Ana Vila Verde's present address is University of Duisburg-Essen, Faculty of Physics, Duisburg, Germany.

Editor: Lucie Delemotte.

https://doi.org/10.1016/j.bpj.2023.05.011

© 2023 Biophysical Society.

This is an open access article under the CC BY license (http://creativecommons.org/licenses/by/4.0/).

pressure, halophiles accumulate osmolytes inside their cytoplasm up to the same concentration as the external environment (1). For some halophiles, the main osmolyte is KCl; these halophiles boast cytoplasmic KCl concentrations in the multimolar range, far higher than the 0.15 mol$\cdot$ dm^{-3} that is typical of nonhalophilic organisms (termed mesophiles). Halophilic proteins (of halophiles where KCl is the main osmolyte) thus remain soluble, structurally stable, and functional (1,4,6–8) under conditions that proteins of mesophiles (i.e., mesophilic proteins) cannot typically tolerate (1,9–12).

Halophilic and mesophilic proteins differ in their amino acid composition (7). Revealing and quantifying how amino acid composition impacts protein-solvent, protein-ion, and protein-protein interactions so proteins remain stable and functional at high KCl concentration is important from a fundamental perspective, because halophilic environments are ancient and are candidates for proteogenesis (the origin of polypeptides) and abiogenesis (the origin of living systems) (13–15). The amino acids most abundant in halophilic proteins are part of the prebiotic set of amino acids; polypeptides formed from this set have the ability to fold when under halophilic conditions (13–15). Clarifying the physical-chemical mechanisms that result in structurally stable and functional proteins under halophilic conditions is thus critical for a broader understanding of evolution and of protein folding. It is also important from a biotechnological perspective, to develop methods to rationally determine the optimal and minimal modifications that must be introduced in mesophilic enzymes (e.g., for production of chiral drug molecules (16), for production of biofuels (17)) so they remain functional at high NaCl concentration. Enzymes with this ability are advantageous in a world where fresh water resources are increasingly scarce.

Mesophilic and halophilic proteins differ substantially in composition and structure. The most noticeable difference between them is that halophilic proteins are highly depleted of positively charged residues, and are highly enriched in negatively charged amino acids, especially aspartic acid, located on the surface of the protein (4,6,7,18,19). Halophilic proteins are thus on average substantially negatively charged. The physical mechanisms making up the evolutionary driving force for this characteristic are not yet well-understood. Multiple explanations have been proposed, as we described elsewhere (20); here we provide only a short summary. At high salt concentration, electrostatic interactions are strongly screened. A seemingly obvious explanation for the excess acidic amino acids in halophilic proteins is that a high net protein charge is necessary for sufficient electrostatic repulsion to prevent protein aggregation, i.e., to increase protein solubility (4,21). However, the fact that enzyme activity, structural stability, and association of subunits strongly depend on salt concentration beyond 0.5 mol$\cdot$dm^{-3}, even though charge screening is largely complete by this concentration, indicates that, while maintenance of solubility may well be one of their roles, acidic amino acids should have other roles as well (1). The *solvent-only stabilization* model claims that acidic amino acids, which have the highest water-binding ability of all amino acids (22), are necessary to keep the protein hydrated by competing with the ions in solution for available water (2,23). However, our previous simulation work (20) does not support that scenario. Our simulations indicate that the amount of hydration water for any given protein is barely affected by changing the KCl concentration from 0.15 to 2 mol$\cdot$dm^{-3} (20).

In this work, we investigate another proposed explanation for the abundance of acidic amino acids in halophilic proteins: the *ion-solvent stabilization model*. This hypothesis claims that hydrated ion networks form around the surface of the folded protein, enabled by specific arrangements of carboxyl groups that attract hydrated potassium ions and which thus induce synergistic interactions between the acidic amino acids (4,24–26). This hypothesis has been described in qualitative terms only. Because analogous arrangements of acidic amino acids cannot form (or at least are not dominant) in the ensemble of unfolded protein structures, the folded structure of halophilic proteins is stabilized relative to the unfolded state (4,24–26). This hypothesis is supported by the presence of highly ordered protein hydration shells in some of the few halophilic proteins that have been crystallized (27). Synergistic hydrated ion networks would explain why some halophilic proteins have higher hydration levels than predicted based on their content in acidic amino acids alone, and why halophilic proteins bind larger amounts of salt than mesophilic proteins (4,24–26,28). Such an ordered solvation layer around the protein is proposed to function as a barrier that prevents aggregation of halophilic proteins at high salt concentration (4,24–26). This hypothesis was further supported by measurements of water translational dynamics in the cytoplasm of halophilic and nonhalophilic bacteria using quasielastic neutron spectroscopy, which were interpreted as indicating that water near halophilic proteins had extremely slow translational dynamics (29). However, this interpretation of the quasielastic neutron spectroscopy measurements was subsequently challenged by another experimental study (30), and by our previous simulation work, which demonstrated that the translational dynamics of water of hydration of mesophilic and halophilic proteins are quite similar (20).

We use simulations to determine whether synergistic water + potassium + carboxylate networks indeed arise in halophilic proteins and contribute toward the stability of the folded protein structure relative to the unfolded state. We use force fields for the carboxylate-potassium, carboxylate-amine, and carboxylate-water interactions previously optimized by us to correctly capture these interactions, a critical aspect of this work (20,31). With the optimized parameters, the models reproduce the KCl activity derivative of aqueous potassium acetate solutions up to $b_{KCH_3COO} = 2$

$mol \cdot kg^{-1}$, the carboxylate-potassium distances visible in the crystal structure of a protein, the difference between the experimental hydration free energies of the carboxylate and the chloride ions, and the osmotic pressure of concentrated glycine solutions. Although multiple references to synergistic or cooperative effects involving acidic amino acids in halophilic proteins can be found in the literature, a quantifiable definition has not been offered. We thus start by defining synergistic and nonsynergistic (i.e., interfering) interactions between acidic amino acids in a precise and quantifiable manner. The minimum system in which synergistic interactions between acidic amino acids and the ions in solution can exist is that involving two neighboring amino acids. We investigate whether synergistic interactions exist between pairs of neighboring amino acids on halophilic proteins, and also in model systems composed of dimers or trimers of mimics of amino acid side chains. Our results make clear that synergistic interactions between neighboring acidic amino acids are indeed possible in proteins at high KCl concentration, and give insight into the physical mechanisms behind them.

METHODS

All simulations used the TIP3P water model (32), and modified versions of the AMBER ff14SB (33,34) force field for proteins and of the potassium and chloride parameters of Joung and Cheatham (35) for TIP3P water. The original force fields significantly overestimate the interaction between carboxylates and amines and between carboxylates and the potassium ion. To overcome these shortcomings, we modified the Lennard-Jones (LJ) interactions between carboxylate and water, carboxylate and amines, and carboxylate and potassium ions as described in our previous work (20,31).

Free energy calculations

Free energies associated with the mutation of acidic residues—Asp (D) or Glu (E)—to their neutral counterparts—Asn (N) or Gln (Q), respectively—were calculated using thermodynamic integration as implemented in the pmemd GPU engine of the AMBER 18 molecular dynamics package (36), using a dual-topology approach and soft core potentials. Calculations were performed for halophilic proteins with Protein Data Bank identifying codes (PDB) PDB: 1DOI (2Fe-2S ferredoxin from *H. marismortui* (23)), PDB: 2KAC (protein L mutant Kx6E (37)), and PDB: 2ITH (dihydrofolate reductase from *H. volcanii* (38)). Folded proteins PDB: 1DOI and PDB: 2KAC were simulated at two different salt concentrations: $b_{KCl} = 0.15\ mol \cdot kg^{-1}$, corresponding to mesophilic conditions (the control situation), and $b_{KCl} = 2\ mol \cdot kg^{-1}$, corresponding to halophilic conditions. The folded dihydrofolate reductase and the unfolded protein L were simulated at the higher salt concentration to increase the number and diversity of systems under halophilic conditions, for which synergistic effects have been proposed to occur. Given the very high computational cost of these calculations, these two systems were not simulated at low salt concentration. The starting structure of each folded protein for the free energy calculations was the last saved configuration of 1 μs production runs at the desired salt concentration, reported in our previous publication (20). The starting structure for the simulations with the unfolded protein L was obtained as described in supporting material, section 6. The simulation boxes (Fig. 1) are cubic, with edge length $\approx$ 100 Å.

To choose appropriate pairs of acidic amino acids for the mutation study, we first selected residues with a minimum solvent-accessible surface area of

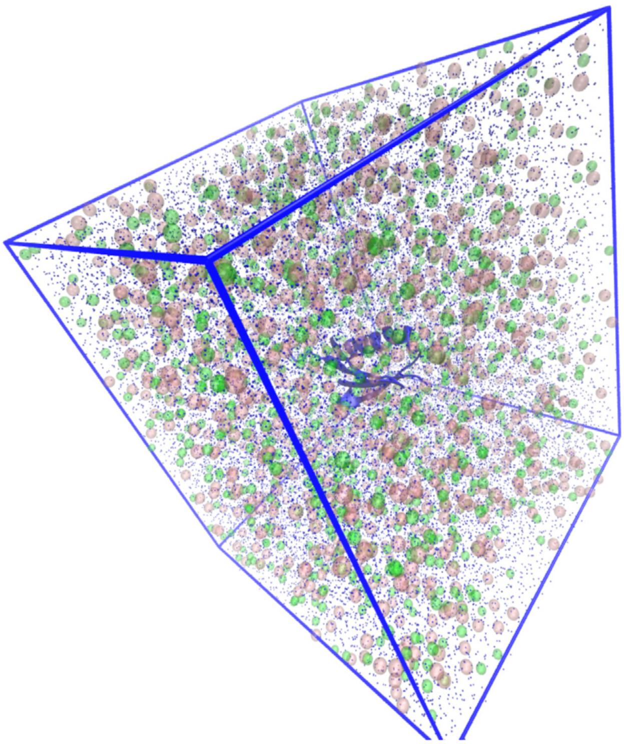

FIGURE 1 A snapshot of the simulation box of protein L in an aqueous solution of KCl at $b_{KCl} = 2\ mol \cdot kg^{-1}$. Protein L, dark blue; K^{+}, transparent pink spheres; Cl^{-}, transparent green spheres; oxygen atoms of water molecules, blue dots. To see this figure in color, go online.

50 Å^2 using the package *chimera* (39), thus ensuring they were solvent exposed. Of this set, we selected amino acid pairs for which the distance between their carboxylate carbons was below 7 Å at $t = 0$.

Simulations were performed in three steps, described in Eq. 1 for the mutation of a generic amino acid X into amino acid Y. In the equation, a denotes the position of the residue in the amino acid sequence. Y_a^0 and X_a^0 indicate nonphysical forms of the amino acid where all atomic charges are set to zero. The free energy of mutation, ΔG_{XaY}, is equal to the sum of the three partial terms shown in Eq. 1. This free energy reflects only the different nonbonded interactions of the initial- and final-state amino acids with the solvent (water and ions) and the surrounding protein.

$$\begin{array}{ccc} X_a & \xrightarrow{\Delta G_{XaY}} & Y_a \\ \Delta G_{decharge}\ (1) \downarrow & & \uparrow (3)\ \Delta G_{charge} \\ X_a^0 & \xrightarrow[\Delta G_{vdW}]{(2)} & Y_a^0 \end{array} \tag{1}$$

Complete details of the free energy simulations are given in supporting material, section 2.1; here we summarize only the most relevant aspects. The protein backbone atoms were restrained to their initial position using a harmonic restraint with a force constant of 35 $kcal \cdot mol^{-1} \cdot Å^{-2}$. Doing so is indispensable to obtain reproducible values of ΔG_{XaY} for each mutation. Our tests, discussed in supporting material, section 3, indicate that, without backbone restraints, small conformational changes away from the mutation site affect the value of ΔG_{XaY} by a few $kcal \cdot mol^{-1}$. Changes of

this magnitude are similar to those that may arise from the synergistic effect, and would thus make results impossible to interpret. The introduction of backbone restraints eliminates this difficulty. We emphasize that the energy of the restraints does not contribute to $\partial V/\partial\lambda$, and therefore does not contribute to the mutation free energy.

The Python tool *alchemical-analysis* (40) was used to calculate the free energy difference for each of the steps in Eq. 1. This tool calculates multiple free energy estimates, using different methods, from the simulation data. In all cases, the first 1 ns of the production simulations was ignored and considered as equilibration time. The values reported here were estimated integrating $\partial V/\partial\lambda$ using a natural cubic spline ("TI-3"). A detailed analysis of computational accuracy and choice of the best estimator for our system is given in supporting material, section 2.1.1.

Potential of mean force calculations

To generate minimal models of the side chains of aspartate and of asparagine, we replaced the amino ($-NH_3^+$) and carboxylate ($-CO_2^-$) functional groups attached to the C_α of the zwitterionic form of the amino acids by two hydrogen atoms, to form a $-C_\alpha H_3$ group. The three hydrogen atoms attached to the C_α were given identical charges so that the net charge of the side chain model is zero (for asparagine) or -1 (for aspartate). Finally, the van der Waals (vdW), bond, angle, and dihedral parameters for interactions involving the newly added hydrogen atoms and the rest of the side chain atoms were given the same values as those of the standard hydrogen bonded to C_α in the respective amino acid. The side chain models are shown in Fig. 2.

All umbrella sampling simulations from which the potential of mean force (PMF) curves were obtained were performed using the GROMACS 2020 simulation package (41,42). Complete simulation details are given in supporting material, section 2.1; here we summarize only the most relevant simulation choices. The two-body PMFs were calculated as a function of the distance, ξ, between the C_α of the side chains. For all cases, the side chains were kept approximately coplanar by restraining the dihedral φ defined by atoms $C_{\beta1}$-$C_{\alpha1}$-$C_{\alpha2}$-$C_{\beta2}$ of the two amino acids (denoted by the subscripts 1 and 2) to $\varphi = 0^\circ$. Calculations were performed for different relative orientations of the side chains, enforced by restraining the angles θ_1 and θ_2 to different values; θ_1 is defined by atoms $C_{\beta1}$-$C_{\alpha1}$-$C_{\alpha2}$; θ_2 is defined by atoms $C_{\alpha1}$-$C_{\alpha2}$-$C_{\beta2}$. The collective variables used in the two-body PMF calculations are illustrated in Fig. 2 *A*. The two-body PMF was calculated for three different systems—D-D, N-N, and D-N—in explicit water and KCl at $b_{KCl} = 0.15$ and 2 mol$\cdot$ kg^{-1}.

The three-body PMF was calculated as a function of the distances, ξ_1 and ξ_2, of one amino acid to the other two, as illustrated in Fig. 2 *B*. The C_αs of two of the amino acids are restrained to fixed positions in space so they are on average 6 Å apart. The position of the C_α of the third amino acid is governed by the restraints applied to ξ_1 and ξ_2. Six angular and three dihedral restraints (not shown in Fig. 2 *B*) analogous to those employed in the two-body system were used to keep the side chains approximately perpendicular to the plane defined by the three C_αs, by setting the angle equilibrium values to 90° and the dihedral ones to 0°. PMFs were calculated for three different systems—D-D–D, D-D–N, and D-N–N—in explicit water and KCl at $b_{KCl} = 2$ mol$\cdot$kg^{-1}. The WHAM (43) analysis program implemented in GROMACS was used to compute the PMF curves from the output of the umbrella simulations.

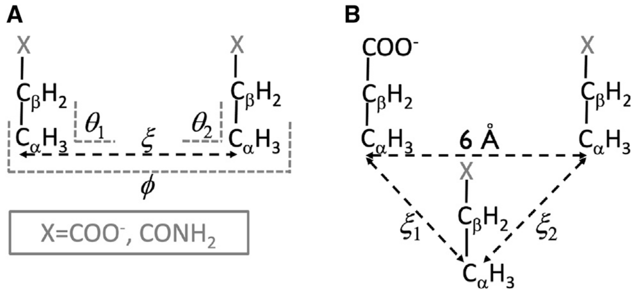

FIGURE 2 Systems and collective variables used in the potential of mean force calculations; $X = COO^-$ for the model aspartate side chain and $X = CONH_2$ for the model asparagine side chain. (*A*) Two-body systems, showing the reaction coordinate ξ and the restrained angles (θ_1 and θ_2) and dihedral (φ). (*B*) Three-body systems, showing the reaction coordinates ξ_1 and ξ_2; angle and dihedral restraints (not shown) analogous to those applied in the two-body system were used to keep the side chains parallel to each other and perpendicular to the plane defined by the three C_αs.

RESULTS

Defining and quantifying synergistic interactions between acidic amino acids

As summarized above, the ion-solvent stabilization model claims that synergistic interactions between spatially close acidic amino acids, mediated by hydrated potassium cations, stabilize folded halophilic proteins at high KCl concentrations (24,26,44). To investigate this effect in simulations, a precise and quantifiable definition of synergistic interactions between acidic amino acids is necessary. The smallest system in which synergistic interactions between acidic amino acids can exist consists of two acidic residues. With this in mind, we designed a simulation protocol to assess the existence and magnitude of these synergistic interactions by a difference of mutation free energies. A pair of solvent-exposed acidic amino acids in a protein, in positions *a* and *b* of the amino acid sequence, is selected. We first calculate the free energy of mutating of one of them to the neutral natural amino acid that most closely resembles it: aspartate (D) is mutated to asparagine (N) and glutamate (E) to glutamine (Q). This mutation is schematically represented as:

$$(-)X_a \xrightarrow{\Delta G_{(-)XaY}} (-)Y_a \tag{2}$$

Equation 2 indicates that amino acid X in position *a* is mutated to amino acid Y; the minus sign in parentheses $(-)$ emphasizes that this mutation is done in the presence of a particular negative neighbor in position *b*. The free energy $\Delta G_{(-)XaY}$ contains only the contribution of the interactions between the environment (i.e., the water, ions in solution, and the rest of the protein) and the mutated residues to the mutation free energy, as detailed in the methods. The structure and size of the neutral amino acid Y (Q or N) are very similar to that of X (E or D) as can be seen in Fig. S1, so the initial and final states are expected to have similar vdW interactions with their environment, i.e., these differences will contribute minimally to $\Delta G_{(-)XaY}$. Our results below confirm that this assumption holds. $\Delta G_{(-)XaY}$ is thus dominated by electrostatic interactions with the solvent (i.e., water + ions). These interactions are much more favorable for charged amino acids than for uncharged ones (45), so this free energy is positive (i.e., the mutation is unfavorable). If solvent-mediated synergistic electrostatic

interactions exist between the two acidic amino acids, they will be present in the initial state but not the final state of the system, and will thus affect the value of $\Delta G_{(-)\mathrm{X}a\mathrm{Y}}$.

In a second step, we calculate the free energy associated with the same mutation, but now for a modified version of the protein where D or E in position b was replaced by N or Q, as appropriate, before the calculation is performed. This second mutation is schematically represented as

$$(0)\mathrm{X}_a \xrightarrow{\Delta G_{(0)\mathrm{X}a\mathrm{Y}}} (0)\mathrm{Y}_a \quad (3)$$

where (0) emphasizes that the neighboring amino acid is electrically neutral.

The difference

$$\Delta\Delta G = \Delta G_{(0)\mathrm{X}a\mathrm{Y}} - \Delta G_{(-)\mathrm{X}a\mathrm{Y}} \quad (4)$$

gives direct insight into the presence or absence of synergistic interactions between amino acids, and quantifies the magnitude of this effect. If amino acids a and b interact very weakly (e.g., if they are spatially distant), $\Delta\Delta G = 0$. If they are nearby, conventional understanding would suggest that two charged amino acids repel each other. Moreover, this repulsion should be more intense than any interactions between two neutral amino acids or between one neutral and one charged amino acid (in the end state). To first approximation, the electrostatic interaction between two neutral amino acids is a dipole-dipole interaction, which decays as $1/r^3$, where r is the distance between the two dipoles; likewise, the electrostatic interaction between a neutral and a charged amino acid is a charge-dipole interaction, which decays as $1/r^2$. In contrast, charge-charge interactions decay as $1/r$. In this conventional picture, the $(-)$ XaY mutation should be less unfavorable than the (0) XaY one, i.e., $\Delta G_{(-)\mathrm{X}a\mathrm{Y}} < \Delta G_{(0)\mathrm{X}a\mathrm{Y}}$, so $\Delta\Delta G > 0$; we call these interactions repulsive or interfering.

A negative value of $\Delta\Delta G$ indicates a synergistic interaction: it is harder to mutate an acidic amino acid to a neutral one in the presence of a nearby acidic residue than when the neighbor is not acidic. Our results below will show that this synergistic interaction indeed stems from the interactions between the two acidic amino acids.

Practically, to account for the uncertainty in the calculated values of $\Delta\Delta G$, we consider that negligible interactions exist if $|\Delta\Delta G| < 0.5$ kcal$\cdot$mol^{-1}. Significant interfering interactions occur if $\Delta\Delta G > 0.5$ kcal$\cdot$mol^{-1} and significant synergistic interactions exist if $\Delta\Delta G < -0.5$ kcal$\cdot$ mol^{-1}.

We note that other approaches to assess whether neighboring acidic amino acids have synergistic interactions are in principle possible. For example, mutating charged amino acids to their protonated versions should lead to results comparable with ours, because protonating the acid minimally perturbs its LJ interactions with its neighbors. The definition proposed here (Eqs. 2, 3, and 4) has the advantage of being more intuitive because it relies on protonation states realistically accessible at the same pH. Quantitative definitions relying on mutations of charged amino acids to purely hydrophobic ones, however, will likely introduce larger changes also in the LJ interactions between them, making it harder to isolate the electrostatic effect that is thought to be at the source of synergistic interactions.

Free energies of D → N and E → Q mutations in folded proteins

We calculated $\Delta\Delta G$ for selected pairs of acidic residues close to each other (the distance between the a and b carboxylate carbons is < 7 Å at $t = 0$) on the surface of three halophilic proteins: halophilic ferredoxin (PDB: 1DOI), halophilic protein L (PDB: 2KAC), and halophilic dihydrofolate reductase (PDB: 2ITH). These proteins were selected because they differ in net electric charge and size. Protein L has a net charge of $-15e$ but is substantially smaller (only 64 amino acids) than either the halophilic dihydrofolate reductase (162 amino acids) or the halophilic ferredoxin (128 amino acids). The halophilic dihydrofolate reductase is substantially less charged ($-15e$) than the halophilic ferredoxin ($-29e$). Their diverse characteristics thus enable us to draw general conclusions regarding the importance of synergistic interactions between acidic amino acids in halophilic proteins. Given the high computational cost of these calculations, we investigated a smaller set of amino acid pairs in two of the proteins at a low salt concentration ($b_{\mathrm{KCl}} = 0.15$ mol$\cdot$kg^{-1}) and a larger set of pairs in the three proteins at the highest salt concentration ($b_{\mathrm{KCl}} = 2$ mol$\cdot$kg^{-1}), for which the force fields yield reliable results.

Fig. 3 shows the calculated values of $\Delta\Delta G$ in (i). The position of the residues of each pair is shown in (ii). The amino acid pairs and the mutations performed are indicated using condensed notation: e.g., (D,N36) … E41Q indicates that the glutamate in position 41 of the amino acid sequence is mutated to glutamine; the vicinal amino acid is in position 36 and is either an aspartate (when calculating $\Delta G_{(-)\mathrm{E41Q}}$) or an asparagine (when calculating $\Delta G_{(0)\mathrm{E41Q}}$). Fig. 3 *A* and *B* shows values of $\Delta\Delta G$ for the two proteins investigated at low KCl concentration in light blue. Most values of $\Delta\Delta$ G are positive, but vary substantially (between 0.5 and 2 kcal$\cdot$mol^{-1}) depending on the pair of amino acids being investigated. These results are qualitatively in line with the conventional expectation that, at low salt concentration, neighboring acidic amino acids should repel. Synergistic interactions between acidic amino acids at low KCl concentration are only observed for one of the sites ((D,N38) … E41Q of protein L) and are very weak ($\Delta\Delta G = -0.5$ kcal$\cdot$mol^{-1}).

At high KCl concentration, however, the picture that emerges is very different: significant synergistic interactions are observed in 5 out of the 18 pairs of amino acids investigated (Fig. 3 *A–C*), i.e., for those with $\Delta\Delta G < -0.5$ kcal$\cdot$mol^{-1}, as described in the previous subsection. Moreover, synergistic interactions have similar

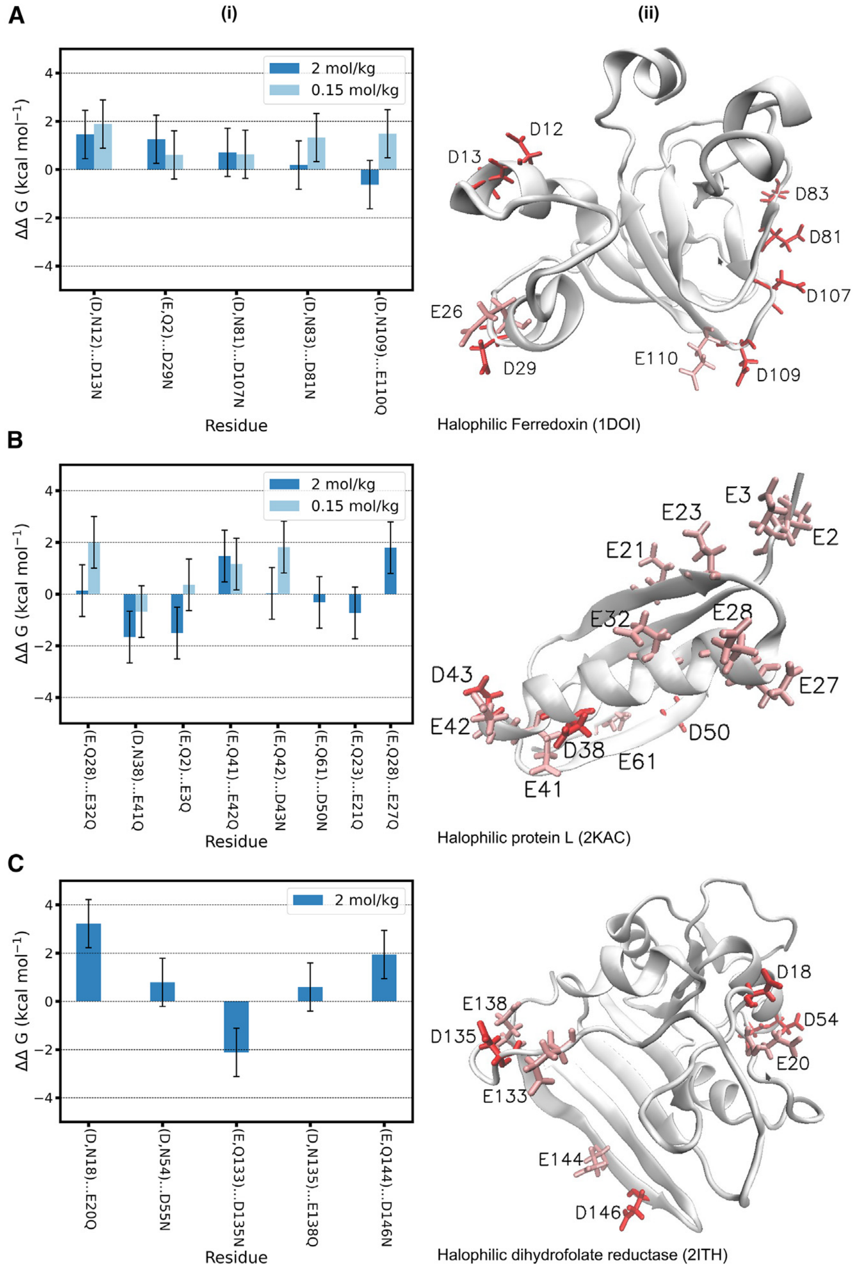

FIGURE 3 Interactions between pairs of acidic amino acids in folded halophilic proteins. (*i*) Change in free energy of mutation ($\Delta\Delta G \pm 1.0$ kcal$\cdot$mol^{-1}; Eq. 4) for selected pairs of amino acids of the indicated proteins. The $\Delta\Delta G$ values are compiled in Tables S4–S6. The error bars are calculated using error propagation, using as input the standard error of the mean of each ΔG value estimated from five independent calculations for one of the protein sites as described in supporting material, section 3. (*ii*) Structure of each protein during the free energy calculation simulations; the acidic amino acids in (*i*) are displayed in red (D, aspartic acid) and in pink (E, glutamic acid). To see this figure in color, go online.

magnitude (-0.5 to -2 kcal$\cdot$mol^{-1}) as the observed repulsive interactions (positive $\Delta\Delta G$ cases) in other amino acid pairs at the same concentration; in other words, the synergistic effect, when present, is of considerable magnitude. In half of the pairs investigated at high salt concentration, we observe interfering (i.e., repulsive) interactions. The values of positive $\Delta\Delta G$ are clearly lower than those at low KCl concentration for the same pairs, reflecting the expected higher electrostatic shielding brought by the more concentrated electrolyte solution. This result illustrates the limitations of mean field descriptions of electrostatic interactions for short distances: mean field calculations predict that the electrostatic interaction between acidic amino acids at $b_{KCl} = 2$ mol$\cdot$kg^{-1} is fully shielded for distances larger than 2 Å, i.e., $\Delta\Delta G$ should be very close to zero for the pairs we selected. In our calculations, full shielding is only observed for 3 of the 18 pairs tested.

The results in Fig. 3 demonstrate that a synergistic interaction between neighboring acidic amino acids is possible, and in fact occurs frequently at high salt concentration: in our data set, we observe it in $\approx 1/3$ of the amino acid pairs tested. The solvent-mediated (i.e., water- and ion-mediated) interaction between the amino acids in these pairs thus contributes to the stabilization of the folded protein structure. Moreover, for $1/6$ of the amino acid pairs tested at high salt concentration, the interaction between the neighboring

amino acids was reduced to zero: those pairs neither stabilize nor destabilize the folded protein structure. For half of the pairs investigated at high salt concentration, however, we observe positive values of $\Delta\Delta G$. Overall, our results suggest that the ion-solvent stabilization effect exists. The fact that a synergistic interaction is more frequent at high salt concentration confirms that it is mediated by the ions and water. Nevertheless, this effect contributes only to a limited extent to the stabilization of the folded structure of halophilic proteins, at least at $b_{KCl} = 2\ \text{mol}\cdot\text{kg}^{-1}$. It is possible that the ion-solvent stabilization effect becomes more intense and more likely at even higher salt concentrations, but at present this possibility cannot be investigated using molecular simulations because force fields that remain accurate up to the solubility limit of the salt do not exist.

Free energies of D → N and E → Q mutations in the unfolded halophilic protein L

We next tested whether synergistic interactions between acidic amino acids in unfolded proteins are possible: we calculated $\Delta\Delta G$ for multiple pairs of spatially close acidic amino acids belonging to an unfolded structure of halophilic protein L, and compared the frequency of occurrence and magnitude of synergistic effects relative with those observed for the same protein in the folded state. Protein L was selected because, of the three proteins investigated in the folded state, it had the most frequent synergistic interactions. The unfolded configuration was obtained from a replica exchange molecular dynamics (REMD) simulation, as described in supporting material, section 6. The REMD simulation could not sample the full ensemble of unfolded configurations; rather, it sampled a subset of configurations where the protein is denatured but collapsed. For our purpose this behavior is not an impediment because synergistic interactions, if present in the unfolded state, should be more likely when pairs of acidic amino acids are sufficiently close. Theseus (46) was used to find the configuration with the structure most similar to the average structure in the REMD trajectory at 298 K. This configuration is shown in Fig. 4. The backbone of the structure was restrained during the free energy calculations, similarly to the calculations done for the folded proteins.

Fig. 4 *A* shows the $\Delta\Delta G$ values for the denatured structure of protein L at $b_{KCl} = 2\ \text{mol}\cdot\text{kg}^{-1}$. Surprisingly, almost all of the pairs of amino acids show synergistic interactions, and the value of $\Delta\Delta G$ is more negative in some cases than the synergistic cases in the folded structure of the same protein. These results do not support the scenario that synergistic effects between pairs of amino acids are enabled by specific amino acid configurations exclusive to the folded protein structure (44). On the contrary, it seems that pairs of acidic residues assume relative distances and conformations leading to synergistic interactions very easily in denatured conformations of halophilic protein L.

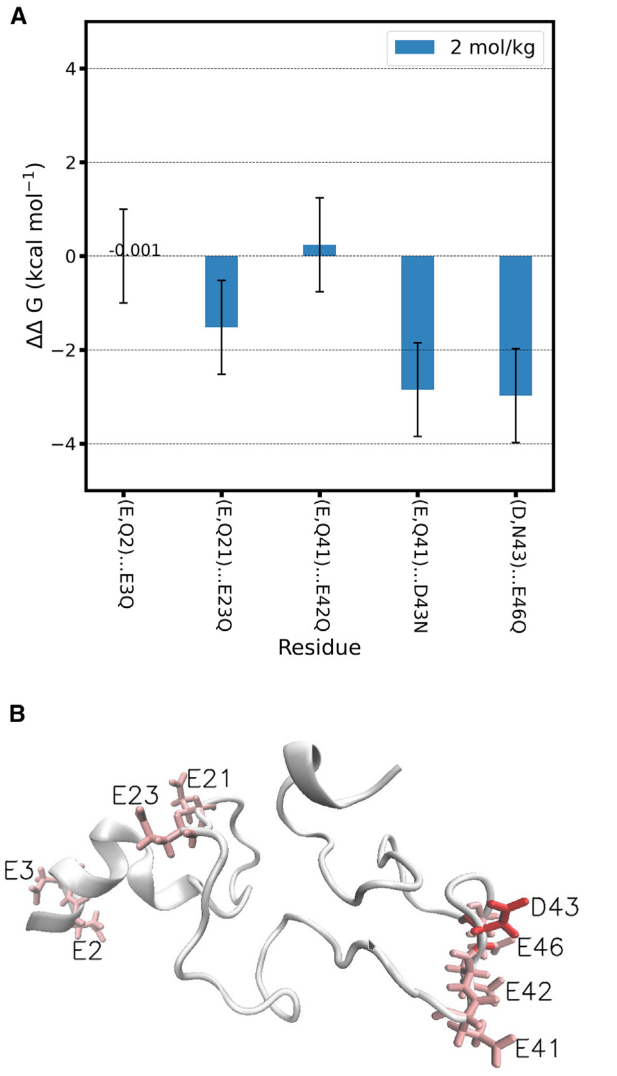

FIGURE 4 Interactions between pairs of acidic amino acids in the unfolded halophilic protein L at $b_{KCl} = 2\ \text{mol}\cdot\text{kg}^{-1}$. (*A*) Change in free energy of mutation ($\Delta\Delta G \pm 1.0\ \text{kcal}\cdot\text{mol}^{-1}$; Eq. 4) for selected pairs of amino acids. The $\Delta\Delta G$ values are compiled in Table S7. The error bars are calculated using error propagation, using as input the standard error of the mean of each ΔG value estimated from five independent calculations for one of the protein sites as described in supporting material, section 3. (*B*) Unfolded conformation of protein L used in the free energy calculations; the acidic amino acids in (*A*) are displayed in red (D, aspartic acid) and in pink (E, glutamic acid). To see this figure in color, go online.

Mechanism behind the synergistic effect

Negative values of $\Delta\Delta G$ result from synergistic electrostatic interactions between acidic amino acids

To further understand the mechanism leading to the synergistic effect, we examined the three contributions to $\Delta\Delta G$ along the mutation path. The free energy associated with a generic mutation X*a*Y is calculated in three steps

$$\Delta G_{XaY} = \Delta G_{decharge} + \Delta G_{vdW} + \Delta G_{charge} \qquad (5)$$

following the thermodynamic cycle shown in Eq. 1. The term $\Delta G_{\text{decharge}}$ is the free energy change associated with decharging amino acid X, i.e., setting all atomic charges of this residue to zero. The term ΔG_{vdW} corresponds to mutating the decharged residue X^0 into the decharged residue Y^0; this term includes only the contributions of the changes in the LJ potentials of the residue to the interactions with its environment and is often termed the vdW contribution. The final term, ΔG_{charge}, is the free energy change associated with reinstating the atomic charges of amino acid Y. The values of $\Delta\Delta G$ can thus be decomposed into decharging, vdW, and charging contributions as:

$$\Delta\Delta G_{\text{decharge}} = \Delta G_{(0)\text{decharge}} - \Delta G_{(-)\text{decharge}} \tag{6}$$

$$\Delta\Delta G_{\text{vdW}} = \Delta G_{(0)\text{vdW}} - \Delta G_{(-)\text{vdW}} \tag{7}$$

$$\Delta\Delta G_{\text{charge}} = \Delta G_{(0)\text{charge}} - \Delta G_{(-)\text{charge}} \tag{8}$$

These components are shown in Fig. 5 for the folded halophilic protein L at $b_{\text{KCl}} = 2\ \text{mol}\cdot\text{kg}^{-1}$. The $\Delta\Delta G_{\text{vdW}}$ component takes both positive and negative values, but its absolute value is always below $\approx 0.5\ \text{kcal}\cdot\text{mol}^{-1}$. The vdW component of the mutation free energy is thus essentially independent of the identity of the vicinal amino acid. The sign of $\Delta\Delta G$ is thus determined by the balance between the charging and the decharging steps. The charging step, which introduces the atomic charges in the final (neutral) amino acid, predominantly has a positive contribution to $\Delta\Delta G$. In contrast, the decharging step, which removes the atomic charges in the initial (negative) amino acid, negatively contributes to $\Delta\Delta G$.

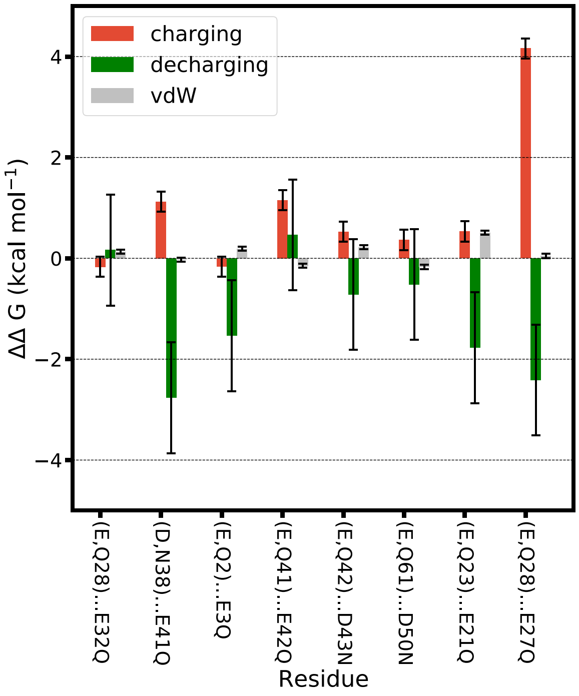

FIGURE 5 Components of $\Delta\Delta G$ for the indicated pairs of amino acids of the halophilic protein L at $b_{\text{KCl}} = 2\ \text{mol}\cdot\text{kg}^{-1}$. $\Delta\Delta G_{\text{charge}} \pm 0.2\ \text{kcal}\cdot\text{mol}^{-1}$, $\Delta\Delta G_{\text{decharge}} \pm 1.1\ \text{kcal}\cdot\text{mol}^{-1}$, and $\Delta\Delta G_{\text{vdW}} \pm 0.04\ \text{kcal}\cdot\text{mol}^{-1}$ (Eqs. 6, 7, and 8). The error bars are standard errors of the mean, estimated from 5 independent calculations for one of the protein sites as described in supporting material, section 3. The sum of the components for each amino acid pair equals the values shown in Fig. 3 *Bi*). To see this figure in color, go online.

This result confirms that a negative $\Delta\Delta G$ is associated with the decharging step. A negative value of $\Delta\Delta G_{\text{decharge}}$ could arise either from 1) unexpected *stabilizing* electrostatic interactions between the two vicinal acidic amino acids, or between 2) *destabilizing* electrostatic interactions between vicinal acidic and neutral amino acids. As discussed in detail in supporting material, section 5, the first possibility is the one at play here.

Synergistic interactions are not observed in minimal systems of acidic amino acids

Do synergistic interactions between acidic amino acids occur in minimal systems, i.e., outside of a protein environment? Does the synergistic effect occur for particular distances or relative orientations? To answer these questions, we characterized the thermodynamics of the interaction of minimal systems mimicking the side chains of aspartate and of asparagine by calculating the PMF as a function of their distance. In the minimal systems, the carbon corresponding to the C_α in the full amino acid now forms a CH_3 group; that carbon and the hydrogen atoms directly bonded to it have modified charges, so the nominal charge of the side chain is the same as that of the original amino acid. For conciseness, we refer to the amino acid models as side chains, and by the names (aspartate or asparagine) and one-letter code (D or N) of the corresponding amino acids.

Fig. 6 *A* shows the PMF as a function of $C_\alpha \cdots C_\alpha$ distance (ξ) for the side chain pairs D-D, N-N, and D-N, at $b_{\text{KCl}} = 0.15\ \text{mol}\cdot\text{kg}^{-1}$ and at $b_{\text{KCl}} = 2\ \text{mol}\cdot\text{kg}^{-1}$, for the case where the side chains are parallel to each other and perpendicular to ξ. The curves are shifted along the y axis for ease of viewing, so the absolute values of $\Delta G(\xi)$ have no physical meaning. Differences between the three curves at each salt concentration, or within each curve, are physically meaningful. The D-N and N-N interactions at large distances can be assumed to be zero; for this reason, the curves for the D-N and N-N pairs at each salt concentration were shifted along the y axis to coincide at $\xi = 15$ Å. The interaction energy between the D-D pair at $\xi = 15$ Å at each salt concentration was estimated analytically as the Coulomb interaction between two point charges. Each D-D curve was shifted so that the difference $\Delta G_{\text{D-D}}(15\ \text{Å}) - \Delta G_{\text{N-N}}(15\ \text{Å})$ reproduces the analytical estimate for the respective salt concentration. At both concentrations, the maximum repulsion experienced by two negative side chains occurs at $\xi \approx 6$ Å, and is weak ($< 1\ \text{kcal}\cdot\text{mol}^{-1}$). The D-N and N-N pairs have negligible interaction energy down to $\xi \approx 5$ Å, as expected because

charge-dipole and dipole-dipole interactions are short range and are screened at the salt concentrations considered. Below $\xi \approx 4.5$ Å, the CH_3 and CH_2 groups of the side chains are very close, whereas the terminal atoms point away from each other, as the representative configuration shown in Fig. S10 illustrates. As a result of this preferential configuration, attractive LJ interactions dominate over electrostatics, giving rise to a minimum in the PMF in all three cases (calculations shown in supporting material, section 7). Results (not shown) for other relative orientations of the side chains have the same qualitative features; the main differences are observed in the position and height of the repulsive maximum of the D-D pair. PMF curves calculated based on the distance between the terminal (i.e., the γ) carbons of the side chain show similar characteristics, as illustrated in Fig. S11 for the D-D pair.

To assess whether a synergistic effect can arise from side-chain pairs, we computed the quantity $\Delta\Delta G(\xi)$ from the PMF curves:

$$\begin{aligned}\Delta\Delta G(\xi) &= \left[\Delta G(\xi)_{\mathrm{N-N}} - \Delta G(\xi)_{\mathrm{N-D}}\right] - \left[\Delta G(\xi)_{\mathrm{D-N}} - \Delta G(\xi)_{\mathrm{D-D}}\right] \\ &= \Delta G(\xi)_{\mathrm{D-D}} + \Delta G(\xi)_{\mathrm{N-N}} - 2 \times \Delta G(\xi)_{\mathrm{D-N}}\end{aligned} \tag{9}$$

The subscripts indicate the pair of side chains for each PMF. The quantity $\Delta\Delta G(\xi)$ is analogous to the $\Delta\Delta G$ calculated for pairs of acidic amino acids on proteins (Figs. 3 and 4) but gives insight into cooperativity as a function of the distance between the side chains. Fig. 6 *B* shows $\Delta\Delta G(\xi)$ for both salt concentrations, for side chains at different relative orientations. The value and position of the maxima of each curve depend strongly on relative orientation. Nevertheless, this quantity never assumes negative values, indicating that a synergistic effect does not occur in this two-body system. Could it be that a synergistic effect arises in minimal systems with more side chains? To answer this question we performed an analogous PMF study for a system of three side chains. The results, presented in supporting material, section 8, indicate that a synergistic effect does not occur in that system either.

The absence of the synergistic effect in the two-body and three-body systems suggests that synergistic interactions between acidic amino acids do not result solely from water- and salt-mediated interactions between the acidic amino acids, but are also enabled by particular local protein environments.

The synergistic effect does not require particular amino acid orientations

The large variation observed in the values of $\Delta\Delta G$ ($-3 < \Delta\Delta G$ (kcal·mol^{-1}) $< +3$) shown in Figs. 3 and 4 indicates that solvent-mediated interactions (synergistic or repulsive) between charged amino acids at high salt concentration are strongly affected by local protein composition and local structure. Obvious correlations between the sign or magnitude of $\Delta\Delta G$ and the secondary structural motifs in which the amino acids of the pair are located or the identity of other neighboring amino acids are not present. Moreover, in our data set the frequency of synergistic interactions differs strongly between proteins: they occur more frequently in protein L than for the other two proteins. Because the data set is small, it is at present unclear whether these differences between the proteins are significant. Clarifying these aspects will require their systematic study through a substantially larger data set of mutation free energy calculations, which is beyond what is currently possible.

We have observed a substantial synergistic effect in a denatured configuration of the halophilic protein L. This observation contradicts the claim of the lack of necessary preorientation for cation-acidic residue interaction in the unfolded structure. To gain further insight into the connection between the relative orientation of amino acids and their synergistic or repulsive interactions, we simulated the folded halophilic protein L, at $b_{\mathrm{KCl}} = 2$ mol·kg^{-1}, to obtain a long, continuous trajectory (see supporting material, section 2.2 for simulation details). We analyzed this trajectory to determine whether characteristic configurations of acidic amino acids differ between pairs of amino acids showing synergistic versus interfering effects. Fig. 7 compares the histograms of distances between the carboxylate carbons of the amino acid pairs for which $\Delta\Delta G$ was calculated (Fig. 3 *Bi*). The distribution of distances differs substantially between pairs, and a correlation between the characteristics of the distribution and the magnitude or sign of $\Delta\Delta G$ cannot be discerned. The distribution of distances for pairs of amino acids showing synergistic interactions (in *blue*) can be narrow and associated with short distances (5–6 Å) corresponding to contact ion pair configurations, or can be quite broad and include distances that correspond to solvent-shared ion pair configurations. The distributions of distances for pairs of amino acids showing interfering interactions (in *red*) are equally broad and span the same distances. These results do not support the claim that the synergistic effect, when it exists, arises from amino acids with particular and well-defined relative orientations.

Enthalpic/entropic origins of the synergistic effect

To assess whether the synergistic effect observed for some of the protein sites could originate from stronger hydrogen bonds between water and the carboxylate groups of the

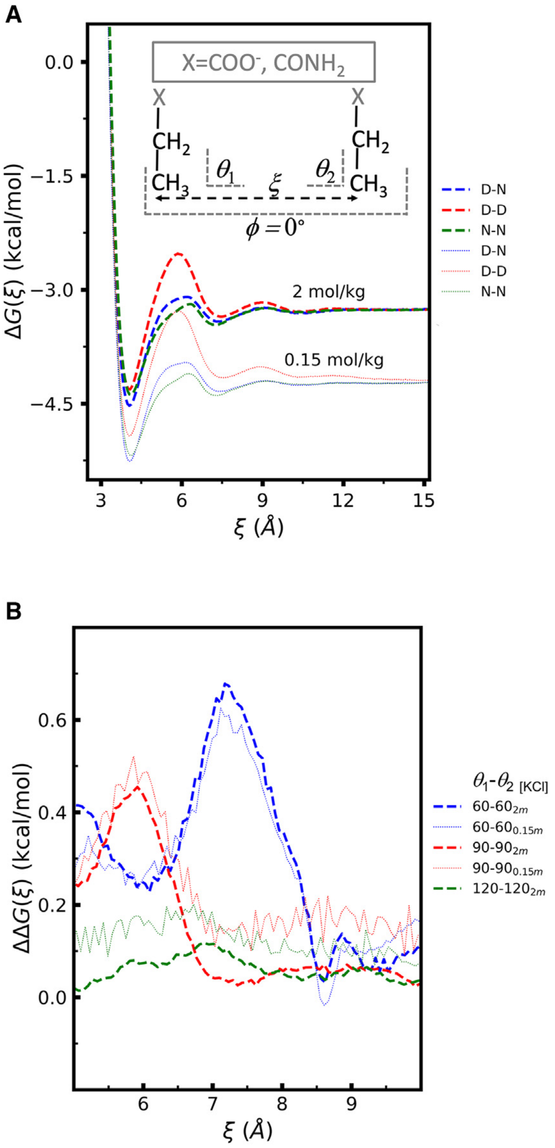

FIGURE 6 Potential of mean force as a function of $C_{\alpha} \cdots C_{\alpha}$ distance, for different orientations of pairs of side chain mimics, at $b_{KCl} = 0.15$ mol$\cdot$ kg^{-1} (0.15m) and $b_{KCl} = 2$ mol$\cdot$kg^{-1} (2m). (*A*) PMF for the indicated pairs of amino acid side chains, for parallel side chains (restraint angles $\theta_1 = \theta_2 = 90^{\circ}$; $\varphi = 0^{\circ}$). The ideal gas entropic term ($-2k_BT\ln(\xi)$) is not included in the curves. (*B*) $\Delta\Delta G(\xi)$ (Eq. 9) for different relative orientations (θ_1 and θ_2; $\varphi = 0^{\circ}$) of the side chains, at $b_{KCl} = 0.15$ mol$\cdot$ kg^{-1} (0.15m) or $b_{KCl} = 2$ mol$\cdot$kg^{-1} (2m). To see this figure in color, go online.

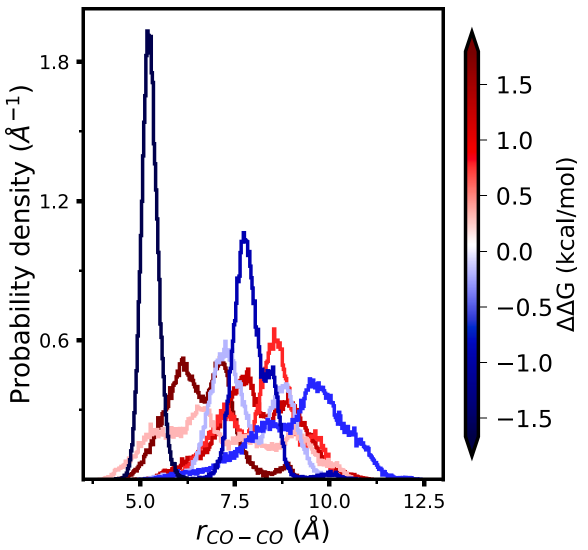

FIGURE 7 Histogram of the distances between carboxylate carbons of the folded protein L from an MD simulation at $b_{KCl} = 2$ mol$\cdot$kg^{-1}, for the amino acid pairs for which $\Delta\Delta G$ was calculated (Figure 3 *Bi*). The color scale shows the value of $\Delta\Delta G$, with blue used for pairs showing a synergistic interaction and red used for pairs with an interfering interaction. To see this figure in color, go online.

acidic amino acids in those cases, we quantified the geometry of those hydrogen bonds separately for the synergistic and the interfering pairs of amino acids of protein L from the same simulation on which Fig. 7 is based. Fig. 8 *A* shows the normalized histogram ($p_{syn}(d, \cos\theta)$) of the distance (d) between the water and the carboxylate oxygens versus the cosine of the angle θ for water molecules near carboxylate groups of the amino acid pairs showing a synergistic effect. The hydrogen bond strength increases for shorter distances and for cosine values close to -1, i.e., for θ near 180°. Water preferentially donates strong hydrogen bonds to the carboxylate groups, as indicated by the short values of d and by cosine values near -1. These results are consistent with previous work, which indicates that carboxylates accept strong hydrogen bonds from water (47). In Fig. 8 *B* we show the difference between the histograms obtained for the interfering and for the synergistic residues ($p_{syn}(d, \cos\theta) - p_{int}(d, \cos\theta)$). This difference shows that the synergistic sites accept stronger hydrogen bonds and accept fewer weaker hydrogen bonds than the interfering sites. This result confirms that the synergistic effect has an enthalpic contribution arising from solute-water interactions.

We found no mechanistic correlation between the radial distribution function of the cation and the presence or absence of synergistic interactions. Further assessing the contribution of the cation toward the difference in hydrogen bond strength between water and the carboxylate groups, and assessing whether entropy also contributes to the synergistic effect, will require the quantification of local entropy and enthalpy at the interfering and synergistic protein sites. At present, algorithms to quantify local enthalpic and entropic contributions to solvation free energies (48,49) can only be applied to solutes in pure water, so these aspects cannot be investigated in our system.

CONCLUSIONS

We used molecular simulations to investigate whether synergistic—rather than the expected interfering—interactions between neighboring acidic amino acids exist and contribute to the stabilization of folded halophilic proteins, as originally

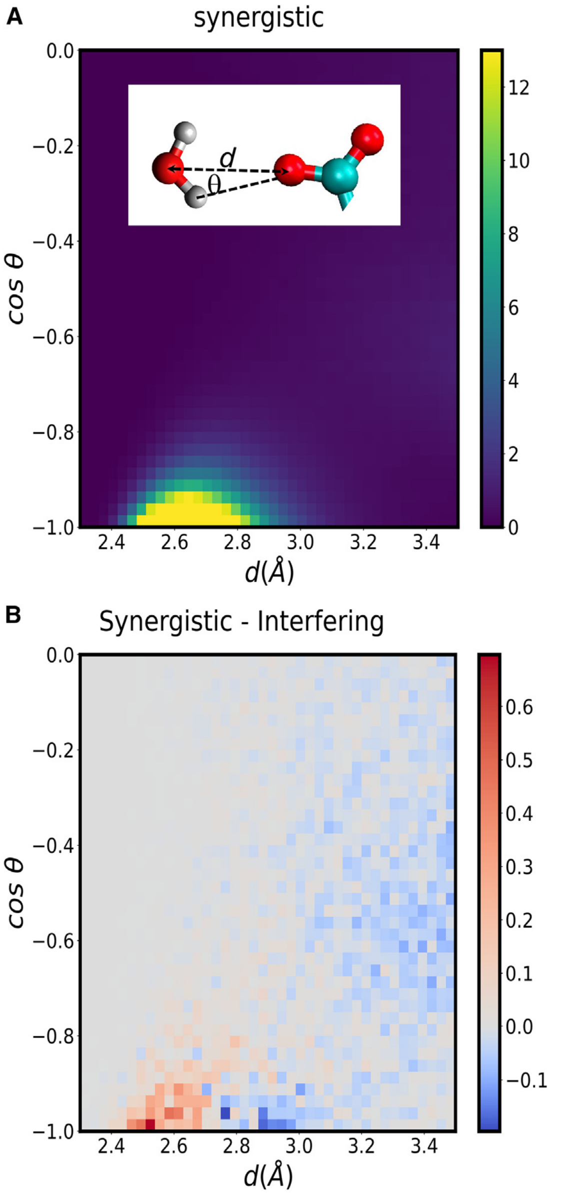

FIGURE 8 Geometric features of hydrogen bonds donated by water molecules to the carboxylate groups of acidic amino acids in protein L. (*A*) Histogram, $p_{\mathrm{syn}}(d, \cos\theta)$, of the distance and angle characterizing hydrogen bonds accepted by the residues of synergistic pairs. The inset shows a water molecule near a carboxylate group, and illustrates the distance and angle used for the histograms. (*B*) Difference, $p_{\mathrm{syn}}(d, \cos\theta) - p_{\mathrm{int}}(d, \cos\theta)$, between the histograms characterizing hydrogen bonds accepted by residues of synergistic and interfering pairs. To see this figure in color, go online.

proposed by Zaccai et al. (24) and supported by NMR studies of halophilic proteins (44). Our results partially support this hypothesis, clarify its mechanism, and point to further studies necessary to fully understand the origin of synergistic interactions.

Quantitative investigations of this hypothesis have been hampered by the lack of an operational definition of synergistic interactions. Our first step was thus to address this difficulty: we quantify the interactions between neighboring amino acids as a difference, $\Delta\Delta G$, of two mutation free energies, which can be calculated with standard molecular dynamics simulation packages.

Our $\Delta\Delta G$ calculations show that pairs of neighboring acidic amino acids at the surface of proteins in multimolar KCl solutions may indeed interact synergistically. Synergistic interactions are frequent at multimolar KCl concentration but very infrequent at low KCl concentration, suggesting that they have an electrostatic origin. Decomposing $\Delta\Delta G$ into the decharging, vdW, and charging components confirms that synergistic interactions are associated with the decharging step, i.e., with solvent-mediated electrostatic interactions between two acidic amino acids. Hydrogen bond analysis confirms that acidic amino acids with synergistic interactions accept stronger hydrogen bonds from water more often than those with interfering interactions. Further work is necessary to investigate whether synergistic interactions have also an entropic contribution. We find that synergistic interactions do not occur in minimal systems mimicking amino acid side chains, indicating that the protein environment influences the solvent- and ion-mediated interactions between acidic amino acids and contributes to the occurrence of synergistic interactions between them. However, obvious correlations between the secondary structural motif to which the amino acids belong or their spatially close neighboring amino acids and their synergistic/interfering interactions do not exist. Further work—e.g., investigating whether the protein environment creates electric fields of favorable strength and direction at synergistic but not interfering sites—is necessary to fully clarify the mechanism by which the protein environment allows synergistic interactions to occur in some sites.

Our results support the existence of synergistic interactions between acidic amino acids proposed by Zaccai et al. (24), but do not support their proposed mechanism that synergistic interactions are enabled by specific amino acid configurations exclusive to folded halophilic proteins. Instead, the simulations show that synergistic interactions are not associated with specific or rigid amino acid configurations, and exist also in unfolded conformations. According to Zaccai et al., specific amino acid configurations would lead to unusually slow dynamics of the water of hydration because the anchoring of the acidic residues in the protein would limit the number of available configurations and would slow down the hydrogen bond dynamics. Our previous simulations do not support that scenario: we found that the translational dynamics of water of hydration of halophilic proteins is very similar to that of mesophilic ones (20).

Synergistic interactions should nevertheless stabilize folded relative to unfolded configurations, as initially proposed, because the ensemble of unfolded configurations has many more configurations than the folded one. The configurations for which synergistic interactions occur (where the acidic amino acids are spatially close) are only a small

subset of the total unfolded ensemble, whereas they dominate the ensemble of folded configurations. Therefore, the stabilizing contribution of synergistic interaction to the unfolded ensemble should be smaller than for the folded one. We emphasize that it remains unclear whether synergistic interactions are the main mechanism by which acidic amino acids stabilize the folded structure of halophilic proteins at high salt concentration. It is also possible that nonsynergistic interactions involving acidic amino acids, solvent, and ions lead to different solvation of folded versus unfolded configurations, and that these differences stabilize the folded state (50). Future studies in our group will investigate this possibility.

SUPPORTING MATERIAL

Supporting material can be found online at https://doi.org/10.1016/j.bpj.2023.05.011.

AUTHOR CONTRIBUTIONS

A.V.V. designed and guided the research. H.G.D. performed the research. Both authors wrote the manuscript.

ACKNOWLEDGMENTS

This work was funded by the Deutsche Forschungsgemeinschaft (DFG, German Research Foundation) under grant no. 639429 and under Germany's Excellence Strategy – EXC 2033 – 390677874 – RESOLV.

DECLARATION OF INTERESTS

The authors declare no competing interests.

REFERENCES

1. Lanyi, J. K. 1974. Salt-dependent properties of proteins from extremely halophilic bacteria. *Bacteriol. Rev.* 38:272–290.
2. Deole, R., J. Challacombe, …, W. D. Hoff. 2013. An extremely halophilic proteobacterium combines a highly acidic proteome with a low cytoplasmic potassium content. *J. Biol. Chem.* 288:581–588.
3. van der Wielen, P. W. J. J., H. Bolhuis; …, BioDeep Scientific Party. 2005. The enigma of prokaryotic life in deep hypersaline anoxic basins. *Science.* 307:121–123.
4. Graziano, G., and A. Merlino. 2014. Molecular bases of protein halotolerance. *Biochim. Biophys. Acta.* 1844:850–858.
5. Gunde-Cimermana, N., P. Zalarb, …, A. Plemenitaš. 2000. Hypersaline waters in salterns - natural ecological niches for halophilic black yeasts. *FEMS Microbiol. Ecol.* 32:235–240.
6. Kennedy, S. P., W. V. Ng, …, S. DasSarma. 2001. Understanding the adaptation of Halobacterium species NRC-1 to its extreme environment through computational analysis of its genome sequence. *Genome Res.* 11:1641–1650.
7. Paul, S., S. K. Bag, …, C. Dutta. 2008. Molecular signature of hypersaline adaptation: insights from genome and proteome composition of halophilic prokaryotes. *Genome Biol.* 9:R70.
8. Siglioccolo, A., A. Paiardini, …, S. Pascarella. 2011. Structural adaptation of extreme halophilic proteins through decrease of conserved hydrophobic contact surface. *BMC Struct. Biol.* 11:50.
9. Tessier, P. M., and A. M. Lenhoff. 2003. Measurements of protein self-association as a guide to crystallization. *Curr. Opin. Biotechnol.* 14:512–516.
10. Dumetz, A. C., A. M. Snellinger-O'Brien, …, A. M. Lenhoff. 2007. Patterns of protein-protein interactions in salt solutions and implications for protein crystallization. *Protein Sci.* 16:1867–1877.
11. Guo, B., S. Kao, …, W. William Wilson. 1999. Correlation of second virial coefficients and solubilities useful in protein crystal growth. *J. Cryst. Growth.* 196:424–433.
12. Reed, C. J., S. Bushnell, and C. Evilia. 2014. Circular dichroism and fluorescence spectroscopy of cysteinyl-tRNA synthetase from Halobacterium salinarum ssp. NRC-1 demonstrates that group I cations are particularly effective in providing structure and stability to this halophilic protein. *PLoS One.* 9, e89452.
13. Rode, B. M. 1999. Peptides and the origin of life. *Peptides.* 20:773–786.
14. Longo, L. M., J. Lee, and M. Blaber. 2013. Simplified protein design biased for prebiotic amino acids yields a foldable, halophilic protein. *Proc. Natl. Acad. Sci. USA.* 110:2135–2139.
15. Longo, L. M., and M. Blaber. 2014. Prebiotic protein design supports a halophile origin of foldable proteins. *Front. Microbiol.* 4:418.
16. Rossino, G., M. S. Robescu, …, S. Collina. 2022. Biocatalysis: a smart and green tool for the preparation of chiral drugs. *Chirality.* 34:1403–1418.
17. Bangaru, A., K. A. Sree, …, C. N. Reddy. 2022. Role of Enzymes in Biofuel Production: Recent Developments and Challenges. Springer Nature Singapore, pp. 81–112.
18. Madern, D., and G. Zaccai. 2004. Molecular adaptation: the malate dehydrogenase from the extreme halophilic bacterium Salinibacter ruber behaves like a non-halophilic protein. *Biochimie.* 86:295–303.
19. Nath, A. 2016. Insights into the sequence parameters for halophilic adaptation. *Amino Acids.* 48:751–762.
20. Geraili Daronkola, H., and A. Vila Verde. 2021. Proteins maintain hydration at high [KCl] concentration regardless of content in acidic amino acids. *Biophys. J.* 120:2746–2762.
21. Elcock, A. H., and J. A. McCammon. 1998. Electrostatic contributions to the stability of halophilic proteins. *J. Mol. Biol.* 280:731–748.
22. Kuntz, I. D. 1971. Hydration of macromolecules. III. Hydration of polypeptides. *J. Am. Chem. Soc.* 93:514–516.
23. Frolow, F., M. Harel, …, M. Shoham. 1996. Insights into protein adaptation to a saturated salt environment from the crystal structure of a halophilic 2Fe-2S ferredoxin. *Nat. Struct. Biol.* 3:452–458.
24. Zaccai, G., F. Cendrin, …, H. Eisenberg. 1989. Stabilization of halophilic malate dehydrogenase. *J. Mol. Biol.* 208:491–500.
25. Pundak, S., and H. Eisenberg. 1981. Structure and activity of malate dehydrogenase from the extreme halophilic bacteria of the Dead Sea: 1. Conformation and interaction with water and salt between 5 M and 1 M NaCl concentration. *Eur. J. Biochem.* 118:463–470.
26. Madern, D., C. Ebel, and G. Zaccai. 2000. Halophilic adaptation of enzymes. *Extremophiles.* 4:91–98.
27. Irimia, A., C. Ebel, …, F. M. D. Vellieux. 2003. The oligomeric states of Haloarcula marismortui malate dehydrogenase are modulated by solvent components as shown by crystallographic and biochemical studies. *J. Mol. Biol.* 326:859–873.
28. Calmettes, P., H. Eisenberg, and G. Zaccai. 1987. Structure of halophilic malate dehydrogenase in multimolar KCl solutions from neutron scattering and ultracentrifugation. *Biophys. Chem.* 26:279–290.
29. Jasnin, M., A. Stadler, …, G. Zaccai. 2010. Specific cellular water dynamics observed in vivo by neutron scattering and NMR. *Phys. Chem. Chem. Phys.* 12:10154–10160.

30. Qvist, J., G. Ortega, …, B. Halle. 2012. Hydration dynamics of a halophilic protein in folded and unfolded states. *J. Phys. Chem. B.* 116:3436–3444.

31. Kashefolgheta, S., and A. Vila Verde. 2017. Developing force fields when experimental data is sparse: AMBER/GAFF-compatible parameters for inorganic and alkyl oxoanions. *Phys. Chem. Chem. Phys.* 19:20593–20607.

32. Jorgensen, W. L., J. Chandrasekhar, …, M. L. Klein. 1983. Comparison of simple potential functions for simulating liquid water. *J. Chem. Phys.* 79:926–935.

33. Cornell, W. D., P. Cieplak, …, P. A. Kollman. 1995. A second generation force field for the simulation of proteins, nucleic acids, and organic molecules. *J. Am. Chem. Soc.* 117:5179–5197.

34. Maier, J. A., C. Martinez, …, C. Simmerling. 2015. ff14SB: improving the accuracy of protein side chain and backbone parameters from ff99SB. *J. Chem. Theory Comput.* 11:3696–3713.

35. Joung, I. S., and T. E. Cheatham. 2008. Determination of alkali and halide monovalent ion parameters for use in explicitly solvated biomolecular simulations. *J. Phys. Chem. B.* 112:9020–9041.

36. Case, D., I. Ben-Shalom, …, P. A. Kollman. 2018. Amber 18. University of California.

37. Tadeo, X., B. López-Méndez, …, O. Millet. 2009. Structural basis for the aminoacid composition of proteins from halophilic archea. *PLoS Biol.* 7, e1000257.

38. Binbuga, B., A. F. B. Boroujerdi, and J. K. Young. 2007. Structure in an extreme environment: NMR at high salt. *Protein Sci.* 16:1783–1787.

39. Pettersen, E. F., T. D. Goddard, …, T. E. Ferrin. 2004. UCSF Chimera - a visualization system for exploratory research and analysis. *J. Comput. Chem.* 25:1605–1612.

40. Klimovich, P. V., M. R. Shirts, and D. L. Mobley. 2015. Guidelines for the analysis of free energy calculations. *J. Comput. Aided Mol. Des.* 29:397–411, HHS Public Access.

41. Berendsen, H. J. C., D. van der Spoel, and R. van Drunen. 1995. GROMACS: a message-passing parallel molecular dynamics implementation. *Comput. Phys. Commun.* 91:43–56.

42. Van Der Spoel, D., E. Lindahl, …, H. J. C. Berendsen. 2005. GROMACS: fast, flexible, and free. *J. Comput. Chem.* 26:1701–1718.

43. Kumar, S., J. M. Rosenberg, …, P. A. Kollman. 1992. The weighted histogram analysis method for free-energy calculations on biomolecules. I. The method. *J. Comput. Chem.* 13:1011–1021.

44. Ortega, G., T. Diercks, and O. Millet. 2015. Halophilic protein adaptation results from synergistic residue-ion interactions in the folded and unfolded states. *Chem. Biol.* 22:1597–1607.

45. Deng, Y., and B. Roux. 2004. Hydration of amino acid side chains: nonpolar and electrostatic contributions calculated from staged molecular dynamics free energy simulations with explicit water molecules. *J. Phys. Chem. B.* 108:16567–16576.

46. Theobald, D. L., and D. S. Wuttke. 2008. Accurate structural correlations from maximum likelihood superpositions. *PLoS Comput. Biol.* 4:43.

47. Vinogradov, S. N., and R. H. Linnell. 1971. Hydrogen Bonding. Van Nostrand Reinhold.

48. Lazaridis, T. 1998. Inhomogeneous fluid approach to solvation thermodynamics. 1. Theory. *J. Phys. Chem. B.* 102:3531–3541.

49. Heyden, M. 2019. Disassembling solvation free energies into local contributions—toward a microscopic understanding of solvation processes. *Wiley Interdiscip. Rev. Comput. Mol. Sci.* 9:e1390.

50. Smiatek, J. 2017. Aqueous ionic liquids and their effects on protein structures: an overview on recent theoretical and experimental results. *J. Phys. Condens. Matter.* 29, 233001.

Biophysical Journal, Volume *122*

Supplemental information

Prevalence and mechanism of synergistic carboxylate-cation-water interactions in halophilic proteins

Hosein Geraili Daronkola and Ana Vila Verde

S1 Molecular formula of amino acids

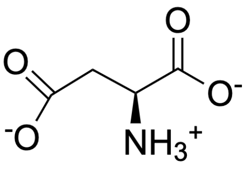

(a) Aspartate (D)

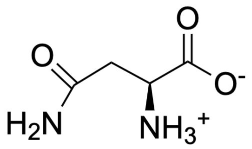

(b) Asparagine (N)

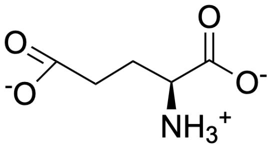

(c) Glutamate (E)

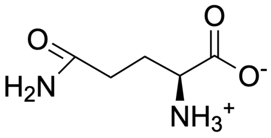

(d) Glutamine (Q)

Fig. S1 Molecular formula of each amino acid in its zwitterionic form.

S2 Simulation details

S2.1 Free energy calculations

The free energy values reported in the main text were obtained from free energy calculations using optimized simulation parameters described in this section; section S3 describes how the optimized parameters were determined.

For step (2) in Scheme 1 in the main text, 34 λ values of 0.00 (X_a^0), 0.02, 0.04, 0.06, 0.08, 0.10, 0.12, 0.14, 0.16, 0.18, 0.20, 0.22, 0.24, 0.26, 0.28, 0.30, 0.35, 0.40, 0.45, 0.50, 0.55, 0.60, 0.65, 0.70, 0.75, 0.80, 0.85, 0.88, 0.90, 0.92, 0.94, 0.96, 0.98, 1.00 (Y_a^0) were used. The value returned by this calculation is ΔG_{vdW}. For practical reasons, in the case of steps (1) and (3), the thermodynamic steps actually calculated were the reverse of those shown in Scheme 1. For step (1), 37 λ values of 0.00 (X_a^0), 0.05, 0.10, 0.15, 0.20, 0.25, 0.30, 0.35, 0.40, 0.45, 0.50, 0.55, 0.60, 0.61, 0.62, 0.63, 0.64, 0.65, 0.66, 0.67, 0.68, 0.69, 0.70, 0.71, 0.72, 0.73, 0.74, 0.75, 0.76, 0.77, 0.78, 0.79, 0.80, 0.85, 0.90, 0.95, 1.00 (X_a) were used. The value returned by this calculation was $-\Delta G_{\mathrm{decharge}}$. For step (3), 24 λ values of 0.00 (Y_a), 0.02, 0.04, 0.06, 0.08, 0.10, 0.15, 0.20, 0.25, 0.30, 0.35, 0.40, 0.45, 0.50, 0.55, 0.60, 0.65, 0.70, 0.75, 0.80, 0.85, 0.90, 0.95, 1.00 (Y_a^0) were used. The value returned by this calculation is $-\Delta G_{\mathrm{charge}}$.

For each value of λ, the starting configuration was minimized using a steepest-descent algorithm for 10000 steps with a cutoff distance of 12 Å for both Lennard-Jones and electrostatic interactions. The simulation box was equilibrated in the NpT ensemble for 1 ns. Using the Berendsen barostat[1] with a relaxation time of 2.0 ps, the average system pressure was kept at 1.0 bar. Also, in this 1 ns equilibration step, using a Langevin thermostat with a coupling constant of 5.0 ps^{-1} the simulation box was heated for 250 ps between 0 and 298 K, after which the average temperature of the system was kept at 298 K. Lennard-Jones interactions and direct electrostatic interactions were calculated up to a cutoff of distance 10 Å. Beyond this cutoff, electrostatic interactions were calculated with the Particle Mesh Ewald (PME) algorithm[2] with a grid spacing of 1 Å, and fourth order interpolation. Long-range dispersion corrections were applied to both the energy and pressure. The protein backbone atoms (N, C_α, C, O) were restrained to their initial positions using a harmonic restraint with a force constant of 35 kcal·mol^{-1}·Å^{-2}. The value of *skinnb* was increased to 5 Å. The production phase of the simulation was performed in the NVT ensemble and lasted 10 ns, using the Langevin thermostat with a coupling constant of 5.0 ps^{-1} to keep the average temperature at 298 K. The cutoff value for the calculation of Lennard-Jones and direct electrostatic interactions was increased to 12 Å.

Soft-core potentials were used for the residues participating in the mutation, taking the AMBER default

parameter values of α=0.5 and β=12.0 Å^2 which control the softness of this potential. The SHAKE algorithm was used to constrain the length of bonds involving hydrogen atoms, except those in the two residues involved in the mutation. We used a time step of 1 fs in all the simulations.

S2.1.1 Evaluating the quality of the free energy calculations

In Figure S2 we show the result of the integrations for the mutation (D81)...D83N on the surface of 1DOI protein at b_{KCl} = 2 mol·kg^{-1} concentration. This figure shows the vdW (Figure S2a), charge (Figure S2b), and decharge (Figure S2c) curves of integration of $\langle \partial U(\lambda)/\partial\lambda \rangle_{\lambda_i}$ versus λ_i values. Usually, the integration for the charge step is smooth. In contrast, there is a sharp change around $0.65 < \lambda < 0.78$ in the decharge step, so we used a $\Delta\lambda$ of 0.01 in this region to be able to capture the change more accurately. As for the vdW step, the integration is not always very smooth. We did not increase the number of λ values for this step because the results show (see Tables S4,S6,S7 and S5) that it contributes minimally to the total free energy change of the mutation.

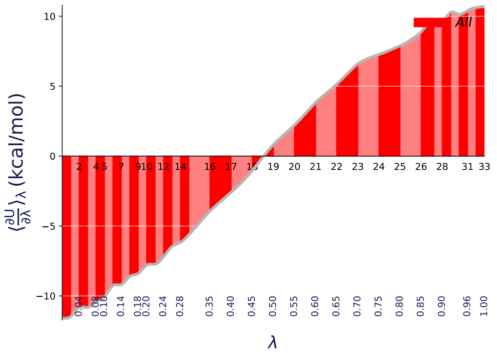

(a) vdW

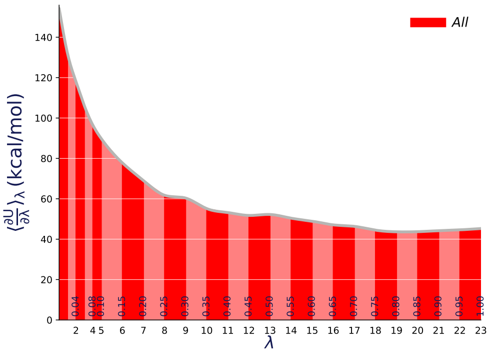

(b) charge

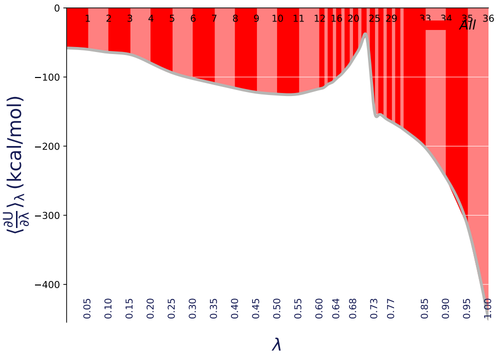

(c) decharge

Fig. S2 Plot of $\left\langle \frac{\partial U}{\partial \lambda} \right\rangle_\lambda$ vs. λ values for the thermodynamic integration corresponding to the (D81)...D83N mutation at b_{KCl} = 2 mol·kg^{-1} of protein 1DOI. The light and dark red filled areas indicate free energy estimates from the TI-1 method; the silver curve indicates interpolation via the TI-3 method. The agreement between the two interpolation schemes suggests that the interpolation successfully captures the free energy change between two neighboring λ, as well as over the whole range. The subfigures correspond to the three legs of the thermodynamic cycle shown in Scheme 1 in the main text, with (a) vdW, (b) charge, and (c) decharge. Free energy values calculated with the TI-3 method: $-\Delta G_{\text{charge}} = 59.50194$ kcal·mol^{-1}; $-\Delta G_{\text{decharge}} = -133.7835$ kcal·mol^{-1}; $\Delta G_{\text{vdW}} = 0.23334$ kcal·mol^{-1}, resulting in a total free energy change of 74.5149 kcal·mol^{-1}.

S2.1.2 Calculating the standard error of the free energies

We made the assumption that the statistical error associated with the mutation free energy values is the same for every protein site we investigated. We estimated this error by calculating the Standard Error of the Mean (SEM) for one case only: the mutation (E26)...D29N of protein 1DOI at b_{KCl} = 2 mol·kg^{-1} (see also Table S4). We performed 5 independent simulations using the optimized simulation protocol described in Section S2.1. The resulting free energy values are shown in Table S1.

Table S1 Free energy values and their SEM (kcal·mol^{-1}) associated with the (E26)...D29N of protein 1DOI at b_{KCl} = 2 mol·kg^{-1}, from 5 independent simulations. These SEM values were taken as estimates of the statistical uncertainty of all free energy values reported in the main text.

Simulation	1	2	3	4	5	SEM
$-\Delta G_{\mathrm{charge}}$	56.26149	56.54705	56.56241	56.45222	56.99566	0.12
$-\Delta G_{\mathrm{decharge}}$	-132.62702	-131.24322	-133.03272	-133.62545	-134.52607	0.55
ΔG_{vdW}	-0.64023	-0.61521	-0.73233	-0.61889	-0.60338	0.02
ΔG_{XaY} (total)	75.7253	74.08096	75.73798	76.55434	76.92703	0.49

The SEM associated with each step (charge, decharge and vdW) was calculated using the following expressions:

$$\sigma = \sqrt{\frac{\sum_{i=1}^{N}(x_i - \bar{x})^2}{N-1}} \tag{1}$$

$$\mathrm{SEM} = \frac{\sigma}{\sqrt{N}} \tag{2}$$

where $N = 5$ is the number of independent simulation runs for each simulation step (charge, decharge and vdW), x_i is the free energy value of each simulation step, $\bar{x}$ is the mean over the N values, and σ is the standard deviation.

S2.2 MD of folded protein L.

The folded halophilic protein L. was simulated at b_{KCl}= 2 mol·kg^{-1} with the same parameters as for the mutation free energy simulations, to obtain a continuous trajectory with frequently saved configurations for detailed structural analysis. This simulation used the same starting configuration as well as the same minimization and equilibration protocols as for the mutation free energy calculations of this protein. In the production phase, the system was simulated in the NVT ensemble for 400 ns. The final trajectory contains 4×10^4 configurations saved every 10 ps.

S2.3 Simulation details for the potential of mean force calculations

All simulations for the PMF calculations were done using the GROMACS 2020 simulation package.[3,4] The simulation boxes were prepared by placing the side chains in appropriate positions near the center of a cubic box with an edge length $L \approx 6$ nm and then adding the appropriate number of TIP3P water molecules and potassium and chloride ions to create an aqueous solution of the desired potassium chloride molality. In all steps of the simulations, electrostatic interactions were calculated using direct summation up to 1.2 nm, and using the particle mesh Ewald[2] (PME) scheme with a grid spacing of 0.12 nm beyond this cutoff distance. Lennard-Jones interactions were smoothly shifted to zero between 1.0 nm and 1.2 nm using the switch function available in GROMACS. Long-range dispersion corrections were applied to both the energy and pressure. A leap-frog stochastic (SD) integrator[5] was used to integrate the equations of motion in all simulations. All bonds with H-atoms were restrained using the LINCS algorithm[6] in all simulation steps (except the minimization step with the l-bfgs method), which enables integration using a 2 fs time step.

The initial configurations were equilibrated as follows: i) Two initial minimization steps with the

steepest-descent and l-bfgs algorithms. The latter is a quasi-Newtonian algorithm for energy minimization, which converges faster than the Conjugate-Gradient algorithm. ii) A 500 ps heating equilibration simulation in the canonical ensemble using the Langevin thermostat with a coupling constant of 1.0 ps and using a target temperature of 298 K. iii) Another 6 ns simulation in the isothermal-isobaric ensemble to equilibrate the system density at the pressure of 1 bar, using the Berendsen barostate[1] with a relaxation time of 1.0 ps. In all of these equilibration steps, the C_αs were restrained to their initial position using a harmonic restraint with a force constant of 10000 kJ·mol^{-1}·nm^{-2}.

To generate starting configurations for the umbrella simulations, we performed pulling simulations in the isothermal-isobaric ensemble. The pressure was controlled using the Parrinello-Rahman barostat[7,8] with a relaxation time of 1.0 ps and a target pressure of 1 bar. The temperature was controlled separately for the water and the remaining species using two Nose-Hoover thermostats, each with a coupling constant of 1.0 ps and a target temperature of 298 K. The C_α of one side chain was restrained to its initial position, in the proximity of the box center, using a harmonic restraint with a force constant of 10000 kJ·mol^{-1}·nm^{-2}. For the 2-body system, the second residue was pulled along the 3-D vector associated with the distance ξ. For the 3-body system, the third residue was pulled along the 3-D vectors associated with the distances ξ_1 and ξ_2. In all cases the pulling simulations had a duration of 1.5 ns, the pull rate was 1 nm·ns^{-1}, the distance restraints were enforced with a harmonic potential with a force constant of 10000 kJ·mol^{-1}·nm^{-2}, and 6 angular restraints and 3 dihedral harmonic restraints (described in the main text) were applied to ensure that the 3 side chains were approximately parallel to each other and were perpendicular to the plane defined by the three C_α. The force constant for the angular restraints was 2000 kJ·mol^{-1}·rad^{-2} and for the dihedral restraints were 2000 kJ·mol^{-1}·rad^{-2}. The pulling simulations were post-processed using in-house python scripts to extract individual configurations where the reaction coordinates ξ, ξ_1 and ξ_2 vary between $\approx$ 2.5 Å and 15 Å in 0.5 Å steps. These configurations were used as the starting configurations of the umbrella simulations.

Each umbrella simulation lasted 60 ns. The distance, angle and dihedral reaction coordinates were restrained to their values in the starting configuration used for each umbrella simulation. The force constants for the distance restraints were 5000 kJ·mol^{-1}·nm^{-2}; for the angular and dihedral restraints, force constants of 500 kJ·mol^{-1}·rad^{-2} were used.

S3 Identifying the best computational scheme to calculate free energies of mutation

Optimized simulation protocols for mutation free energy calculations of the type of system investigated here – highly charged proteins at high salt concentration in explicit water, with mutations leading to a change in the total charge of the system, and where the accuracy and precision of the calculated values are critical for the desired study – have not been reported. To address this issue, we investigated how the number of intermediate states, conformational sampling adequacy, finite-size effects in free energy calculation and post-processing free energy calculation methods impact the results. In the computational test studies that follow, the simulation settings were those used in the final simulations unless explicitly noted otherwise.

The calculations of the free energy associated with an amino acid mutation are done using the 3-step protocol described in Scheme 1 in the main text: the charge, decharge, and vdW steps are performed separately. Mutation free energies could, in principle, have been calculated using a one-step protocol corresponding to the direct mutation of residue, ΔG_{XaY}, in Scheme 1, by applying softcore potentials to the vdW and electrostatic interactions. We opted not to do so because Garton et al.[9] have concluded in their study that, for mutations leading to a change in the electric charge, the 3-step protocol yields more accurate free energy values than the 1-step protocol. They have also indicated that if a change in charge with the mutation does not occur, the 3-step protocol should always be avoided.

S3.1 Finite-size effects

Mutation free energies involving charge changes and in explicit solvent might suffer from significant finite-size effects[10]. Finite-size effects occur when the result of a simulation in a periodic boundary box and that done in an infinite bulk medium differ. They arise primarily because of the treatment of long-range electrostatic interactions in simulations. When performing simulations in periodic boxes, finite-size effects have two main sources: firstly, the extra electrostatic interaction between the solute in the computational reference box, its periodic replicas, and the homogeneous background charge density; and secondly, the undersolvation of the solute in the reference box because the solvent in the periodic boxes is perturbed by the image of the solute in the respective box, and is therefore unavailable for the solute in the main box[10]. Analytical and numerical schemes have been proposed[10–13] to correct these issues, but not all commonly used simulation packages have implemented them. To check how finite size effects impact the results of free energy calculations in AMBER[14] when using Particle Mesh Ewald (PME)[15] to calculate long-range electrostatics, we calculated the solvation free energy (ΔG_{solv}) of one potassium ion in a box of TIP3P water, for different box sizes. The production simulation was performed after minimization and heating; it used a timestep of 2 fs and lasted 10 ns. The SHAKE algorithm was used for every bond involving hydrogen. The average temperature was kept at 298 K using the Langevin thermostat with a coupling constant of 2 ps^{-1}, and the average pressure was kept at 1 bar using the Berendsen barostat with a coupling constant of 2 ps. A distance cutoff of 8 Å was used for the van der Waals interactions and for the direct calculation of electrostatics interactions. The potassium ion was transferred to the gas phase using a one-step protocol. In total 51 λ values of 0.00 (ion fully coupled to the solution), 0.02, 0.04, 0.06, 0.08, 0.10, 0.12, 0.14, 0.16, 0.18, 0.20, 0.22, 0.24, 0.26, 0.28, 0.30, 0.32, 0.34, 0.36, 0.38, 0.40, 0.42, 0.44, 0.46, 0.48, 0.50, 0.52, 0.54, 0.56, 0.58, 0.60, 0.62, 0.64, 0.66, 0.68, 0.70, 0.72, 0.74, 0.76, 0.78, 0.80, 0.82, 0.84, 0.86, 0.88, 0.90, 0.92, 0.94, 0.96, 0.98, 1.00 (ion fully decoupled from solution (gas phase)) was used.

The $\langle \partial U/\partial \lambda \rangle_\lambda$ vs. λ plots for all the four different box sizes as well as the values of $-\Delta G_{\mathrm{solv}}$ are shown in Figure S3. We find that the solvation free energy values vary between -93.8 and -94.6 kcal·mol^{-1} for boxes with edge-length varying between 45 and 70 Å. Moreover, they do not vary monotonically with edge-length. The results confirm that the Amber 18 code has the appropriate correction schemes in place and thus that our calculated mutation free energy values have minimal influence from finite-size effects.

We further note that, because we used the same box size for each protein and each salt concentration

in all the mutation free energy calculations reported in the main text, differences between the reported values cannot be due to finite-size effects.

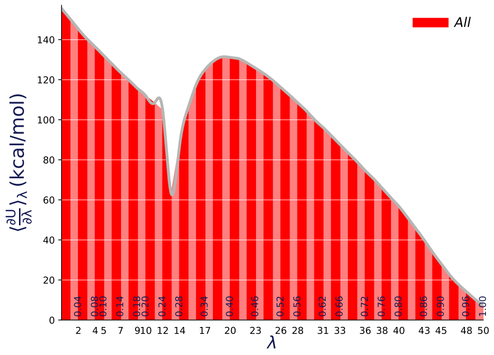

(a) Box-edge length ≈ 70 Å. Total free energy 93.78527 kcal·mol^{-1}.

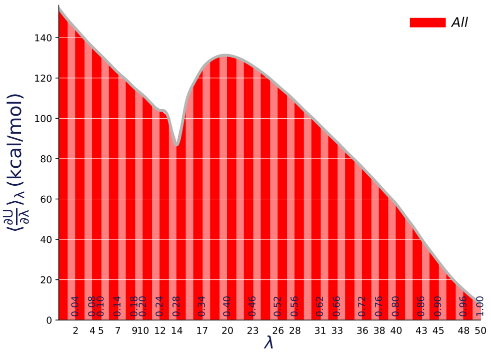

(b) Box-edge length ≈ 60 Å. Total free energy 94.56233 kcal·mol^{-1}.

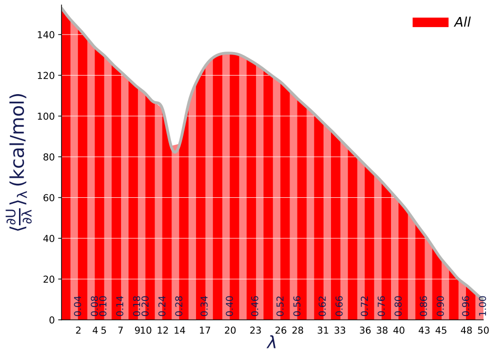

(c) Box-edge length ≈ 50 Å. Total free energy 94.30012 kcal·mol^{-1}.

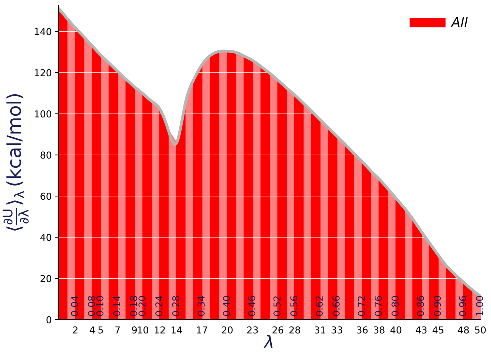

(d) Box-edge length ≈ 45 Å. Total free energy 94.50033 kcal·mol^{-1}.

Fig. S3 Plot of $\left\langle \frac{\partial U}{\partial \lambda} \right\rangle_\lambda$ vs. λ values for the integration. This figure shows the mutation corresponding to annihilating a potassium ion. The details of the simulation are explained in Subsection S3.1.

S3.2 Simulation time, number of intermediate states and post-processing method

To assess whether 10 ns of simulation time for each λ enabled sufficient sampling, we compared the final free energy associated with the mutation (D81)...D83N of protein ferredoxin (pdb ID: 1DOI) at b_{KCl} = 2 mol·kg^{-1} (compare also with Figure S2) against that obtained from simulations that used 30 ns of simulation time for each λ. All the simulation details are the same as the final free energy simulations, except that the SHAKE algorithm was not used for the heating and production runs. Figures S4 and S5 show the $\langle \partial U/\partial \lambda \rangle_\lambda$ vs. λ for the two cases. The results confirm that the shape of the curves is very similar in both cases, indicating that 10 ns is sufficient simulation time to sample the system at each value of λ.

To assess whether a total number of 95 λ (34 for the vdW step, 37 for decharge, and 24 for charge) enabled us to capture the shape of the $\langle \partial U/\partial \lambda \rangle_\lambda$ vs. λ curves with sufficient definition, we performed another test simulation where the total number of λ was increased dramatically to 177: 59 λ values of 0.00, 0.01, 0.02, 0.03, 0.04, 0.05, 0.06, 0.08, 0.12, 0.14, 0.16, 0.20, 0.24, 0.28, 0.32, 0.36, 0.40, 0.44, 0.48, 0.52, 0.56, 0.58, 0.60, 0.62, 0.64, 0.65, 0.66, 0.67, 0.68, 0.70, 0.71, 0.72, 0.73, 0.74, 0.75, 0.76, 0.77, 0.78, 0.79, 0.80, 0.81, 0.82, 0.83, 0.84, 0.85, 0.86, 0.87, 0.88, 0.89, 0.90, 0.91, 0.93, 0.94, 0.95, 0.96, 0.97, 0.98, 0.99, 1.00 for each of the vdW, decharge and charge steps. The simulation details were precisely the same as those of the previous simulations described in this subsection, with a 10 ns of total production simulation time. Figure S6 shows the integration curves corresponding to the different steps of this simulation. The curves have very similar shapes in both cases, confirming that 95 values of λ are sufficient to accurately estimate the free energies.

Table S2 quantitatively compares the results of these simulations by processing the simulation data using an integration-based method (TI-3) and using a perturbation-based method (BAR). Integration methods are sensitive to the smoothness of the $\langle \partial U/\partial \lambda \rangle_\lambda$ curve; in contrast, perturbation-based methods are sensitive to the extent of phase space overlap between adjacent λ windows. Agreement between the free energy values calculated with both methods suggests that the simulations adequately sampled the system and thus that the results are reliable. The values obtained with TI-3 and with BAR are almost identical for every case. Increasing the simulation time per λ alters the total free energy values by less than 0.1 kcal·mol^{-1}, confirming that there is no advantage in increasing the simulation time beyond 10 ns. Increasing the number of λ values altered the total free energy by 0.65 kcal·mol^{-1}, i.e., only slightly higher than the estimated SEM (section S2.1.2) of $\pm$0.49 kcal·mol^{-1} associated with the free energy values.

Table S2 Free energy values (kcal·mol^{-1}) calculated from different perturbation-based (BAR), and integration-based (TI-3) methods, for the indicated simulation conditions (number of λ values and simulation time per λ value).

		decharge		charge		vdW		total	
	Simulation time (ns)	TI-3	BAR	TI-3	BAR	TI-3	BAR	TI-3	BAR
**95λ**	10 (fig. S4)	-135.79401	-135.49497	60.04460	59.89161	0.15400	0.19775	75.90341	75.80111
**95λ**	30 (fig. S5)	-135.33353	-135.38722	59.66333	59.65385	0.13958	0.17432	75.80978	75.90769
**177λ**	10 (fig. S6)	-134.75056	-134.36508	59.76601	59.73252	0.27122	0.25510	75.25577	74.88766

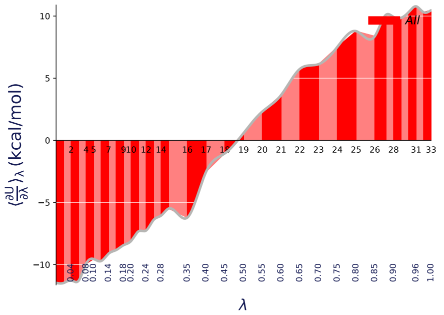

(a) vdW

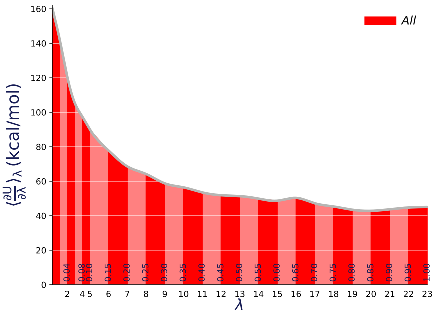

(b) charge

images/dhdl_TI_decharge10ns.pdf

(c) decharge

Fig. S4 Plot of $\left\langle \frac{\partial U}{\partial \lambda} \right\rangle_\lambda$ vs. λ values for the (D81)...D83N mutation of protein 1DOI at b_{KCl} = 2 mol·kg^{-1}, with 10 ns of simulation time per λ. The details of the simulation are explained in Subsection S3.2.

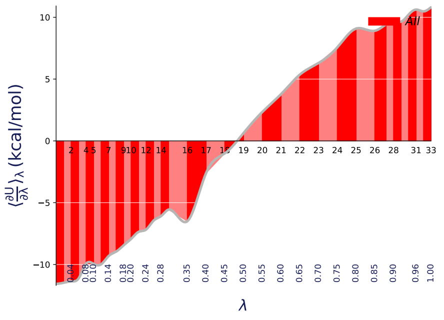

(a) vdW

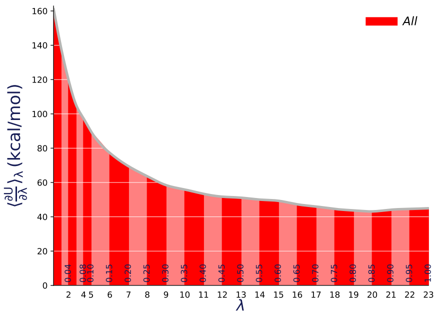

(b) charge

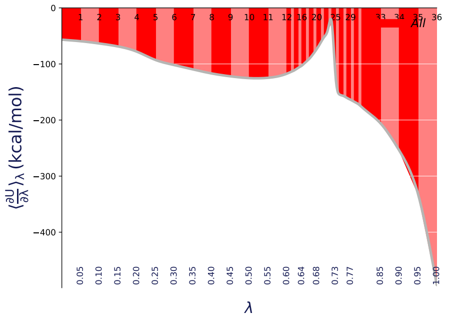

(c) decharge

Fig. S5 Plot of $\left\langle \frac{\partial U}{\partial \lambda} \right\rangle_\lambda$ vs. λ values for the (D81)...D83N mutation of protein 1DOI at b_{KCl} = 2 mol·kg^{-1}, with 30 ns of simulation time per λ. The details of the simulation are explained in Subsection S3.2.

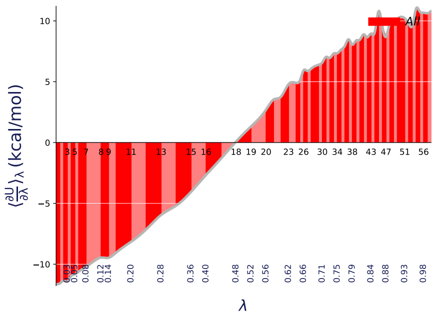

(a) vdW

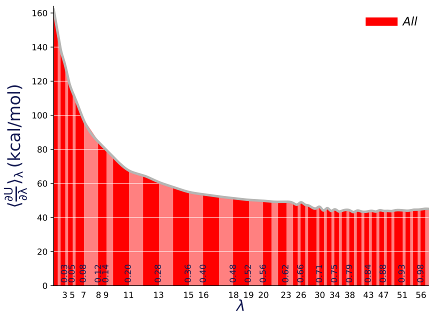

(b) charge

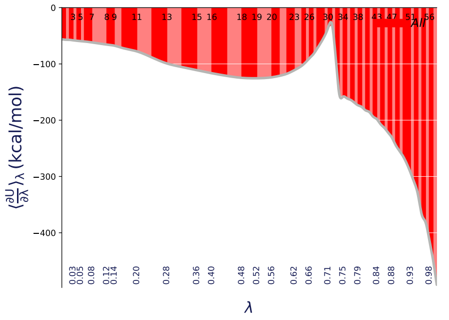

(c) decharge

Fig. S6 Plot of $\left\langle \frac{\partial U}{\partial \lambda} \right\rangle_\lambda$ vs. λ values for the (D81)...D83N mutation of protein 1DOI at b_{KCl} = 2 mol·kg^{-1}, with a total of 177 λ. The details of the simulation are explained in Subsection S3.2.

S3.3 Restraining the backbone of proteins

We calculated the free energy associated with the D34N mutation of protein 1DOI at b_{KCl} = 2 mol·kg^{-1}, with and without backbone restraints. For each value of λ the system was heated from 0 to 298 K over 300 ps with a timestep of 1 fs, using a Langevin thermostat with a coupling constant 1 ps^{-1}. A harmonic restraint with a force constant of 10 kcal·mol^{-1}·Å^{-2} on all heavy atoms of the protein was used during the heating stage; the SHAKE algorithm was not applied. After the target temperature 298 K was reached, the average temperature was kept constant at that value. The production simulation lasted 30 ns. A timestep of 2 fs was used because the SHAKE algorithm was used for all the bonds connected to the hydrogen atoms, including the mutating residues. The Langevin thermostat was used to keep the average temperature at 298 K using a coupling constant of 2 ps^{-1}, and the Berendsen barostat with a coupling constant of 2 ps kept the average pressure at 1 bar. The distance cutoff for the calculation of Lennard-Jones interactions and for the direct calculation of electrostatic interactions was 12 Å. For one simulation, harmonic restraints with bond constants of 50 kcal·mol^{-1}·Å^{2} were applied to the backbone atoms N, C$_{\alpha}$, C, and O; for the other, the restraints were turned off. In total 26 λ values of 0.00 (X), 0.04, 0.08, 0.12, 0.16, 0.20, 0.24, 0.28, 0.32, 0.36, 0.40, 0.44, 0.48, 0.52, 0.56, 0.60, 0.64, 0.68, 0.72, 0.76, 0.80, 0.84, 0.88, 0.92, 0.96, 1.00 (Y) corresponding to direct mutation in one step instead of three, in the scheme 1 were used.

The two cases are compared in Table S3 and in Figures S7a and S7b. Restraining the backbone resulted in a $\langle \partial U/\partial\lambda \rangle_{\lambda}$ vs. λ curve that is smoother and easier to integrate; it also resulted in free energy estimates that are much more similar between TI-3 and BAR than when restraints were not applied. The absence of restraints enables small conformational changes unrelated to the mutation but which affect the mutation free energy by a few kcal·mol^{-1}. Producing free energy values that average over all the small conformational changes is computationally prohibitive. Because this variation would make it impossible to make the detailed comparisons of free energy values that are at the core of this study, the protein backbone was restrained in the final free energy calculations.

Table S3 Free energy values (kcal·mol^{-1}) associated with the D34N mutation of the protein 1DOI at b_{KCl} = 2 mol·kg^{-1}, calculated from different perturbation-based (BAR) and integration-based (TI-3), from simulations where the backbone was restrained or free.

Backbone	TI-3	BAR
Restrained (fig. S7a)	72.75423	73.61249
Free (fig. S7b)	76.03225	73.29420

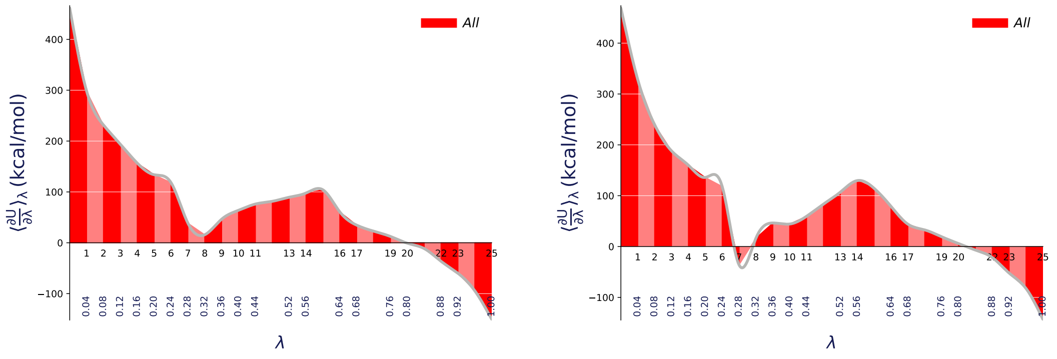

(a) Backbone restrained **(b)** Backbone free

Fig. S7 Plot of $\left\langle \frac{\partial U}{\partial \lambda} \right\rangle_{\lambda}$ vs. λ values for the D34N mutation of protein 1DOI at b_{KCl} = 2 mol·kg^{-1}. The details of the simulation are explained in Subsection S3.3.

S4 Free energy of mutations in protein L., ferredoxin, and dihydrofolate reductase

Table S4 Free energies (kcal·mol^{-1}) of mutation for the halophilic protein ferredoxin. The standard error of the mean (SEM) calculated as described in S2.1.2 is reported for each value. $\Delta G_{XaY} = \Delta G_{decharge} + \Delta G_{vdW} + \Delta G_{charge}$; see Scheme 1.

	b_{KCl} = 2 mol·kg^{-1}					b_{KCl} = 0.15 mol·kg^{-1}				
**mutation**	-ΔG_{charge} (±0.1)	-$\Delta G_{decharge}$ (±0.6)	ΔG_{vdW} (±0.02)	ΔG_{XaY} [a] (±0.5)	distance [b]	-ΔG_{charge} (±0.1)	-$\Delta G_{decharge}$ (±0.6)	ΔG_{vdW} (±0.04)	ΔG_{XaY} (±0.5)	distance (Å)
(D12)...D13N	60.76736	-137.79309	-0.55357	76.47216	5.63	63.24650	-140.28636	-0.56827	76.47159	5.96
(N12)...D13N	59.74974	-138.17301	-0.50017	77.9231	5.53	62.57828	-141.54472	-0.60791	78.35853	5.94
(E26)...D29N	56.26149	-132.62702	-0.64023	75.7253	6.36	57.82612	-133.13112	-0.09011	75.21489	5.19
(Q26)...D29N	56.11569	-133.70953	-0.60941	76.98443	7.74	56.45076	-132.36193	-0.09076	75.82041	5.41
(D81)...D107N	63.03267	-141.40526	-1.55017	76.82242	4.36	61.32781	-139.49030	-1.58711	76.57538	5.32
(N81)...D107N	62.65884	-141.83911	-1.64680	77.53347	5.58	61.34764	-140.34335	-1.78861	77.2071	5.23
(D83)...D81N	63.83078	-139.56623	0.32738	76.06283	10.97	66.46505	-142.27500	-0.85744	74.95251	6.04
(N83)...D81N	63.70651	-139.70081	0.25179	76.24609	9.86	66.10691	-143.31791	-0.93470	76.2763	4.84
(D109)...E110Q	57.22122	-135.08841	-0.59288	77.27431	5.53	57.41629	-134.64142	-0.33888	76.88625	7.65
(N109)...E110Q	56.89173	-134.29882	-0.75998	76.64711	6.74	57.98058	-136.46016	-0.10906	78.37052	8.50

[a] Combination of all thermodynamic cycles' free energy, and multiplying the values of $-\Delta G_{charge}$, and $-\Delta G_{decharge}$ part by a negative to fit the scheme 1 with the associated value of standard error of the mean. [b] The average distance between the terminal carbons of the side-chain of the mutating residue, D or E, and that of the neighboring residue, D, E, N, Q.The average is taken from the $-\Delta G_{vdW}$ step of the mutation simulation at λ zero.

Table S5 Free energy (kcal·mol^{-1}) of mutation of aspartic acid to asparagine for halophilic protein L. The standard error of the mean (SEM) calculated as described in S2.1.2 is reported for each value.

	b_{KCl} = 2 mol·kg^{-1}					b_{KCl} = 0.15 mol·kg^{-1}				
**mutation**	-ΔG_{charge} (±0.1)	-$\Delta G_{decharge}$ (±0.6)	ΔG_{vdW} (±0.04)	ΔG_{XaY} (±0.5)	distance	-ΔG_{charge} (±0.1)	-$\Delta G_{decharge}$ (±0.6)	ΔG_{vdW} (±0.04)	ΔG_{XaY} (±0.5)	distance (Å)
(E2)...E3Q	60.94070	-140.71040	-0.54365	79.22605	9.95	57.32818	-141.04686	-0.46305	83.25563	8.93
(Q2)...E3Q	61.10848	-139.17715	-0.35378	77.71489	9.97	57.41838	-141.39776	-0.36833	83.61105	9.58
(E28)...E32Q	65.31491	-142.93726	-0.33877	77.28358	7.87	66.00519	-141.92388	-0.46811	75.45058	8.07
(Q28)...E32Q	65.48402	-143.10207	-0.20677	77.41128	7.55	65.05565	-143.16430	-0.65581	77.45284	9.12
(E41)...E42Q	64.44933	-142.49745	-0.32551	77.72261	8.85	63.36859	-142.30051	0.02803	78.95995	7.67
(Q41)...E42Q	63.29743	-142.96200	-0.47503	79.18954	8.71	63.41466	-143.84089	-0.30592	80.12031	8.56
(D38)...E41Q	71.77454	-149.83710	-0.93222	77.13034	5.22	70.41324	-148.54546	-0.43475	77.69747	6.62
(N38)...E41Q	70.65305	-147.07521	-0.95782	75.46434	4.73	69.17052	-146.88446	-0.69658	77.01736	5.94
(E42)...D43N	57.20438	-135.56766	-0.67833	77.68495	6.17	57.07769	-134.68398	-0.43039	77.1759	6.28
(Q42)...D43N	56.67696	-134.85054	-0.46103	77.71255	6.00	57.89925	-137.23697	-0.34339	78.99433	5.76
(E23)...E21Q	60.58391	-136.00383	-1.04906	74.37086	8.57					
(Q23)...E21Q	60.04999	-134.23224	-0.54084	73.64141	7.87					
(E61)...D50N	59.29745	-138.22341	-0.93365	77.99231	6.96					
(Q61)...D50N	58.93284	-137.70456	-1.10447	77.66725	6.21					
(E28)...E27Q	66.99108	-142.59274	-0.22245	75.37921	6.30					
(Q28)...E27Q	62.82825	-140.17936	-0.17533	77.17578	7.15					

Table S6 Free energy (kcal·mol^{-1}) of mutation of aspartic acid to asparagine for halophilic protein Dihydrofolate reductase. The standard error of the mean (SEM) calculated as described in S2.1.2 is reported for each value.

$b_{\mathbf{KCl}}$ = 2 mol·kg^{-1}					
**mutation**	-ΔG_{charge} (±0.1)	-$\Delta G_{\text{decharge}}$ (±0.6)	ΔG_{vdW} (±0.04)	$\Delta G_{\text{X}a\text{Y}}$ (±0.5)	distance
(D18)...E20Q	56.77630	-130.71713	-0.52123	73.4196	5.95
(N18)...E20Q	56.41874	-133.81749	-0.75389	76.64486	9.88
(D54)...D55N	56.58246	-136.05554	-0.73275	78.74033	7.23
(N54)...D55N	55.64233	-135.87124	-0.70413	79.52478	6.46
(E133)...D135N	66.45264	-145.07489	-0.34306	78.27919	7.90
(Q133)...D135N	66.52757	-143.00993	-0.31854	76.16382	8.11
(D135)...E138Q	69.22918	-146.38492	-0.30062	76.85512	5.78
(N135)...E138Q	68.29959	-146.02664	-0.27535	77.4517	5.26
(E144)...D146N	57.65902	-131.85082	-0.35306	73.83874	6.11
(Q144)...D146N	57.69196	-133.70504	-0.23425	75.77883	5.53

Table S7 Free energy (kcal·mol^{-1}) of mutation of Aspartic acid to Asparagine for unfolded halophilic protein L. The standard error of the mean (SEM) calculated as described in S2.1.2 is reported for each value.

$b_{\mathbf{KCl}}$ = 2 mol·kg^{-1}					
**mutation**	-ΔG_{charge} (±0.1)	-$\Delta G_{\text{decharge}}$ (±0.6)	ΔG_{vdW} (±0.04)	$\Delta G_{\text{X}a\text{Y}}$ (±0.5)	distance
(E2)...E3Q	61.22625	-140.86904	-0.14001	79.50278	9.38
(Q2)...E3Q	62.13673	-141.91212	-0.27358	79.50181	7.44
(E21)...E23Q	56.39029	-137.91575	-0.06632	81.45914	7.50
(Q21)...E23Q	57.07932	-137.98829	-0.97050	79.93847	8.21
(E41)...E42Q	64.36035	-139.94819	0.04432	75.63216	6.16
(Q41)...E42Q	63.92586	-140.09838	-0.30096	75.87156	5.86
(E41)...D43N	61.78881	-135.53975	-0.27822	73.47272	10.64
(Q41)...D43N	62.42679	-133.52279	-0.46981	70.62619	9.62
(D43)...E46Q	61.07022	-139.94670	-0.16233	78.71415	5.96
(N43)...E46Q	60.45539	-136.06756	0.12897	75.74114	6.80

S5 Negative values of $\Delta\Delta G$ originate from interactions between vicinal acidic amino acids

A negative value of $\Delta\Delta G_{\text{decharge}}$ means that decharging an acidic amino acid, X, is more unfavorable when the neighbor is also an acidic amino acid (Equation 3):

$$(-)\text{X}_a \xrightarrow{\Delta G_{(-)\text{decharge}}} (-)\text{X}_a^0 \tag{3}$$

that when the neighbor is electrically neutral (Equation 4):

$$(0)\text{X}_a \xrightarrow{\Delta G_{(0)\text{decharge}}} (0)\text{X}_a^0. \tag{4}$$

Therefore, a negative value of $\Delta\Delta G_{\text{decharge}}$ can arise either from i) unexpected *stabilizing* electrostatic interactions between the two vicinal acidic amino acids – initial state in Equation 3 – or ii) *destabilizing* electrostatic interactions between vicinal acidic and neutral amino acids – initial state in Equation 4 – strong enough to compensate the expected electrostatic repulsion between two negative amino acids.

Indirect insight from the charging step indicates that the first possibility is the one at play here. The charging step indicates it is more favorable to introduce atomic charges in the final amino acid Y (which is net neutral) when its neighbor has a negative charge, corresponding to Equation 5,

$$(-)\text{Y}_a^0 \xrightarrow{\Delta G_{(-)\text{charge}}} (-)\text{Y}_a \tag{5}$$

than when its neighbor is neutral, corresponding to Equation 6

$$(0)\,\text{Y}_a^0 \xrightarrow{\Delta G_{(0)\text{charge}}} (0)\,\text{Y}_a \tag{6}$$

Considering that i) the electrostatic interaction between two neutral amino acids is well-described as a dipole-dipole interaction; ii) this interaction decays as $1/r^3$ where r is the distance between the dipoles; iii) the amino acids in question are at least 5 Å apart; iv) we are considering interactions at high KCl concentrations, we can assume that the electrostatic energy between 2 neutral amino acids, corresponding to the "products" in Equation 6, is approximately zero; our PMF calculations confirm that this assumption holds. Consequently, $-\Delta\Delta G_{\text{charge}} \approx \Delta G_{(-)\text{charge}}$ is a reasonable estimate of the electrostatic interaction between a neutral (N or Q) and an acidic (D or E) amino acid; this value is negative, indicating they have stabilizing interactions. These results indicate that synergistic effects between neighboring acidic amino acids, as measured by $\Delta\Delta G$, reflect electrostatically favorable interactions between vicinal acidic amino acids.

S6 Replica Exchange Molecular Dynamics (REMD)

S6.1 Computational details of REMD simulation

We performed a REMD simulation to sample the unfolded state ensemble of halophilic protein L. (pdb ID: 2KAC) at the high salt concentration of $b_{\text{KCl}} = 2$ mol·kg^{-1}. Our aim was to obtain a representative unfolded configuration of this protein for subsequent free energy studies of the synergistic effect, to enable comparisons between the folded and the unfolded states.

The simulations were performed using the AMBER 2018 simulation package[14], using the pmemd engine on GPUs. The only exception was the l-bfgs minimization step, which was performed on the Sander engine of the AMBER simulation package because it is not available on the pmemd engine. All the simulation boxes are cubic with periodic boundary conditions applied in the XYZ directions. We set a non-bonded potential cutoff distance at 12 Å for vdW and electrostatic interactions. Beyond this cutoff distance, the electrostatic interactions are calculated with the particle mesh Ewald (PME) scheme with a grid spacing of 1.0 Å, and 4th order of interpolation[2]. Long-range dispersion corrections were applied to both the energy and pressure. The crystal structure of the halophilic protein with pdb ID 2KAC was placed in a simulation box with an edge length of $\approx$ 110 Å; the box was filled with TIP3P water and sufficient ions to obtain an electrically neutral system with the desired molality of KCl. All bonds with H-atoms were constrained using the SHAKE algorithm[16] in the NpT ensemble and REMD simulation. Four initial minimizations cycles, each with 2500 steps of the Steepest-Descent algorithm and 7500 steps of the Conjugate Gradient algorithm while using progressively weaker harmonic restraints (with bond constants 500, 300, 100, and 50 kcal·mol^{-1}· Å^{-2}) on the protein atoms were performed to remove the bad contacts that arose from the experimental structure and in the process of adding ions and water to the system. Afterwards, another minimization using the l-bfgs algorithm without any constraints or restraints was performed for 10000 steps.

The system was heated over 50 ns in the canonical ensemble (NVT) using the Langevin thermostat with a collision frequency of 1.0 ps^{-1} to slowly increase the temperature of the system from 0 to 800 K to denature the protein. Then another heating simulation in the canonical ensemble (NVT) was performed for 2.5 ns, during which the temperature was decreased again to reach 298 K using the Langevin thermostat with the same collision frequency. The SHAKE algorithm was not used for these two heating simulations, so consequently, a timestep of 1 fs was used. The system was equilibrated for a total of 10 ns, in 10 steps of 1 ns simulations, to equilibrate the density in the isothermal-isobaric ensemble (NpT) using the Berendsen barostat[1], and the Langevin thermostat, to keep the average temperature at 298 K, and the pressure at 1 bar.

The REMD simulation was performed in the NVT ensemble. The starting configuration was extracted from the last 1 ns NpT equilibration simulation, taking care to choose a configuration in which the density of the box was closest to the average. Also for the REMD simulation, we used the Langevin thermostat with a collision frequency of 1 ps^{-1}. The replica temperatures are given in section S6.2. Replica exchanges were attempted every 2500 MD steps; a total of 20000 exchanges were attempted. Consequently, each replica was simulated for $2500 \times 20000 = 5 \times 10^7$ MD steps which, considering the timestep of 2 fs, results in 100 ns of simulation time for each replica.

At such high temperatures, (> 500 K) unwanted rotations around the peptide bond might occur, leading to non-physical chiralities. To prevent this, we used harmonic chirality restraints with force constants of 50 kcal·mol^{-1}·Å^{-2} on the backbone ω dihedrals (defined by the backbone atoms C_α, C, N, C_α), to retain it in *trans* configuration to keep it planar, and do not allow its rotation to *cis* configuration.

In the end, we extracted a trajectory from only those frames of all replica trajectories that corresponded to 298 K and performed the subsequent analysis on this trajectory.

S6.2 Temperature replicas used in REMD simulation

A total of 221 replica with different temperatures was used as 298.15, 298.98, 299.82, 300.65, 301.49, 302.33, 303.18, 304.02, 304.87, 305.71, 306.56, 307.42, 308.27, 309.12, 309.98, 310.84, 311.70, 312.57, 313.43, 314.30, 315.17, 316.04, 316.91, 317.79, 318.67, 319.55, 320.43, 321.31, 322.20, 323.09, 323.97, 324.87, 325.76, 326.66, 327.55, 328.45, 329.35, 330.26, 331.16, 332.07, 332.98, 333.89, 334.81, 335.73, 336.64, 337.57, 338.49, 339.41, 340.34, 341.27, 342.20, 343.13, 344.07, 344.97, 345.91, 346.86, 347.80, 348.75, 349.69, 350.64, 351.60, 352.55, 353.51, 354.47, 355.43, 356.39, 357.36, 358.33, 359.30, 360.27, 361.24, 362.22, 363.20, 364.18, 365.17, 366.15, 367.14, 368.13, 369.12, 370.12, 371.11, 372.11, 373.12, 374.12, 375.12, 376.13, 378.16, 37.16, 380.18, 381.20, 382.22, 383.25, 384.28, 385.30, 386.34, 387.37, 388.41, 389.44, 390.48, 391.53, 392.57, 393.62, 394.68, 395.73, 396.79, 397.84, 398.90, 399.97, 401.03, 402.10, 403.18, 404.25, 405.33, 406.40, 407.48, 408.57, 409.65, 410.74, 411.83, 412.92, 414.02, 415.12, 416.22, 417.32, 418.42, 419.53, 420.64, 421.75, 422.87, 423.99, 425.11, 426.23, 427.36, 428.48, 429.62, 430.75, 431.89, 433.03, 434.17, 435.31, 436.46, 437.61, 438.76, 439.91, 441.07, 442.2, 443.39, 444.56, 445.73, 446.90, 448.07, 449.25, 450.43, 451.61, 452.79, 453.98, 455.17, 456.36, 457.55, 458.75, 459.95, 461.15, 462.36, 463.57, 464.78, 466.00, 467.21, 468.44, 469.66, 470.88, 472.11, 473.35, 474.57, 475.81, 477.05, 478.29, 479.54, 480.79, 482.04, 483.29, 484.55, 485.81, 487.07, 488.34, 489.61, 490.88, 492.15, 493.43, 494.71, 495.99, 497.28, 498.57, 499.86, 501.16, 502.46, 503.76, 505.06, 506.37, 507.68, 508.99, 510.31, 511.63, 512.95, 514.28, 515.61, 516.94, 518.27, 519.61, 520.95, 522.29, 523.64, 524.99, 526.35, 527.70, 529.06, 530.43, 531.79, 533.16, 534.53, 535.91, 537.00. This means a total of 22.1 μs of simulation. This temperature distribution was taken from the website `http://folding.bmc.uu.se/remd-temperature-generator/`[17].

S6.3 Evaluating the quality of REMD simulations

To examine whether a good quality REMD simulation was achieved with the temperature distribution, the number of replicas, and the simulation details used in our study, we examine the acceptance rate of exchanges between adjacent pairs, and the temperature of individual replicas. Figure S8 shows the acceptance rate of the exchanges between replicas, considering up and down exchanges. The acceptance rate varies between 40% and 56%, indicating that exchanges between neighboring replicas were likely in all parts of the temperature distribution. Also, the acceptance rates are large enough (> 40%), leading to a sufficient number of exchanges during the simulation.

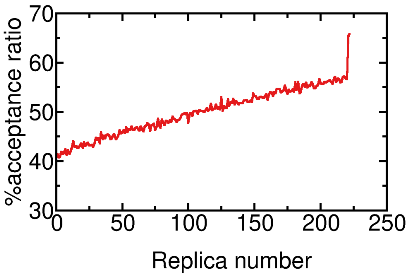

Fig. S8 Fraction of successful exchange attempts (exchange rate) for each replica.

Figure S9 shows the temperature distribution for one of the replicas with an initial temperature of 298.98 K. This replica visited higher temperatures at the end of the REMD simulation, but clearly has not experienced the desired random walk in temperature space. Doing so would require a much longer simulation time, beyond our resources.

Analysis of the protein structure (not shown) at 298 K indicates that we have sampled a subset of the unfolded structure ensemble that is rich in collapsed structures but contains few expanded structures. This level of sampling of the unfolded state ensemble is sufficient for our purpose: to obtain a structure

that is representative of the ensemble of unfolded but collapsed structures, which can be used to study the incidence of synergistic interactions between acidic amino acids in denatured protein conformations.

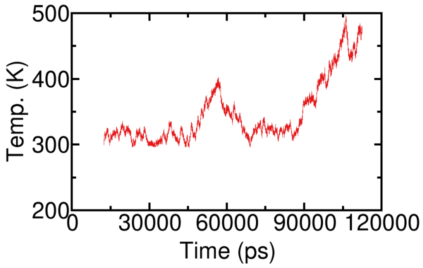

Fig. S9 Temperature distribution for the replica with the starting temperature of 298.98 K.

S7 Potential of mean force of 2-body systems

Figure S10 shows a representative configuration of two aspartates at the mininum of the PMF shown in Figure 6A in the main text. The gas phase interaction energy of the two amino acids in this conformation is negative (-2.63 kJ·mol^{-1}) because the LJ component (-5.1 kJ·mol^{-1}) largely overcomes the electrostatic repulsion (+2.44 kJ·mol^{-1}). These results confirm that the minimum of the PMF is due to the proximity of the CH_2 and CH_3 groups, which enables strong LJ attraction.

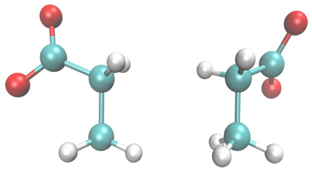

Fig. S10 Representative configuration of the D-D system with $\theta_1 = \theta_2 = 90°$ for $\xi \approx 4$ Å (the distance between the two α carbons), i.e., at the minimum of the PMF shown in Fig. 6A in the main text. The two carboxylates preferentially point away from each other whereas the CH_2 and CH_3 groups are very close.

Figure S11 shows the potential of mean force between two aspartates, calculated as a function of the distance of the carboxylate carbons. This curve differs quantitatively from that calculated based on the distance between the two alpha carbons (shown in Figure 6A) but retains similar features, such as the minimum at short separations.

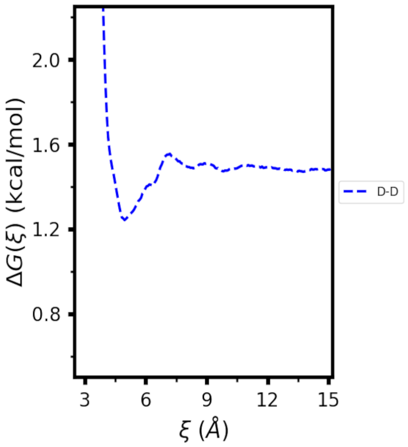

Fig. S11 Potential of mean force as a function of $C_\gamma \cdots C_\gamma$ distance of the D-D pair with parallel side chains (restraint angles $\theta_1 = \theta_2 = 90°; \phi = 0°$), at b_{KCl}= 2 mol·kg^{-1} (2m). The minimum near $\xi \approx$4 Åresults from LJ interactions, that overcome electrostatic repulsion.

S8 Potential of mean force of 3-body systems

We performed PMF calculations for a system of 3 side chains, as a function of the distance between one of the side chains from the other two. We considered only the case where the side chains are parallel to each other and perpendicular to the plane of the three C_αs, at b_{KCl} = 2 mol·kg^{-1}. We then calculated $\Delta\Delta G$ similarly as for the two-body system:

$$\Delta\Delta G(\xi) = \Delta G_{\mathrm{D-D--D}} + \Delta G_{\mathrm{D-N--N}} - 2 \times \Delta G_{\mathrm{D-D--N}} \tag{7}$$

Figure S12 shows $\Delta\Delta G(\xi)$ for the 3-body system. The interactions remain qualitatively similar to those in the analogous 2-body system, with maximum repulsion being observed for a triplet of negative charges at $\xi = 6$ Å. The value of $\Delta\Delta G(\xi)$ is never negative, indicating the synergistic effect does not occur in this 3-body system either.

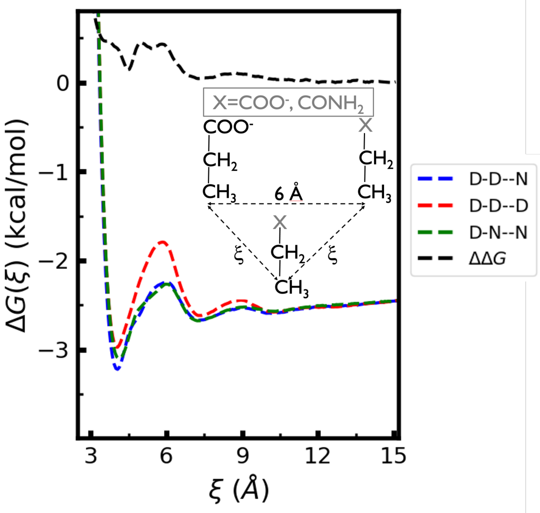

Fig. S12 Potential of mean force ($\Delta G(\xi)$) as a function of the distance ξ between a side chain and two other side chains at fixed positions, for the indicated side chain systems at b_{KCl} = 2 mol·kg^{-1} (2m).

References

1 H. J. C. Berendsen, J. P. M. Postma, W. F. van Gunsteren, A. DiNola and J. R. Haak, *J. Chem. Phys.*, 1984, **81**, 3684–3690.

2 T. Darden, D. York and L. Pedersen, *J. Chem. Phys.*, 1993, **98**, 10089–10092.

3 H. J. C. Berendsen, D. van der Spoel and R. van Drunen, *Comput. Phys. Commun.*, 1995, **91**, 43–56.

4 D. Van Der Spoel, E. Lindahl, B. Hess, G. Groenhof, A. E. Mark and H. J. C. Berendsen, *J. Comput. Chem.*, 2005, **26**, 1701–1718.

5 W. F. van Gunsteren and H. J. C. Berendesen, *Mol. Simul.*, 1988, **1**, 173–185.

6 B. Hess, H. Bekker, H. J. C. Berendsen and G. E. M. Fraaije, *J. Comput. Chem.*, 1997, **18**, 1463–1472.

7 M. Parrinello and A. Rahman, *J. Appl. Phys.*, 1981, **52**, 7182–7190.

8 S. Nosé and M. L. Klein, *Mol. Phys.*, 1983, **50**, 1055–1076.

9 M. Garton, C. Corbi-Verge, Y. Hu, S. Nim, N. Tarasova, B. Sherborne and P. M. Kim, *PROTEINS: Structure, Function, and Bioinformatics*, 2019, **87**, 236–244.

10 G. J. Rocklin, D. L. Mobley, K. A. Dill and P. H. Hünenberger, *J. Chem. Phys.*, 2013, **139**, year.

11 P. H. Hünenberger and J. A. McCammon, *Chem. Phys.*, 1999, **110**, 1856–1872.

12 M. A. Kastenholz and P. H. Hünenberger, *J. Phys. Chem. B*, 2004, **108**, 774–788.

13 M. A. Kastenholz and P. H. Hünenberger, *Chem. Phys.*, 2006, **124**, year.

14 D. Case, I. Ben-Shalom, S. Brozell, D. Cerutti, T. Cheatham III, V. Cruzeiro, T. Darden, R. Duke, D. Ghoreishi, M. Gilson, H. Gohlke, A. Goetz, D. Greene, R. Harris, N. Homeyer, Y. Huang, S. Izadi, A. Kovalenko, T. Kurtzman, ... and P. A. Kollman, *Amber 18*, University of California, San Francisco, 2018.

15 U. Essmann, L. Perera, M. L. Berkowitz, T. Darden, H. Lee and L. G. Pedersen, *Chem. Phys.*, 1995, **103**, 8577–8593.

16 J.-P. Ryckaert, G. Ciccotti and H. J. Berendsen, *J. Comput. Phys.*, 1977, **23**, 327–341.

17 A. Patriksson and D. Van Der Spoel, *Phys. Chem. Chem. Phys.*, 2008, **10**, 2073–2077.
